# Gradient-flow adaptive importance sampling for Bayesian leave one out cross-validation for sigmoidal classification models

**Published:** 2024-02-13

**Authors:** Joshua C. Chang, Xiangting Li, Shixin Xu, Hao-ren Yao, Julia Porcino, Carson C. Chow

**Affiliations:** 1 NIH Clinical Center, Rehabilitation Medicine, Epidemiology and Biostatistics Section, Bethesda MD, USA; 2 UCLA Department of Computational Medicine, Los Angeles CA, USA; 3 Data Science Research Center, Duke Kunshan University, Kunshan, Jiangsu, China; 4 NIH NIDDK, Laboratory of Biological Modeling, Bethesda MD, USA

## Abstract

We introduce a set of gradient-flow-guided adaptive importance sampling (IS) transformations to stabilize Monte-Carlo approximations of point-wise leave one out cross-validated (LOO) predictions for Bayesian classification models. One can leverage this methodology for assessing model generalizability by for instance computing a LOO analogue to the AIC or computing LOO ROC/PRC curves and derived metrics like the AUROC and AUPRC. By the calculus of variations and gradient flow, we derive two simple nonlinear single-step transformations that utilize gradient information to shift a model’s pre-trained full-data posterior closer to the target LOO posterior predictive distributions. In doing so, the transformations stabilize importance weights. Because the transformations involve the gradient of the likelihood function, the resulting Monte Carlo integral depends on Jacobian determinants with respect to the model Hessian. We derive closed-form exact formulae for these Jacobian determinants in the cases of logistic regression and shallow ReLU-activated artificial neural networks, and provide a simple approximation that sidesteps the need to compute full Hessian matrices and their spectra. We test the methodology on an n≪p dataset that is known to produce unstable LOO IS weights.

## 1 INTRODUCTION

In Bayesian formulations of classification problems where one uses a vector of covariates $x\in R^{p}$ to estimate the probability of an outcome labeled by y∈{0,1}, one specifies a likelihood function ℓ, where

(1) $$\begin{array}{c} y_{i}|\theta ,x_{i}\;∼\;\text{Bernoulli}\;\left(p_{i}(\theta )\right)\\ \ell \left(\theta |y_{i},x_{i}\right)\;=p_{i}{\left(\theta \right)}^{y_{i}}{\left(1-p_{i}\left(\theta \right)\right)}^{1-y_{i}}, \end{array}$$

and where $p_{i}(\theta )\equiv p\left(\theta ,x_{i}\right)$ is the predicted outcome probability for observation i, and computes the statistics of the parameter vector θ by applying Bayes rule

(2) $$\pi \left(\theta |𝒟\right)\propto \pi \left(\theta \right)\prod_{i=1}^{n}\ell \left(\theta |y_{i},x_{i}\right),$$

where π(θ) is the joint prior distribution over the parameter vector θ, corresponding to the likelihood function ℓ(θ). There are usually many plausible choices for ℓ, even within the same family of models (for example different sets of features in logistic regression) – one needs methodology to evaluate different models to inform decisions such as model selection.

One usually wishes to evaluate how a model will generalize to new data, by estimating its accuracy on data that is both unused during training and statistically independent from the training data. Simulating performance under unseen data requires either fitting the model multiple times (for k-fold cross validation, which can be expensive on large scale datasets, with large-scale models), or removing a subset of the data entirely (in the case of test/train splits).

Other general methods of assessing model generalizability exist, notably information criterion related to the Akaike Information criteria (AIC). The AIC and Bayesian variants [Gelman et al., 2014, Watanabe, 2013, Vehtari et al., 2017, Piironen and Vehtari, 2017a] are asymptotic approximations [Stone, 1977, Watanabe, 2010] of a model’s leave one out (LOO) cross validation loss. In their standard usage, these methods approximate the total log LOO likelihood for a given model/dataset, a quantity that does not directly map to ROC/PRC curves. However, similar methods can be used to compute LOO versions of these curve. The receiver operator curve (AUROC) [Hanley and McNeil, 1982] and the area under the precision recall cure (AUPRC) are familiar, interpretable, and often desirable for use in decision making processes.

In this manuscript, we motivate an adaptive importance sampling method for LOO, based on transformations derived by reduction of a given functional criterion by stepping down a gradient flow. These transformations use model-specific gradient information – we derive explicit formulae for the transformations and their Jacobian determinants in the case of common sigmoidal classification models (logistic regression and Bayesian ReLU-activated neural networks).

## 2 PRELIMINARIES

### 2.1 NOTATION

We denote vectors using bold-faced lower case symbols, and matrices using bold-faced uppercase symbols. By convention, all vectors are assumed to be column vectors unless otherwise stated. Given a matrix $W=\left(w_{ij}\right)$, the *i*-th row is denoted $w_{i}$, and *j*-th column is denoted $w_{,j}$.

We refer to the entire set of observed training data as $𝒟={\left\{\left(x_{i},y_{i}\right)\right\}}_{i=1}^{n}$. As shorthand, we denote the set of training data where the i-th observation is left out as ${𝒟}^{\left(-i\right)}=𝒟\backslash \left\{\left(x_{i},y_{i}\right)\right\}$. Expectations with respect to the posterior distribution of θ are denoted $E_{\theta |𝒟}$, and with respect to the posterior distribution of θ if observation i is left out are denoted $E_{\theta |{𝒟}^{\left(-i\right)}}$.

For a transformation T:Ω→Ω, where $\Omega \subset R^{p}$, we denote its Jacobian matrix $J_{T}=\nabla T={\left(\partial_{\alpha }T^{\beta }\right)}_{\alpha ,\beta }$ and the determinant of the Jacobian matrix ${𝒥}_{T}=\left|J_{T}\right|$. The gradient operator ∇ operating on a function $\mu \;:R^{p}\to R$ is assumed to yield a column vector, the Hessian matrix for a function μ is denoted ∇∇μ, and the Laplacian of μ is denoted $\nabla^{2}\mu$

The operator |⋅| refers to determinants when the argument is a matrix, the 2-norm when the argument is a vector, and the absolute value when the argument is a scalar.

### 2.2 IMPORTANCE SAMPLING-BASED APPROXIMATE LEAVE ONE OUT CROSS VALIDATION (IS-LOO)

Suppose that one has pre-trained a Bayesian classification model such that one is able to sample its posterior parameters $\theta_{s}\overset{\text{iid}}{∼}\pi (\theta |𝒟)$. Our objective is to use knowledge of this full-data posterior distribution to estimate how the model would behave if any single point is left out at training. One can relate the full-data model to the model with observation i left out using the Bayesian update equation

(3) $$\pi \left(\theta |𝒟\right)=\frac{\ell \left(\theta |x_{i},\;y_{i}\right)\pi (\theta |{𝒟}^{\left(-i\right)})}{\int \;\ell \left(\theta |x_{i},\;y_{i}\right)\pi (\theta |{𝒟}^{\left(-i\right)})d\theta },$$

which is a Fredholm second-kind integral equation with respect to $\pi (\theta |{𝒟}^{\left(-i\right)})$. This integral equation is in-practice difficult to solve due to the typically-high dimensionality of θ.

Rather than attempt direct inversion of Eq. 3, our starting point is the observation that

(4) $$\frac{\pi (\theta |{𝒟}^{\left(-i\right)})}{\pi (\theta |𝒟)}=\frac{E_{\theta |{𝒟}^{\left(-i\right)}}\left[\ell \left(\theta |x_{i},\;y_{i}\right)\right]}{\ell \left(\theta |x_{i},\;y_{i}\right)}\equiv \nu_{i}\left(\theta \right),$$

which provides the ratio of densities between a distribution we know (the full-data posterior π(θ∣𝒟)) and a distribution whose statistics we would like to compute (the point-wise LOO posterior $\pi (\theta |{𝒟}^{\left(-i\right)})$). To use the former in order to compute statistics of the latter we turn to importance sampling, noting that for an integrable function f,

(5) $$\begin{array}{l} E_{\theta |{𝒟}^{\left(-i\right)}}[f(\theta )]\;=\int \;f(\theta )\pi (\theta |{𝒟}^{\left(-i\right)})d\theta \\ \;=\int \;f(\theta )\frac{\pi (\theta |{𝒟}^{\left(-i\right)})}{\pi (\theta |𝒟)}\pi (\theta |𝒟)d\theta \\ \;=E_{\theta |𝒟}\left[f(\theta )\nu_{i}(\theta )\right]. \end{array}$$

Sampling $\theta_{k}\overset{\text{iid}}{∼}\pi (\theta |𝒟)$, we can approximate Eq. 5 using the Monte-Carlo integral

(6) $$E_{\theta |{𝒟}^{\left(-i\right)}}\approx \sum_{k=1}^{s}\nu_{ik}f\left(\theta_{k}\right)$$

where the coefficients $\nu_{ik}$ are known as the self-normalized importance sampling weights

(7) $$\nu_{ik}=\frac{\nu_{i}\left(\theta_{k}\right)}{\sum_{j=1}^{s}\;\;\nu_{i}\left(\theta_{j}\right)}=\frac{{\left(\ell \left(\theta_{k}|y_{i},\;x_{i}\right)\right)}^{-1}}{\sum_{k=1}^{s}\;\;{\left(\ell \left(\theta_{k}|y_{i},\;x_{i}\right)\right)}^{-1}},$$

so that the undetermined constant $E_{\theta |{𝒟}^{\left(-i\right)}}\left[\ell \left(\theta |x_{i},y_{i}\right)\right]$ cancels out. Eqs. 6, 7 define a well-known [Gelfand et al., 1992] Monte-Carlo estimator for LOO.

### 2.3 LOO-BASED METRICS

In Bayesian workflows, it is common to evaluate model generalizability by using importance sampling LOO to compute a Bayesian analogue to the Aikaike Information Criterion (AIC). The LOO information criterion (LOO-IC) takes the form

(8) $$\begin{array}{l} \text{LOO-IC}=-2\sum_{i=1}^{n}\text{log}\;E_{\theta |{𝒟}^{\left(-i\right)}}\left(\ell \left(\theta |x_{i},y_{i}\right)\right)\\ \;\approx -2\sum_{i=1}^{n}\text{log}\sum_{k=1}^{s}\nu_{ik}\ell \left(\theta_{k}|y_{i},x_{i}\right). \end{array}$$

Alternatively, for classification problems, one often desires other metrics such as an estimate of the out-of-sample area under the receiver operator curve or precision-recall curve. To compute these quantities, one simply propagates LOO estimates of the outcome probabilities

(9) $${\hat{p}}_{\text{loo},i}=E_{\theta |𝒟\left(-i\right)}\left[p_{i}\left(\theta \right)\right]\approx \sum_{k=1}^{s}\nu_{ik}p\left(\theta_{k},x_{i}\right),$$

into the relevant formulae. For instance, one may use the probabilistic interpretation of the AUROC [Wang and Guo, 2020] to motivate the direct estimator of the LOO AUROC,

(10) $$\mathrm{LOO}-\mathrm{AUROC}(p(y|x,\theta ))=\frac{\sum_{i\in {𝒟}^{0}}\sum_{j\in {𝒟}^{1}}1\left[{\hat{p}}_{\text{loo},i}\;<\;{\hat{p}}_{\text{loo},j}\right]}{\left|{𝒟}^{0}\right|\;\cdot \;\left|{𝒟}^{1}\right|},$$

where ${𝒟}^{0}$ is the set of all negative observations and ${𝒟}^{1}$ is the set of all positive observations.

### 2.4 WEIGHT STABILIZATION

Often it is the case that using the computed posterior π(θ∣𝒟) as the proposal distribution for importance sampling has slow convergence properties – the 1/ℓ importance weights are known to have large or unbounded variance [Peruggia, 1997], making the importance sampler estimate for LOO noisy.

Two practical model agnostic methods for controlling the tail of the importance weights are through weight truncation [Ionides, 2008] and Pareto smoothing [Vehtari et al., 2015, 2017]. Pareto smoothing replaces the largest M weights with their corresponding rank-values from a fitted generalized Pareto-distribution [Zhang and Stephens, 2009]. Pareto smoothed importance sample (PSIS)-based LOO implementations are widely available in software packages such as Stan and ArviZ. However, PSIS-LOO is insufficient in problems where the Pareto distribution does not well-fit the tail distribution of importance weights – where the estimated Pareto shape parameter $\overset{ˆ}{k}$ exceeds 0.7 – this parameter is a useful criteria for accessing the validity of a given importance sampler. In such a case, it would be desirable to perform an additional model-specific controlled transformation on the proposal distribution that will induce more efficient computations.

### 2.5 ADAPTIVE IMPORTANCE SAMPLING

**Figure 1:**
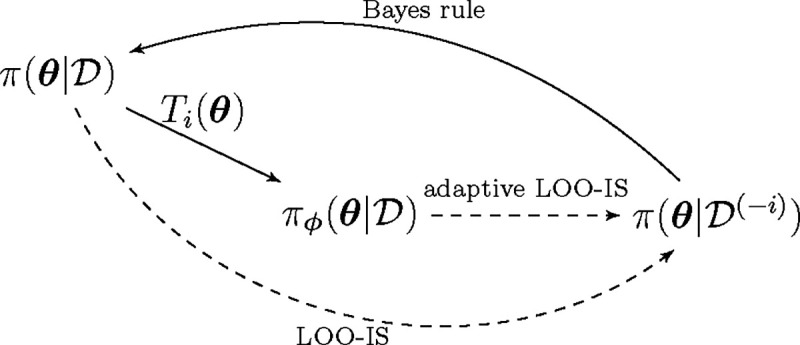
Relationships between probability densities. One wants to sample from $\pi (\theta |{𝒟}^{\left(-i\right)})$, the LOO distribution for observation i, by sampling from the full-data posterior π(θ∣𝒟). The transformation $T_{i}$ on the full-data posterior brings the sampling distribution closer to the target LOO distribution.

The objective of adaptive importance sampling [Bugallo et al., 2017, Cornuet et al., 2011, Elvira and Martino, 2022] in the context of LOO is to use a transformation to nudge, independently for each observation i, the posterior distribution closer to the LOO distribution $\pi (\theta |{𝒟}^{\left(-i\right)})$ (relationships between the different distributions is depicted in Fig. 1). First we’ll examine importance sampling under an arbitrary bijective transformation.

Consider the bijection $T_{i}\;:R^{p}\to R^{p}$, defined for observation i, and let $\phi \equiv T_{i}(\theta )$. By change of variables, $\pi_{\phi }(\phi |\ldots )=\pi \left(T_{i}^{-1}(\phi )|\ldots \right){𝒥}_{i}^{-1}(\phi )$, where we denote $J_{T}=\nabla T,\;{𝒥}_{T_{i}}^{-1}(\phi )=\left|J_{T_{i}}^{-1}(\phi )\right|$, and ${𝒥}_{T_{i}}(\theta )=\left|J_{T_{i}}(\theta )\right|=1/{𝒥}_{T_{i}}^{-1}(\phi )$, the determinants of the Jacobians of the inverse and forward transformations respectively. One can rewrite the expectation in Eq. 5 in terms of an integral over $\pi_{\phi }$,

(11) $$\begin{array}{l} E_{\theta |{𝒟}^{\left(-i\right)}}[f(\theta )]=\int \;f(\theta )\nu_{i}(\theta )\pi (\theta |𝒟)d\theta \\ \;=\int \;f(\theta )\nu_{i}(\theta )\frac{\pi (\theta |𝒟)}{\pi_{\phi }(\theta |𝒟)}\pi_{\phi }(\theta |𝒟)d\theta \\ \;=\int \;f\left(\theta \right)\nu_{i}\left(\theta \right)\frac{\pi \left(\theta |𝒟\right){𝒥}_{T_{i}}\left(T_{i}^{-1}\left(\theta \right)\right)}{\pi \left(T_{i}^{-1}\left(\theta \right)|𝒟\right)}\pi_{\phi }\left(\theta |𝒟\right)d\theta . \end{array}$$

One can then define a Monte-Carlo approximation of Eq. 11 using importance sampling, by sampling $\theta_{k}\overset{iid}{∼}\pi (\theta |𝒟)$ so that $\phi_{k}=T_{i}\left(\theta_{k}\right)\overset{iid}{∼}\pi_{\phi }(\phi |𝒟)$:

(12) $$E_{\theta |{𝒟}^{\left(-i\right)}}\left[f\left(\theta \right)\right]\approx \sum_{k=1}^{s}\frac{\eta_{ik}}{\sum_{j=1}^{s}\eta_{ij}}f\left(\phi_{k}\right)$$

(13) $$\eta_{ik}=\frac{{𝒥}_{T_{i}}\left(\theta_{k}\right)}{\ell \left(\phi_{k}|x_{i},\;y_{i}\right)}\frac{\pi \left(\phi_{k}|𝒟\right)}{\pi \left(\theta_{k}|𝒟\right)}.$$

By Bayes rule (Eq. 3), the posterior likelihood ratio in Eqs. 11–13 has the exact expression

(14) $$\frac{\pi \left(\phi |𝒟\right)}{\pi \left(\theta |𝒟\right)}=\prod_{i}\frac{\ell \left(\phi |x_{i},\;y_{i}\right)}{\ell \left(\theta |x_{i},\;y_{i}\right)}.$$

Computing this expression requires iterating over the entire dataset. For large datasets, one can turn to variational approximations.

### 2.6 ADAPTIVE IMPORTANCE SAMPLING USING VARIATIONAL POSTERIORS

For computational expediency, variational methods are often used in place of MCMC for Bayesian inference, obtaining a variational approximation $\overset{ˆ}{\pi }(\theta |𝒟)$ to the true posterior, where $\overset{ˆ}{\pi }$ lies within a given family of transformed probability distributions. In problems where one expects a substantial discrepancy between the true posterior and $\overset{ˆ}{\pi }$, one may correct for this discrepancy by noting that

(15) $$E_{\theta |{𝒟}^{\left(-i\right)}}[f(\theta )]=\int \;f(\theta )\nu_{i}(\theta )\pi (\theta |𝒟)d\theta \;\;=\int \;f(\theta )\nu_{i}(\theta )\frac{\pi (\theta |𝒟)}{{\overset{ˆ}{\pi }}_{\phi }(\theta |𝒟)}{\overset{ˆ}{\pi }}_{\phi }(\theta |𝒟)d\theta \;\;=\int \;f(\theta )\nu_{i}(\theta )\frac{\pi (\theta |𝒟){𝒥}_{T_{i}}\left(T_{i}^{-1}(\theta )\right)}{\overset{ˆ}{\pi }\left(T_{i}^{-1}(\theta )|𝒟\right)}{\overset{ˆ}{\pi }}_{\phi }(\theta |𝒟)d\theta$$

and using the self-normalized importance sampler

(16) $$E_{\theta |{𝒟}^{\left(-i\right)}}\left[f\left(\theta \right)\right]\approx \sum_{k=1}^{s}\frac{\chi_{ik}}{\sum_{j=1}^{s}\chi_{ij}}f\left(\phi_{k}\right)$$

(17) $$\chi_{ik}=\frac{{𝒥}_{i}\left(\theta_{k}\right)}{\hat{\pi }\left(\theta_{k}|𝒟\right)}\pi \left(\phi_{k}\right)\prod_{j\neq i}\ell \left(\phi_{k}|x_{j},y_{j}\right),$$

where $\pi \left(\phi_{k}\right)$ is the prior density at $\phi_{k}$, canceling out the two unknown constants corresponding to $\pi \left(\phi_{k}|𝒟\right)$ and $\nu_{i}$.

## 3 METHODS

Eq. 13 is valid for an arbitrary bijection $T_{i}$. The objective in using transformations is to shift the proposal distribution closer to the targeted LOO distribution for each observation – to partially invert Bayes rule. First, we motivate three different single-step transformations of the form

(18) $$T_{i}\left(\theta \right)=\theta +hQ_{i}\left(\theta \right),$$

for some small step-size h and function $Q_{i}$.

### 3.1 SINGLE STEP TRANSFORMATIONS

#### KL divergence descent:

We consider choosing $T_{i}$ to minimize the KL divergence $D_{KL}\left(\pi (\theta |{𝒟}^{\left(-i\right)})∥\pi_{\phi }(\theta |𝒟)\right)$, which is equivalent to minimizing the cross-entropy with respect to the mapping $T_{i}$,

(19) $$\begin{array}{l} H\left(\pi (\theta |{𝒟}^{\left(-i\right)}),\pi_{\phi }(\theta |𝒟)\right)=\\ \;-\int \;\nu_{i}(\phi )\pi (\phi |𝒟)log\frac{\pi \left(T_{i}^{-1}(\phi )|𝒟\right)}{{𝒥}_{T_{i}}\left(T_{i}^{-1}(\phi )\right)}d\phi . \end{array}$$

The Euler-Lagrange equation for minimizing Eq. 19 (derived in Supplemental Materials S.1.1), is implicit in $T_{i}$. While it admits no closed form solution, one may note that $T_{i}$ is a t→∞ stable fixed point of the KL-descending gradient flow

(20) $$\frac{\partial T_{i}(\theta ,\;t)}{\partial t}=-\frac{\delta H\left(\pi (\theta |{𝒟}^{\left(-i\right)}),\;\pi_{\phi }(\theta |𝒟)\right)}{\delta T_{i}}$$

and use this fact to refine, using the method of lines, an initial guess of $T_{i}(\theta )=\theta$ with forward Euler discretization of step-size $h{\left[E_{\theta |{𝒟}^{\left(-i\right)}}\left[\ell \left(\theta ,x_{i},y_{i}\right)\right]\right]}^{-1}$, for 0<h≪1, to arrive at the transformation

(21) $$\begin{array}{l} T_{i}^{KL}(\theta )=\theta -{\left.h\frac{\delta H\;\left(\pi (\theta |{𝒟}^{\left(-i\right)}),\;\pi_{\phi }(\theta |𝒟)\right)}{E_{\theta |{𝒟}^{\left(-i\right)}}[\ell (\theta ,\;x_{i},\;y_{i})]\delta T_{i}}\right|}_{T(\theta )=\theta }\\ \;=\theta +h\underset{Q_{i}^{KL}}{\underbrace{\pi (\theta |𝒟)\nabla \left(\frac{1}{\ell \left(\theta |x_{i},\;y_{i}\right)}\right)}}. \end{array}$$

#### Variance descent:

In importance sampling, the variance of the estimator is conditional on the target function for expectation. Since we are interested in computing the LOO predictive probability for each observation i, it is natural to consider minimizing the variance of the transformed importance sampler for the function $p_{i}(\theta )=p\left(\theta ,x_{i}\right)$. However, this objective yields a transformation that is only useful for observations where $y_{i}=0$ (see Supplemental MaterialsS.1.2). Instead, we seek to minimize the variance with respect to estimating the complement probability $p_{i}(\theta {)}^{1-y_{i}}{\left(1-p_{i}(\theta )\right)}^{y_{i}}$.

Starting from the associated variational problem (Appendix S.1.2), and applying the same rationale that went into developing the KL-descending transformation, one arrives at the single step variance-reducing transformation,

(22) $$\begin{array}{l} T_{i}^{Var}(\theta )=\theta +hQ_{i}^{Var}(\theta )\\ \;Q_{i}^{Var}(\theta )=\pi (\theta |𝒟){\left[\frac{1\;-\;p_{i}(\theta )}{p_{i}(\theta )}\right]}^{2y_{i}-1}\\ \;\times \nabla \left({\left[\frac{1\;-\;p_{i}(\theta )}{p_{i}(\theta )}\right]}^{2y_{i}-1}\right). \end{array}$$

#### Log-likelihood descent:

The two single step transformations of Eq. 21 require computing both the gradient and Hessian of the trained full-data posterior. We also consider a simpler alternate transformation [Elvira et al., 2023]

(23) $$T_{i}^{LL}(\theta )=\theta -h\underset{-Q_{i}^{LL}}{\underbrace{\nabla \;log\;\ell \left(\theta |y_{i},x_{i}\right)}},$$

equivalent to repelling each parameter sample a step h>0 *away* from the maximization of the log likelihood function for point i.

### 3.2 RESOLVING THE POSTERIOR DENSITY

Both the KL (Eq. 21) and variance (Eq. 22) descent transformations take steps proportional to the posterior density π(θ∣𝒟). If a variational approximation for π(θ∣𝒟) is available, using it in Eqs. 21 and 22 as a stand-in for the posterior density helps simplify the computation of the transformations and their Jacobians, particularly when using mean-field or low-order ADVI.

In the absence of variational approximation, one may evaluate the posterior densities exactly using Bayes rule, absorbing the unknown normalization constant 𝒵 into the step size h. Then, the KL and variance descent transformations are

(24) $$T_{i}^{\text{KL}}\left(\theta \right)=\theta +h\pi \left(\theta \right)\left[\prod_{j}\ell \left(\theta |x_{j},\;y_{j}\right)\right]\nabla \left(\frac{1}{\ell \left(\theta |x_{i},\;y_{i}\right)}\right),$$

and

(25) $$\begin{array}{l} T_{i}^{\text{Var}}\left(\theta \right)=\theta +hQ_{i}^{\text{Var}}\left(\theta \right)\\ Q_{i}^{\text{Var}}\left(\theta \right)=\pi \left(\theta \right)\left[\prod_{j}\ell \left(\theta |x_{j},y_{j}\right)\right]{\left[\frac{1\;-\;p_{i}\left(\theta \right)}{p_{i}\left(\theta \right)}\right]}^{2y_{i}-1}\\ \;\times \nabla \left({\left[\frac{1\;-\;p_{i}\left(\theta \right)}{p_{i}\left(\theta \right)}\right]}^{2y_{i}-1}\right). \end{array}$$

The obvious downside of using these exact transformations is the need to iterate over the entire dataset in order to evaluate the posterior density, which must be done for each parameter sample, for each data point.

For evaluating the Jacobian determinants, one appeals to Bayes rule to find that

(26) $$\nabla \log\left[𝒵\pi \left(\theta |𝒟\right)\right]=\nabla \log\pi \left(\theta \right)+\sum_{i}\nabla \;\text{log}\;\ell \left(\theta |x_{i},y_{i}\right),$$

where 𝒵 is is absorbed into h.

### 3.3 STEP SIZE SELECTION

The KL-divergence and variance descent transformations correspond to a forward Euler solver on the respective gradient flow equations. According to linear stability analysis, Euler’s method has the conditional stability criteria $h<2/{max}_{k}\;\left|Re\left(\lambda_{k}\right)\right|$ where $\lambda_{k}$ are the eigenvalues of the Jacobian of the system (Jacobians of the functions $Q_{i}$). In each case the structure of the Jacobian admits cheap approximations of $\lambda_{k}$. However, for nonlinear systems, this criterion is not sufficient for achieving stability.

Instead, we use a modified rule to determine the step size. For all parameter samples at each individual observation i, we use

(27) $$h_{i}=\rho \;\underset{s,\alpha }{min}\;\left\{\left|\frac{\sqrt{\Sigma_{\alpha ,\alpha }}}{Q_{i}{\left(\theta_{s}\right)}_{\alpha }}\right|\right\}$$

where ρ>0 and $\sqrt{\Sigma_{\alpha ,\alpha }}$ is the marginal posterior standard deviation of the α-th component of θ. This rule ensures that the transformation takes a step of at most ρ posterior standard deviations in any parameter component. The objective of adaptation is to find *any* transformation that results in importance weights where the Pareto tail shape is sub-threshold. To this end, one can compute the transformations for a range of ρ values in parallel using vectorized computations, saving computation at the cost of memory utilization.

### 3.4 JACOBIAN DETERMINANT APPROXIMATION

For any of the three single-step transformations, one may approximate $\left|J_{T_{i}}\right|$ by noting that

(28) $${𝒥}_{T_{i}}(\theta )=\left|1+h\nabla \cdot Q_{i}(\theta )\right|+𝒪\left(h^{2}\right)$$

and truncating to 𝒪(h), sidestepping the computation of Hessian matrices and their spectra. Note that any higher order terms in this expansion require characterization of the spectra of $\nabla Q_{i}$, for each observation i, and for each sampled parameter $\theta_{k}$. For large problems, computing the Jacobian matrix and its spectra this many times can become computationally problematic.

### 3.5 OVERVIEW

We have presented three single step transformations, each aimed at stabilizing a LOO importance sampler by bringing the proposal distribution closer to the LOO target in a different sense. The KL divergence and variance descent transformations correspond to the first-step of a forward Euler discretized solver for the corresponding gradient flow equations. The log-likelihood descent transformation repels the posterior parameter distribution away from the targeted LOO observation, undoing part of its contribution to the full data posterior. While each transformation uses gradient information, their Jacobians are simple to approximate, requiring no computation of full Hessian matrices.

Generally, one will find that many observations are amenable to direct importance sampling with 1/ℓ weights (Eq. 5) in combination with Pareto smoothing (tail weight distribution shape parameter $\overset{ˆ}{k}<0.7$). One needs only transform the sampling distribution when the estimated shape parameter exceeds this threshold. For a given posterior sample of model parameters $\theta_{1},\ldots ,\theta_{s}\overset{\text{iid}}{∼}\pi (\theta |𝒟)$, one undergoes the procedure for each given observation i:

**procedure AdaptiveIS**(observation i)

Compute weights $\nu_{ik}$ (Eq. 7) and their tail shape $\overset{ˆ}{k}$

**if** $\overset{ˆ}{k}\leq 0.7$ **then**

Done

**for** $T_{i}$ in transformations **do**

Apply $T_{i}$ to each $\theta_{k}$

Compute weights $\eta_{ik}$ (Eq. 13)

Compute $\overset{ˆ}{k}$

**if** $\overset{ˆ}{k}\leq 0.7$ **then**

Done

It is important to note that if *any* transformation takes $\overset{ˆ}{k}$ for a given observation under the threshold then adaptation is successful.

## 4 EXAMPLES

In this manuscript we consider the broad widely-used class of models that have a sigmoidal parameterization

(29) $$p_{i}(\theta )=p\left(\theta ,x_{i}\right)=\sigma \left(\mu_{i}(\theta )\right)$$

where $\sigma (\mu )=1/\left(1+e^{-\mu }\right)$ is the sigmoid function and we denote $\mu_{i}(\theta )\equiv \mu \left(\theta ,x_{i}\right)$ for some mean function μ.

For these models, the steps for each of the three transformations take the form

(30) $$Q_{i}^{KL}(\theta )=(-1{)}^{y_{i}}\pi (\theta |𝒟)e^{\mu_{i}(\theta )\left(1-2y_{i}\right)}\nabla \mu_{i}$$

(31) $$Q_{i}^{Var}(\theta )=(-1{)}^{y_{i}}\pi (\theta |𝒟)e^{2\mu_{i}(\theta )\left(1-2y_{i}\right)}\nabla \mu_{i}$$

(32) $$Q_{i}^{LL}\left(\theta \right)=-\left[y_{i}\left[1-\sigma \left(\mu_{i}\left(\theta \right)\right]-\left(1-y_{i}\right)\sigma \left(\mu_{i}\left(\theta \right)\right)\right]\nabla \mu_{i}\right.,$$

and their Jacobians take the form

(33) $$J_{T_{i}^{KL}}(\theta )=I+h(-1{)}^{y_{i}}\pi (\theta |𝒟)e^{\mu_{i}(\theta )\left(1-2y_{i}\right)}\{\nabla \nabla \mu_{i}\;\left.\;+\left[\nabla \;log\;\pi (\theta |𝒟)+\left(1-2y_{i}\right)\nabla \mu_{i}\right]{\left(\nabla \mu_{i}\right)}^{⊤}\right\}$$

(34) $$J_{T_{i}^{Var}}(\theta )=I+h(-1{)}^{y_{i}}\pi (\theta |𝒟)e^{2\mu_{i}(\theta )\left(1-2y_{i}\right)}\{\nabla \nabla \mu_{i}\;\left.\;+\left[\nabla \;log\;\pi (\theta |𝒟)+2\left(1-2y_{i}\right)\nabla \mu_{i}\right]{\left(\nabla \mu_{i}\right)}^{⊤}\right\}$$

(35) $$\begin{array}{r} J_{T_{i}^{LL}}(\theta )=I+h\left\{(1-\sigma \left(\mu_{i}(\theta )\right)\sigma \left(\mu_{i}(\theta )\right)\nabla \mu_{i}{\left(\nabla \mu_{i}\right)}^{⊤}\right.\\ \;-\left[y_{i}\left[1-\sigma \left(\mu_{i}(\theta )\right)\right]-\left(1-y_{i}\right)\sigma \left(\mu_{i}(\theta )\right)\right]\nabla \nabla \mu_{i}\}, \end{array}$$

where

(36) $$\begin{array}{l} \nabla \;\text{log}\;\pi \left(\theta |𝒟\right)=\nabla \;\text{log}\;\pi \left(\theta \right)\\ \;+\sum_{j}\left(y_{j}\left(1-\sigma \left(\mu_{j}\right)\right)-\left(1-y_{j}\right)\sigma \left(\mu_{j}\right)\right)\nabla \mu_{j}\left(\theta \right), \end{array}$$

and π(θ) is the prior.

Here we will consider two popular sub-families of sigmoidal models.

### 4.1 LOGISTIC REGRESSION (LR)

LR is a sigmoidal model where $\mu_{i}(\theta )=x_{i}^{⊤}\beta$, So, $\nabla_{\beta }\mu_{i}=x_{i}$, and ∇∇μ=0. Because the Hessian of μ vanishes, the Jacobian of the function $Q_{i}$ for each of the three functions is a rank-one matrix and has only a single non-zero eigenvalue. LR admits exact Jacobian determinants for each of the three transformations:

(37) $$\begin{array}{l} {𝒥}_{T_{i}^{KL}}(\theta )=\;|1+h(-1{)}^{y_{i}}\pi (\theta |𝒟)e^{\mu_{i}(\theta )\left(1-2y_{i}\right)}\\ \;\times \;x_{i}^{⊤}\left[\nabla \;log\;\pi (\theta |𝒟)+\left(1-2y_{i}\right)x_{i}\right]|, \end{array}$$

(38) $$\begin{array}{l} {𝒥}_{T_{i}^{Var}}(\theta )=|1+h(-1{)}^{y_{i}}\pi (\theta |𝒟)e^{2\mu_{i}(\theta )\left(1-2y_{i}\right)}\\ \;\times \;x_{i}^{⊤}\left[\nabla \;log\;\pi (\theta |𝒟)+2\left(1-2y_{i}\right)x_{i}\right]|, \end{array}$$

(39) $${𝒥}_{T_{i}^{LL}}\left(\theta \right)=|1+hx_{i}^{⊤}x_{i}\sigma \left(\mu_{i}\left(\theta \right)\right)\left(1-\sigma \left(\mu_{i}\left(\theta \right)\right)|\right..$$

### 4.2 BAYESIAN (RELU) NEURAL NETWORKS

Bayesian ReLU-nets [Lee, 2000, Ghosh and Doshi-Velez, 2017, Choi et al., 2018, Kristiadi et al., 2020, Bhadra et al., 2019] are piecewise linear [Sudjianto et al., 2021, Wang, 2022, Montúfar et al., 2014, Sudjianto et al., 2020] extensions to regression models. Being locally linear, these models have block-sparse Hessians and are also amenable to some limited degree of interpretability [Sudjianto et al., 2020, Chang et al., 2023]. One may write an L-layer ReLU Bayesian neural network recursively

(40) $$\begin{array}{c} y_{i}|\mu_{i}\;∼Bernoulli\left(\sigma \left(\mu_{i}\right)\right)\\ \mu_{i}|W_{L},b_{L},z_{L-1}^{\left(i\right)}\;=\mu \left(x_{i}\right)=W_{L}a(z_{L-1}^{\left(i\right)})+b_{L}\\ z_{k}|z_{k-1}^{\left(i\right)},b_{k},W_{k}\;=W_{k}a(z_{k-1}^{\left(i\right)})+b_{k}\\ z_{1}^{\left(i\right)}|W_{1},x_{i}\;=W_{1}x_{i}, \end{array}$$

where a is the ReLU activation function. The derivative of this function is the unit step function. We assume that the output function is sigmoid, noting that the softmax function also transforms into a sigmoid under a change of variables. Within the parameterization of Eq. 40 we absorbed the initial first-layer bias into the transformation $W_{1}$, by assuming that x has a unit constant component, as is the convention in regression.

The Hessian matrix of μ, while non-zero, is sparse because all of the following identities hold:

(41) $$\nabla_{b_{k}}\nabla_{b_{j}}\mu =0\;\;\;\;\;\;\forall j,k$$

(42) $$\nabla_{W_{k}}\nabla_{W_{k}}\mu =0\;\;\;\;\forall k$$

(43) $$\nabla_{w_{k}}\nabla_{b_{j}}\mu =0\;\;\;\;\;\;\forall j\geq k.$$

For this reason, the Jacobian determinant approximation of Eq. 28 can ignore the model Hessian entirely. However, in the case of one hidden layer we exploit the Hessian’s structure to provide explicit exact expressions for ${𝒥}_{\left(\cdot \right)}$.

**Example 4.1** (One hidden layer). These models are governed by the equations $\mu =W_{2}a\left(z_{1}\right)+b_{2}$ and $z_{1}=W_{1}x$, where $W_{2}\in R^{1\times d},\;b_{2}\in R,\;W_{1}\in R^{d\times p},\;b_{1}\in R^{d}$. This model has the first order derivatives $\partial_{{\left(W_{2}\right)}_{1i}}\mu =a{\left(z_{1}\right)}_{i},\;\partial_{{\left(W_{1}\right)}_{ij}}\mu ={\left(W_{2}\right)}_{1i}a^{\prime }\left({\left(z_{1}\right)}_{i}\right)x_{j},\;\partial_{b_{2}}\mu =1$. The only non-zero components of the Hessian matrix for μ are the mixed partial derivatives

(44) $$\frac{\partial^{2}\mu }{\partial {\left(W_{1}\right)}_{jk}\partial {\left(W_{2}\right)}_{1j}}=a^{\prime }({\left(z_{1}\right)}_{j})x_{k}.$$

The Hessian matrix of μ has a particular block structure that can be exploited (see Supplemental Materials S.2.1.1 for derivations) in order to find explicit expressions for its 2d non-zero eigenvalues, for k∈{1,2…,d},

(45) $$\lambda_{k}^{\pm }=\pm {\left[\sum_{j}a^{\prime }\left({\left(z_{1}\right)}_{k}\right)x_{j}^{2}\right]}^{1/2},$$

and associated eigenvectors

(46) $$v_{k}^{\pm }=\left(\begin{array}{c} {\tilde{u}}_{k}/\sqrt{2{\left|u_{k}\right|}^{2}}\\ \pm e_{k}/\sqrt{2}\\ 0 \end{array}\right),$$

where

(47) $${\tilde{u}}_{k}=(\overset{\left(k-1\right)p\;\text{zeros}}{\overbrace{0\;\ldots \;0}}u_{k}^{⊤}\;\overset{\left(d-k\right)p\;\text{zeros}}{\overbrace{0\ldots \ldots }}{)}^{⊤},$$

and $u_{k}=a^{\prime }\left({\left(z_{1}\right)}_{k}\right)x$. To compute the overall transformation Jacobians, one can then apply rank-one updates to ∇∇μ – a process that is aided by projecting the model gradients into the eigenspace of the model Hessian (see S.2.1 for derivations).

## 5 EXPERIMENTS

### 5.1 IMPLEMENTATION

We implemented our methodology in Python using the github:mederrata/bayesianquilts wrapper for Tensorflow probability [Dillon et al., 2017]. For Pareto smoothing, we used the Python implementation available at github:avehtari/PSIS.

### 5.2 DATASET AND MODEL

For demonstration, we used a public domain ovarian cancer micro-array dataset Hernández-Lobato et al. [2010], Schummer et al. [1999]. This dataset consists of n=54 observations of p=1056+1 predictors. As an example of a p≫n problem, model-agnostic 1/ℓ importance sampling is insufficient for computing LOO expectations. Notably, Paananen et al. [2021] used this dataset to test their moment-matching adaptive importance sample where they successfully decreased the number of observations where $\overset{ˆ}{k}>0.7$ from between 34 and 36 to between 11 and 20, depending on the number of posterior samples. We reproduced their logistic regression model, using the same regularized-horseshoe [Piironen and Vehtari, 2017b,c, Carvalho et al., 2009] prior, and the same statistical inference scheme using Stan, which we interfaced to Python using the package cmdstanpy. We ran four parallel Markov Chains, with twelve thousand burn-in iterations, retaining 2000 samples per chain after thinning to every other sample.

Prior to any adaptation, we estimated $\overset{ˆ}{k}$ for the corresponding 1/ℓ tail weights for this model. Repeatedly simulating 64 posterior draws, we found 27 ± 4 observations having $\overset{ˆ}{k}$ in excess of the stability cutoff.

### 5.3 DECREASING $\overset{ˆ}{k}$

**Figure 2:**
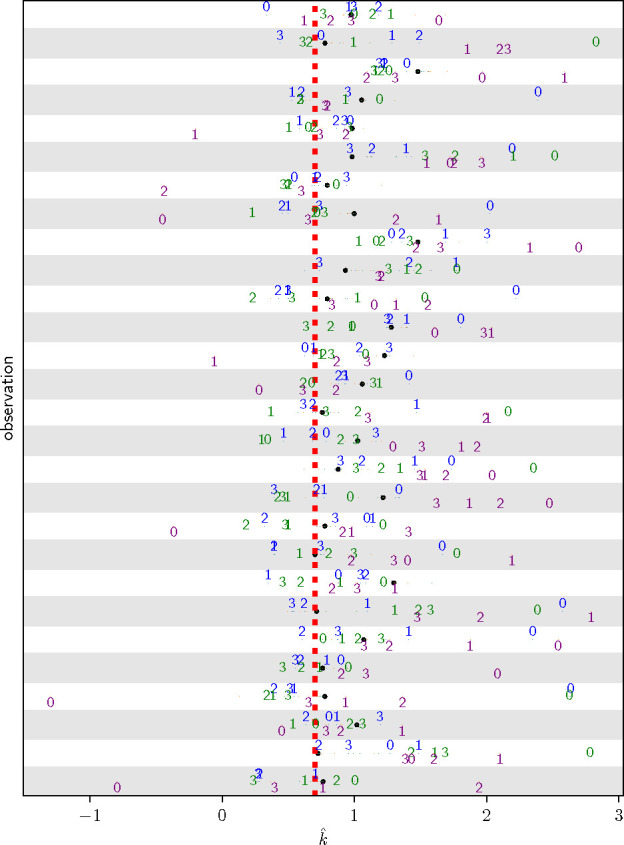
**Applying transformations (blue=KL descent, green=variance descent, purple=LL descent) for different values of** ρ (point labels are $-{log}_{10}\;\rho$) to posterior samples of a logistic regression model fitted to the ovarian cancer dataset and evaluating $\overset{ˆ}{k}$ for those observations where the untransformed 1/ℓ IS weights (Eq. 7) have $\overset{ˆ}{k}>0.7$.

We scanned different values of $\rho =10^{-r}$, for r∈{0,1,…,6}, evaluating all three transformations for a given value of ρ. We found that for 21 ± 4 of the initially $\overset{ˆ}{k}>0.7$ observations that at least one of the transformations reduced from super-threshold to sub-threshold in $\overset{ˆ}{k}$. Fig. 2 shows the results of one such simulated posterior draw. Generally, for too-large a value of ρ, all $\overset{ˆ}{k}$ values increased.

### 5.4 AREA UNDER ROC/PRC

Since n≪p, logistic regression is easily able to perfectly fit the dataset as defined by achieving in-training AUROC/AUPRC scores of approximately one.

**Figure 3:**
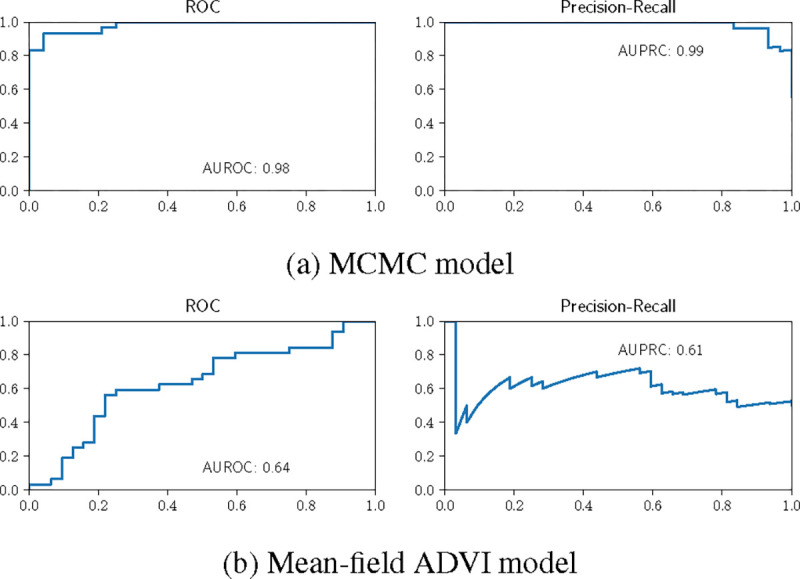
**LOO ROC curves for ovarian cancer classification model** contrasting the model fitted using MCMC and the model fitted using mean-field ADVI.

For the model trained using MCMC, the LOO AUROC and AUPRC curves have areas slightly less than one (Fig. 3a). In contrast, the model, when inferred using mean-field ADVI, has lower LOO AUROC and AUPRC scores. This finding is likely due to the fact that the MCMC model, when using the full posterior, captures associations between the model parameters – in effect finding a lower-dimensional “factorization” of the features. The mean-field model is unable to capture parameter correlations by design and its LOO performance suffers.

## 6 DISCUSSION

In this manuscript we introduced an adaptive importance sampler for using pre-trained full-data posteriors to approximate leave one out cross validation (LOO) in Bayesian classification models. The objective of adaptation to bring the sampling distribution (the full data posterior) closer to the target LOO posterior distributions for each data point. The methodology is based on taking a single step according to the gradient flow corresponding to minimization of a given objective. We introduce two such transformations: KL divergence descent and variance descent, along with a simple log-likelihood descent transformation. We presented explicit formulae for these transformations for logistic regression and ReLU-activated artificial neural networks with one hidden layer – the latter by computing the exact spectral decomposition of its Hessian matrix. We described how one can easily approximate the Jacobian of the transformations for more-complicated models, including for ReLU neural networks of any size. The adaptive importance sampler is ultimately used to estimate the expected LOO prediction for each given datapoint – quantities that can be used to compute downstream model generalization metrics such as ROC/PRC curves and the area under these curves.

### 6.1 LIMITATIONS

The main tradeoff of this method versus the model-agnostic PSIS-LOO method is that this method is model-dependent. In order to use this methodology for a given model, one needs to be able to evaluate gradients of the model with respect to parameters – and also the gradients of the corresponding prior distribution. Both the KL descent and variance descent transformations require computing the the posterior density – when a variational approximation of the posterior is not available or trustworthy this computation is costly for large datasets.

### 6.2 EXTENSIONS

In this manuscript we focused on classification problems but the methodology for adapting the importance sampler is much broader. In the Supplemental Materials one may find more-general formulae for the KL and variance descending transformations. In medical and industrial contexts, one is often interested in whether an individual or unit will experience an outcome within a certain time interval. For instance, policymakers are interested in hospital readmission within 30 days post discharge [Xia et al., 2023, Chang et al., 2023] because these readmissions are possibly preventable. In these problems, one may apply survival modeling to characterize the lifetime distribution, and additionally evaluate a model according to its classification performance at a given cut-off time T. Our methodology can easily be used for assessing such models.

Another extension to this methodology is to take more steps along the gradient flow for a given objective. Finally, we resolved the spectrum of the Hessian matrix for shallow ReLU models – the spectral decomposition for larger ReLU models may be useful for other analyses.

## Supplementary Material

Supplement 1

## References

[R1] Bhadra Anindya, Datta Jyotishka, Li Yunfan, and Polson Nicholas G.. Horseshoe Regularization for Machine Learning in Complex and Deep Models. April 2019.

[R2] Bugallo Monica F., Elvira Victor, Martino Luca, Luengo David, Miguez Joaquin, and Djuric Petar M.. Adaptive Importance Sampling: The past, the present, and the future. IEEE Signal Processing Magazine, 34(4):60–79, July 2017. ISSN 1558–0792. doi: 10.1109/MSP.2017. 2699226.

[R3] Carvalho Carlos M., Polson Nicholas G., and Scott James G.. Handling Sparsity via the Horseshoe. In Artificial Intelligence and Statistics, pages 73–80, April 2009.

[R4] Chang Joshua C., Chang Ted L., Chow Carson C., Mahajan Rohit, Mahajan Sonya, Maisog Joe, Vattikuti Shashaank, and Xia Hongjing. Interpretable (not just posthoc-explainable) medical claims modeling for discharge placement to prevent avoidable all-cause readmissions or death, January 2023. 10.1371/journal.pone.0302871 PMC11081343 38722929

[R5] Choi Arthur, Wang Ruocheng, and Darwiche Adnan. On the Relative Expressiveness of Bayesian and Neural Networks. December 2018.

[R6] Cornuet Jean-Marie, Marin Jean-Michel, Mira Antonietta, and Robert Christian P.. Adaptive Multiple Importance Sampling, October 2011.

[R7] Dillon Joshua V., Langmore Ian, Tran Dustin, Brevdo Eugene, Vasudevan Srinivas, Moore Dave, Patton Brian, Alemi Alex, Hoffman Matt, and Saurous Rif A.. Tensor-Flow Distributions. arXiv:1711.10604 [cs, stat], November 2017.

[R8] Elvira Víctor and Martino Luca. Advances in Importance Sampling, March 2022.

[R9] Elvira Víctor, Chouzenoux Émilie, Ömer Deniz Akyildiz, and Luca Martino. Gradient-based adaptive importance samplers. Journal of the Franklin Institute, 360(13):9490–9514, September 2023. ISSN 0016–0032. doi: 10.1016/j.jfranklin.2023.06.041.

[R10] Gelfand Alan E., Dey Dipak K., and Chang Hong. Model determination using predictive distributions with implementation via sampling-based methods. Bayesian statistics, 4:147–167, 1992.

[R11] Gelman Andrew, Hwang Jessica, and Vehtari Aki. Understanding predictive information criteria for Bayesian models. Statistics and Computing, 24(6):997–1016, November 2014. ISSN 1573–1375. doi: 10.1007/s11222-013-9416-2.

[R12] Ghosh Soumya and Doshi-Velez Finale. Model Selection in Bayesian Neural Networks via Horseshoe Priors. May 2017.

[R13] Hanley J. A. and McNeil B. J.. The meaning and use of the area under a receiver operating characteristic (ROC) curve. Radiology, 143(1):29–36, April 1982. ISSN 0033–8419. doi: 10.1148/radiology.143.1.7063747. 7063747

[R14] Hernández-Lobato Daniel, Hernández-Lobato José Miguel, and Suárez Alberto. Expectation Propagation for microarray data classification. Pattern Recognition Letters, 31 (12):1618–1626, September 2010. ISSN 0167–8655. doi: 10.1016/j.patrec.2010.05.007.

[R15] Ionides Edward L.. Truncated Importance Sampling. Journal of Computational and Graphical Statistics, 17(2):295–311, 2008. ISSN 1061–8600.

[R16] Kristiadi Agustinus, Hein Matthias, and Hennig Philipp. Being Bayesian, Even Just a Bit, Fixes Overconfidence in ReLU Networks, July 2020.

[R17] Lee H. K.. Consistency of posterior distributions for neural networks. Neural Networks: The Official Journal of the International Neural Network Society, 13(6):629–642, July 2000. ISSN 0893–6080. 10987516 10.1016/s0893-6080(00)00045-9

[R18] Guido Montúfar Razvan Pascanu, Cho Kyunghyun, and Bengio Yoshua. On the Number of Linear Regions of Deep Neural Networks, June 2014.

[R19] Paananen Topi, Piironen Juho, Paul-Christian Bürkner, and Aki Vehtari. Implicitly adaptive importance sampling. Statistics and Computing, 31(2):16, February 2021. ISSN 1573–1375. doi: 10.1007/s11222-020-09982-2.

[R20] Peruggia Mario. On the Variability of Case-Deletion Importance Sampling Weights in the Bayesian Linear Model. Journal of the American Statistical Association, 92(437):199–207, March 1997. ISSN 0162–1459. doi: 10.1080/01621459.1997.10473617.

[R21] Piironen Juho and Vehtari Aki. Comparison of Bayesian predictive methods for model selection. Statistics and Computing, 27(3):711–735, May 2017a. ISSN 1573–1375. doi: 10.1007/s11222-016-9649-y.

[R22] Piironen Juho and Vehtari Aki. On the Hyperprior Choice for the Global Shrinkage Parameter in the Horseshoe Prior. In AISTATS, 2017b.

[R23] Piironen Juho and Vehtari Aki. Sparsity information and regularization in the horseshoe and other shrinkage priors. Electronic Journal of Statistics, 11(2):5018–5051, 2017c. ISSN 1935–7524. doi: 10.1214/17-EJS1337SI.

[R24] Schummer Michèl, Ng WaiLap V, Bumgarner Roger E, Nelson Peter S, Schummer Bernhard, Bednarski David W, Hassell Laurie, Baldwin Rae Lynn, Karlan Beth Y, and Hood Leroy. Comparative hybridization of an array of 21 500 ovarian cDNAs for the discovery of genes overexpressed in ovarian carcinomas. Gene, 238(2):375–385, October 1999. ISSN 0378–1119. doi: 10.1016/S0378-1119(99)00342-X. 10570965

[R25] Stone M.. An Asymptotic Equivalence of Choice of Model by Cross-Validation and Akaike’s Criterion. Journal of the Royal Statistical Society. Series B (Methodological), 39(1):44–47, 1977. ISSN 0035–9246.

[R26] Sudjianto Agus, Knauth William, Singh Rahul, Yang Zebin, and Zhang Aijun. Unwrapping The Black Box of Deep ReLU Networks: Interpretability, Diagnostics, and Simplification. https://arxiv.org/abs/2011.04041v1, November 2020.

[R27] Sudjianto Agus, Qiu Jinwen, Li Miaoqi, and Chen Jie. Linear Iterative Feature Embedding: An Ensemble Framework for Interpretable Model. arXiv:2103.09983 [cs, stat], March 2021.

[R28] Vehtari Aki, Gelman Andrew, and Gabry Jonah. Pareto Smoothed Importance Sampling. arXiv:1507.02646 [stat], July 2015.

[R29] Vehtari Aki, Gelman Andrew, and Gabry Jonah. Practical Bayesian model evaluation using leave-one-out cross-validation and WAIC. Statistics and Computing, 27(5):1413–1432, September 2017. ISSN 1573–1375. doi: 10.1007/s11222-016-9696-4.

[R30] Wang Qing and Guo Alexandria. An efficient variance estimator of AUC and its applications to binary classification. Statistics in Medicine, 39(28):4281–4300, 2020. ISSN 1097–0258. doi: 10.1002/sim.8725. 32914457

[R31] Wang Yuan. Estimation and Comparison of Linear Regions for ReLU Networks. In Thirty-First International Joint Conference on Artificial Intelligence, volume 4, pages 3544–3550, July 2022. doi: 10.24963/ijcai.2022/492.

[R32] Watanabe Sumio. Asymptotic Equivalence of Bayes Cross Validation and Widely Applicable Information Criterion in Singular Learning Theory. Journal of Machine Learning Research, 11(Dec):3571–3594, 2010. ISSN ISSN 1533–7928.

[R33] Watanabe Sumio. A Widely Applicable Bayesian Information Criterion. Journal of Machine Learning Research, 14(Mar):867–897, 2013. ISSN ISSN 1533–7928.

[R34] Xia Hongjing, Chang Joshua C., Nowak Sarah, Mahajan Sonya, Mahajan Rohit, Chang Ted L., and Chow Carson C.. Interpretable (not just posthoc-explainable) heterogeneous survivor bias-corrected treatment effects for assignment of postdischarge interventions to prevent readmissions. https://arxiv.org/abs/2304.09981v1, April 2023.

[R35] Zhang Jin and Stephens Michael A.. A New and Efficient Estimation Method for the Generalized Pareto Distribution. Technometrics, 51(3):316–325, 2009. ISSN 0040–1706.

