# Supplementary material for: Gradient-flow adaptive importance sampling for Bayesian leave one out cross-validation with application to sigmoidal classification models
Source: ArXiv. 2024 Oct 20:arXiv:2402.08151v2. Originally published 2024 Feb 13. Preprint. [Version 2] (PMC11071546)
Supplement: Supplementary file 1 [file NIHPP2402.08151v2-supplement-1.pdf]

Gradient-flow adaptive importance sampling for Bayesian LOO (Supplementary Material)

October 22, 2024

S.1 Variational problems

S.1.1 KL Divergence

For convenience, we write the cross-entropy in the forward-transformation form, so that

$$\begin{aligned} H\left(\pi(\boldsymbol{\phi}|\mathcal{D}^{(-i)}), \pi_{\boldsymbol{\phi}}(\boldsymbol{\phi}|\mathcal{D})\right) &= -\int \nu_i(\boldsymbol{\phi})\pi(\boldsymbol{\phi}|\mathcal{D}) \log \frac{\pi(T_i^{-1}(\boldsymbol{\phi})|\mathcal{D})}{\mathcal{J}_i(T_i^{-1}(\boldsymbol{\phi}))} \mathrm{d}\boldsymbol{\phi} \\ &= -\int \nu_i(T_i(\boldsymbol{\theta}))\pi(T_i(\boldsymbol{\theta})|\mathcal{D}) \log \frac{\pi(\boldsymbol{\theta}|\mathcal{D})}{\mathcal{J}_i(\boldsymbol{\theta})} \mathcal{J}_{T_i}(\boldsymbol{\theta})\mathrm{d}\boldsymbol{\theta}. \end{aligned} \tag{S.1}$$

Now for an arbitrary test function with vanishing boundary conditions, ξ,

$$\begin{aligned} &\int \frac{\delta H\left(\pi(\boldsymbol{\theta}|\mathcal{D}^{(-i)}), \pi_{\boldsymbol{\phi}}(\boldsymbol{\theta}|\mathcal{D})\right)}{\delta T_i} \xi(\boldsymbol{\theta})\mathrm{d}\boldsymbol{\theta} \\ &\quad = -\lim_{\varepsilon\to 0} \frac{\mathrm{d}}{\mathrm{d}\varepsilon} \int \nu_i(T_i(\boldsymbol{\theta}) + \varepsilon\xi(\boldsymbol{\theta}))\pi(T_i(\boldsymbol{\theta}) + \varepsilon\xi(\boldsymbol{\theta})|\mathcal{D}) \log \frac{\pi(\boldsymbol{\theta}|\mathcal{D})}{|\nabla T_i(\boldsymbol{\theta}) + \varepsilon\nabla\xi(\boldsymbol{\theta})|} |\nabla T_i(\boldsymbol{\theta}) + \varepsilon\nabla\xi|\mathrm{d}\boldsymbol{\theta} \\ &\quad = -\int \nabla\left\{\pi(\boldsymbol{\theta}|\mathcal{D})\nu_i(\boldsymbol{\theta})\right\}\big|_{\boldsymbol{\theta}=T_i(\boldsymbol{\theta})} \mathcal{J}_i(\boldsymbol{\theta}) \log \frac{\pi(\boldsymbol{\theta}|\mathcal{D})}{\mathcal{J}_i(\boldsymbol{\theta})} \cdot \xi(\boldsymbol{\theta})\mathrm{d}\boldsymbol{\theta} \\ &\quad\quad - \int \nu_i(T_i(\boldsymbol{\theta}))\pi(T_i(\boldsymbol{\theta})|\mathcal{D}) \left[\mathcal{J}_{T_i}(\boldsymbol{\theta}) \log \frac{\pi(\boldsymbol{\theta}|\mathcal{D})}{\mathcal{J}_{T_i}(\boldsymbol{\theta})} - \mathcal{J}_{T_i}(\boldsymbol{\theta})\right] \mathrm{tr}\left(\mathbf{J}_i^{-1}(\boldsymbol{\theta})\nabla\xi(\boldsymbol{\theta})\right) \mathrm{d}\boldsymbol{\theta}. \end{aligned} \tag{S.2}$$

Re-arranging the trace term,

$$\mathrm{tr}\left[\mathbf{J}_i^{-1}\nabla\xi(\boldsymbol{\theta})\right] = \sum_q \sum_r (\mathbf{J}_i^{-1})_{qr}(\nabla\xi)_{rq} = \sum_r \sum_q \frac{\partial \xi_r}{\partial \theta_q} (\mathbf{J}_i^{-1}(\boldsymbol{\theta}))_{qr}, \tag{S.3}$$

and integrating each term by parts,

$$\begin{aligned} &\int \frac{\delta H\left(\pi(\boldsymbol{\theta}|\mathcal{D}^{(-i)}), \pi_{\boldsymbol{\phi}}(\boldsymbol{\theta}|\mathcal{D})\right)}{\delta T_i} \xi(\boldsymbol{\theta})\mathrm{d}\boldsymbol{\theta} \\ &\quad = -\int \nabla\left\{\pi(\boldsymbol{\theta}|\mathcal{D})\nu_i(\boldsymbol{\theta})\right\}\big|_{\boldsymbol{\theta}=T_i(\boldsymbol{\theta})} \mathcal{J}_i(\boldsymbol{\theta}) \log \frac{\pi(\boldsymbol{\theta}|\mathcal{D})}{\mathcal{J}_i(\boldsymbol{\theta})} \cdot \xi(\boldsymbol{\theta})\mathrm{d}\boldsymbol{\theta} \\ &\quad\quad + \sum_r \nabla\cdot \int \frac{\partial}{\partial\theta_q} \left\{\nu_i(T_i(\boldsymbol{\theta}))\pi(T_i(\boldsymbol{\theta})|\mathcal{D}) \left[\mathcal{J}_{T_i}(\boldsymbol{\theta}) \log \frac{\pi(\boldsymbol{\theta}|\mathcal{D})}{\mathcal{J}_{T_i}(\boldsymbol{\theta})} - \mathcal{J}_{T_i}(\boldsymbol{\theta})\right] (\mathbf{J}_i^{-1}(\boldsymbol{\theta}))_{qr}\right\} \mathbf{e}_r \cdot \xi\mathrm{d}\boldsymbol{\theta}. \end{aligned} \tag{S.4}$$

So, the Euler-Lagrange equation satisfies

$$\begin{aligned} \frac{\delta\mathcal{H}}{\delta T_i} &= -\nabla(\pi(\boldsymbol{\theta}|\mathcal{D})\nu_i(\boldsymbol{\theta}))\big|_{\boldsymbol{\theta}=T_i(\boldsymbol{\theta})} \mathcal{J}_i(\boldsymbol{\theta}) \log \frac{\pi(\boldsymbol{\theta}|\mathcal{D})}{\mathcal{J}_i(\boldsymbol{\theta})} \\ &\quad + \sum_r \nabla\cdot \left\{\nu_i(T_i(\boldsymbol{\theta}))\pi(T_i(\boldsymbol{\theta})|\mathcal{D}) \left[\mathcal{J}_{T_i}(\boldsymbol{\theta}) \log \frac{\pi(\boldsymbol{\theta}|\mathcal{D})}{\mathcal{J}_{T_i}(\boldsymbol{\theta})} - \mathcal{J}_{T_i}(\boldsymbol{\theta})\right] (\mathbf{J}_i^{-1}(\boldsymbol{\theta}))_{qr}\right\} \mathbf{e}_r \\ &= 0. \end{aligned} \tag{S.5}$$

We approximate T_i using the single-step (of size $0 < h \ll 1$) forward Euler update under the initial condition $T_i(\boldsymbol{\theta}, 0) = \boldsymbol{\theta}$,

$$T_i(\boldsymbol{\theta}) \approx T_i(\boldsymbol{\theta}, 0) - h \frac{\delta H\left(\pi(\boldsymbol{\theta}|\mathcal{D}^{(-i)}), \pi_{\boldsymbol{\phi}}(\boldsymbol{\theta}|\mathcal{D})\right)}{\delta T_i}\Big|_{T_i(\boldsymbol{\theta})=\boldsymbol{\theta}} = \boldsymbol{\theta} + h\pi(\boldsymbol{\theta}|\mathcal{D})\nabla\left(\frac{1}{\ell(\boldsymbol{\theta}|\mathbf{x}_i, y_i)}\right), \tag{S.6}$$

where we have absorbed the unknown normalizing constant into h.

S.1.2 Variance

The variance of the transformed importance sampling estimator of Eq. 11 is specific to the target function. We examine the variance in computing the expectation of a function $f(\boldsymbol{\theta})$ which is related to the variance of the individual element

$$\begin{aligned}
\mathrm{Var}\left[\nu_i(\boldsymbol{\phi})\mathcal{J}_{T_i}(T_i^{-1}(\boldsymbol{\phi}))\frac{\pi(\boldsymbol{\phi}|\mathcal{D})}{\pi(T_i^{-1}(\boldsymbol{\phi})|\mathcal{D})}f(\boldsymbol{\theta})\right] &= \int\left[\nu_i(\boldsymbol{\phi})\frac{\pi(\boldsymbol{\phi}|\mathcal{D})}{\pi_{\boldsymbol{\phi}}(\boldsymbol{\phi}|\mathcal{D})}f(\boldsymbol{\theta})\right]^2 \pi_{\boldsymbol{\phi}}(\boldsymbol{\phi}|\mathcal{D})\mathrm{d}\boldsymbol{\phi} \\
&\quad - \left\{\int\left[\nu_i(\boldsymbol{\phi})\frac{\pi(\boldsymbol{\phi}|\mathcal{D})}{\pi_{\boldsymbol{\phi}}(\boldsymbol{\phi}|\mathcal{D})}f(\boldsymbol{\theta})\right]\pi_{\boldsymbol{\phi}}(\boldsymbol{\phi}|\mathcal{D})\mathrm{d}\boldsymbol{\phi}\right\}^2 \\
&= \int \frac{\left[\nu_i(\boldsymbol{\phi})\pi(\boldsymbol{\phi}|\mathcal{D})f(\boldsymbol{\theta})\right]^2}{\pi_{\boldsymbol{\phi}}(\boldsymbol{\phi}|\mathcal{D})}\mathrm{d}\boldsymbol{\phi} + \text{constant relative to } T_i
\end{aligned} \tag{S.7}$$

Note that if one plugs in $f(\boldsymbol{\theta}) = p_i(\boldsymbol{\theta})$ into Eq. S.7, then when $y_i = 1$ the overall functional loses dependence on $\ell(\boldsymbol{\theta}|\mathbf{x}_i, y_i)$ because ν_i and $p_i(\boldsymbol{\theta})$ cancel. For this reason, in order to optimize with respect to the variance of the prediction, one can optimize against the symmetric function

$$f_i(\boldsymbol{\theta}) = p_i(\boldsymbol{\theta})^{1-y_i}(1 - p_i(\boldsymbol{\theta})^{y_i}.$$

Writing this expression in terms of the forward transformation and doing a change of variables $\mathrm{d}\boldsymbol{\phi} = \mathcal{J}_{T_i}(\boldsymbol{\theta})\mathrm{d}\boldsymbol{\theta}$, we write the functional to minimize

$$\begin{aligned}
\mathcal{V}[T_i] &= \int \frac{g(T_i(\boldsymbol{\theta}))}{\pi_{\boldsymbol{\phi}}(T_i(\boldsymbol{\theta})|\mathcal{D})}\mathcal{J}_{T_i}(\boldsymbol{\theta})\mathrm{d}\boldsymbol{\theta} \\
&= \int \frac{g(T_i(\boldsymbol{\theta}))}{\pi(\boldsymbol{\theta}|\mathcal{D})}\mathcal{J}_i^2(\boldsymbol{\theta})\mathrm{d}\boldsymbol{\theta}
\end{aligned} \tag{S.8}$$

$$g(\boldsymbol{\theta}) = (\nu_i(\boldsymbol{\theta})\pi(\boldsymbol{\theta}|\mathcal{D})f_i(\boldsymbol{\theta}))^2. \tag{S.9}$$

Computing the first variation of $\mathcal{V}$ with respect to T_i, using Gateaux differentiation,

$$\begin{aligned}
\delta\mathcal{V} &= \lim_{\varepsilon\to 0}\frac{\mathrm{d}}{\mathrm{d}\varepsilon}\int \frac{g(T_i(\boldsymbol{\theta}) + \varepsilon\xi)}{\pi(\boldsymbol{\theta}|\mathcal{D})}\left|\nabla T_i + \varepsilon\nabla\xi\right|^2 \mathrm{d}\boldsymbol{\theta} \\
&= \int\left\{\frac{\nabla g(T_i(\boldsymbol{\theta}))\mathcal{J}_i^2(\boldsymbol{\theta})}{\pi(\boldsymbol{\theta}|\mathcal{D})}\cdot\xi(\boldsymbol{\theta}) + 2\frac{g(T_i(\boldsymbol{\theta}))\mathcal{J}_i^2(\boldsymbol{\theta})}{\pi(\boldsymbol{\theta}|\mathcal{D})}\mathrm{tr}\left(\mathbf{J}_i^{-1}(\boldsymbol{\theta})\nabla\xi(\boldsymbol{\theta})\right)\right\}\mathrm{d}\boldsymbol{\theta} \\
&= \int\left\{\frac{\nabla g(T_i(\boldsymbol{\theta}))\mathcal{J}_i^2(\boldsymbol{\theta})}{\pi(\boldsymbol{\theta}|\mathcal{D})} - 2\sum_r \nabla\cdot\left[\frac{g(T_i(\boldsymbol{\theta}))\mathcal{J}_i^2(\boldsymbol{\theta})}{\pi(\boldsymbol{\theta}|\mathcal{D})}\mathbf{J}_i^{-1}(\boldsymbol{\theta})_{,r}\right]\mathbf{e}_r\right\}\cdot\xi(\boldsymbol{\theta})\mathrm{d}\boldsymbol{\theta}.
\end{aligned} \tag{S.10}$$

So the Euler-Lagrange equation for minimizing the variance is

$$\frac{\delta\mathcal{V}}{\delta T_i} = \frac{\nabla g(T_i(\boldsymbol{\theta}))\mathcal{J}_i^2(\boldsymbol{\theta})}{\pi(\boldsymbol{\theta}|\mathcal{D})} - 2\sum_r \nabla\cdot\left[\frac{g(T_i(\boldsymbol{\theta}))\mathcal{J}_i^2(\boldsymbol{\theta})}{\pi(\boldsymbol{\theta}|\mathcal{D})}\mathbf{J}_i^{-1}(\boldsymbol{\theta})_{,r}\right]\mathbf{e}_r = 0. \tag{S.11}$$

For $T_i(\boldsymbol{\theta}) = \boldsymbol{\theta}$, the functional derivative is

$$\begin{aligned}
\frac{\delta \mathcal{V}}{\delta T_i}\Big|_{T_i=\boldsymbol{\theta}} &= \frac{\nabla g(\boldsymbol{\theta})}{\pi(\boldsymbol{\theta}|\mathcal{D})} - 2\nabla\left(\frac{g(\boldsymbol{\theta})}{\pi(\boldsymbol{\theta}|\mathcal{D})}\right) \\
&= \frac{\nabla g(\boldsymbol{\theta})}{\pi(\boldsymbol{\theta}|\mathcal{D})} - 2\frac{\nabla g(\boldsymbol{\theta})}{\pi(\boldsymbol{\theta}|\mathcal{D})} + 2\frac{g\nabla\pi(\boldsymbol{\theta})}{\pi^2(\boldsymbol{\theta}|\mathcal{D})} \\
&= -\frac{2\nu_i(\boldsymbol{\theta})\pi(\boldsymbol{\theta}|\mathcal{D})f_i(\boldsymbol{\theta})\nabla(\nu_i(\boldsymbol{\theta})\pi(\boldsymbol{\theta})f_i(\boldsymbol{\theta}))}{\pi(\boldsymbol{\theta}|\mathcal{D})} + 2\frac{g\nabla\pi(\boldsymbol{\theta})}{\pi^2(\boldsymbol{\theta}|\mathcal{D})} \\
&= -2\nu_i(\boldsymbol{\theta})f_i(\boldsymbol{\theta})\nabla(\nu_i(\boldsymbol{\theta})\pi(\boldsymbol{\theta}|\mathcal{D})f_i(\boldsymbol{\theta})) + 2\nu_i^2(\boldsymbol{\theta})f_i^2(\boldsymbol{\theta})\nabla\pi(\boldsymbol{\theta}|\mathcal{D}) \\
&= -2\nu_i(\boldsymbol{\theta})f_i(\boldsymbol{\theta})\pi(\boldsymbol{\theta}|\mathcal{D})\nabla\big[\nu_i(\boldsymbol{\theta})f_i(\boldsymbol{\theta})\big] \qquad \text{(S.12)}
\end{aligned}$$

S.2 Sigmoidal models

For these models one may use the chain rule to write the gradient,

$$\nabla\ell(\boldsymbol{\theta}|\mathbf{x},y) = \ell(\boldsymbol{\theta}|\mathbf{x},y)\nabla\log\ell(\boldsymbol{\theta}|\mathbf{x},y) \qquad \text{(S.13)}$$

$$\nabla\log\ell(\boldsymbol{\theta}|\mathbf{x},y) = \underbrace{[y(1-\sigma(\mu)) - (1-y)\sigma(\mu)]}_{(\log\ell)'}\nabla\mu \qquad \text{(S.14)}$$

and Hessian

$$\begin{aligned}
\nabla\nabla\ell(\boldsymbol{\theta}|\mathbf{x},y) &= \ell(\boldsymbol{\theta}|\mathbf{x},y)\nabla\nabla\log\ell(\boldsymbol{\theta}|\mathbf{x},y) \\
&\quad + \ell(\boldsymbol{\theta}|\mathbf{x},y)\nabla\log\ell(\boldsymbol{\theta}|\mathbf{x},y)\nabla\log\ell(\boldsymbol{\theta}|\mathbf{x},y) \qquad \text{(S.15)} \\
\nabla\nabla\log\ell(\boldsymbol{\theta}|\mathbf{x},y) &= [y(1-\sigma(\mu)) - (1-y)\sigma(\mu)]\nabla\nabla\mu \\
&\quad \underbrace{-\sigma(\mu)(1-\sigma(\mu))}_{(\log\ell)''}\nabla\mu\nabla\mu \qquad \text{(S.16)}
\end{aligned}$$

for the likelihood function as a function of the gradient and Hessian of μ.

For the sake of space, we drop all indices i and let $\pi = \pi(\boldsymbol{\theta}|\mathbf{x})$ and $\ell = \ell(\boldsymbol{\theta}|\mathbf{x},y)$ All transformations are of the form $T(\boldsymbol{\theta}) = \boldsymbol{\theta} + Q(\boldsymbol{\theta})$

S.2.1 ReLU with one hidden layer

Ordering the parameters $(W_1)_{11},\ldots,(W_1)_{1p},(W_1)_{21},\ldots,(W_1)_{2p},(W_1)_{d1},\ldots,(W_1)_{dp},(W_1)_1,(W_2)_1,\ldots,(W_2)_d,b_2$, the Hessian of μ takes the form

$$\nabla\nabla^{\mathsf{T}}\mu = \begin{pmatrix} \mathbf{0} & \mathbf{M} & \mathbf{0} \\ \mathbf{M}^{\mathsf{T}} & \mathbf{0} & \mathbf{0} \\ \mathbf{0} & \mathbf{0} & 0 \end{pmatrix}, \qquad \text{(S.17)}$$

where $\mathbf{M}$ encodes the mixed partial derivatives of μ with respect to the elements of $\mathbf{W}_1$ and $\mathbf{W}_2$,

$$\frac{\partial^2\mu}{\partial(W_1)_{ij}(W_2)_{1k}} = \delta_{ik}a'((z_1)_i)x_j. \qquad \text{(S.18)}$$

The $dp \times d$ matrix $\mathbf{M}$ takes the form

$$\mathbf{M} = \begin{pmatrix} \mathbf{u}_1 & \mathbf{0} & \mathbf{0} & \cdots & \mathbf{0} \\ \mathbf{0} & \mathbf{u}_2 & \mathbf{0} & \cdots & \mathbf{0} \\ \mathbf{0} & \mathbf{0} & \mathbf{u}_3 & \ddots & \vdots \\ \vdots & \vdots & \ddots & \ddots & \vdots \\ \mathbf{0} & \cdots & \cdots & \cdots & \mathbf{u}_d \end{pmatrix} \qquad \text{(S.19)}$$

where each column vector

$$\mathbf{u}_k = \begin{pmatrix} a'((z_1)_k)x_1 & a'((z_1)_k)x_2 & \cdots & a'((z_1)_k)x_p \end{pmatrix}^{\mathsf{T}} = a'((z_1)_k)\mathbf{x} \qquad \text{(S.20)}$$

is potentially sparse.

S.2.1.1 Hessian decomposition

Suppose that λ is an eigenvalue of $\nabla\nabla^\intercal\mu$, corresponding to the eigenvector $\boldsymbol{\psi} = \begin{pmatrix}\boldsymbol{\psi}_1^\intercal & \boldsymbol{\psi}_2^\intercal & \boldsymbol{\psi}_3^\intercal\end{pmatrix}^\intercal$. Then,

$$\mathbf{M}\boldsymbol{\psi}_2 = \lambda\boldsymbol{\psi}_1 \tag{S.21}$$
$$\mathbf{M}^\intercal\boldsymbol{\psi}_1 = \lambda\boldsymbol{\psi}_2 \tag{S.22}$$
$$\boldsymbol{\psi}_3 = \mathbf{0}. \tag{S.23}$$

Left-multiplying Eq. S.21 by $\mathbf{M}^\intercal$ and Eq. S.22 by $\mathbf{M}$, one finds that

$$\mathbf{M}^\intercal\mathbf{M}\boldsymbol{\psi}_2 = \lambda\mathbf{M}^\intercal\psi_1 = \lambda^2\boldsymbol{\psi}_2 \tag{S.24}$$
$$\mathbf{M}\mathbf{M}^\intercal\boldsymbol{\psi}_1 = \lambda\mathbf{M}\mathbf{M}^\intercal\boldsymbol{\psi}_2 = \lambda^2\boldsymbol{\psi}_1. \tag{S.25}$$

Eq. S.24 implies that solutions for $\boldsymbol{\psi}_2$ are eigenvectors of $\mathbf{M}^\intercal\mathbf{M}$ and the solutions for $\boldsymbol{\psi}_1$ are eigenvectors of $\mathbf{M}\mathbf{M}^\intercal$.

The matrix $\mathbf{M}^\intercal\mathbf{M}$ is the diagonal matrix $\text{diag}(|\mathbf{u}_k|^2)$, so the non-zero eigenvalues are each of $\pm|u_k|$, and the solutions for $\boldsymbol{\psi}_2$ are proportional to elements of the d dimensional standard basis.

It is easy to see that the eigenvectors of $\mathbf{M}\mathbf{M}^T$, which we denote $\tilde{\mathbf{u}}_k$, are related to the column vectors of $\mathbf{M}$,

$$\tilde{\mathbf{u}}_k = (\overbrace{0 \quad \ldots \quad 0}^{(k-1)p \text{ zeros}} \quad \mathbf{u}_k^\intercal \quad \overbrace{0 \quad \ldots \quad 0}^{(d-k)p \text{ zeros}})^\intercal. \tag{S.26}$$

Overall, each eigenvector for $\nabla\nabla^\intercal\mu$ takes the form $\begin{pmatrix}\tilde{\mathbf{u}}_k^\intercal & \gamma\mathbf{e}_k^\intercal & 0\end{pmatrix}^\intercal$ for some constant γ. By Eq. S.24,

$$\mathbf{M}\gamma\mathbf{e}_k = \gamma\tilde{\mathbf{u}}_k = \lambda\tilde{\mathbf{u}}_k,$$

so $\gamma = \lambda$. Hence, by spectral theorem, we may write the decomposition for $\nabla\nabla^\intercal\mu$,

$$\nabla\nabla^\intercal\mu = \sum_{k=1}^{d}\left[|\mathbf{u}_k|\underbrace{\begin{pmatrix}\tilde{\mathbf{u}}_k/\sqrt{2|\mathbf{u}_k|^2}\\ \mathbf{e}_k/\sqrt{2}\\ 0\end{pmatrix}}_{\mathbf{v}_{k+}}\begin{pmatrix}\tilde{\mathbf{u}}_k^\intercal/\sqrt{2|\mathbf{u}_k|^2} & \mathbf{e}_k^\intercal/\sqrt{2} & 0\end{pmatrix} - |\mathbf{u}_k|\underbrace{\begin{pmatrix}\tilde{\mathbf{u}}_k/\sqrt{2|\mathbf{u}_k|^2}\\ -\mathbf{e}_k/\sqrt{2}\\ 0\end{pmatrix}}_{\mathbf{v}_{k-}}\begin{pmatrix}\tilde{\mathbf{u}}_k^\intercal/\sqrt{2|\mathbf{u}_k|^2} & -\mathbf{e}_k^\intercal/\sqrt{2} & 0\end{pmatrix}\right]. \tag{S.27}$$

S.2.1.2 Overall model Jacobians

To assemble the Jacobian of the overall transformation, we need to diagonalize the gradient outer product simultaneously with $\mathbf{M}$. The gradient terms follow

$$\frac{\partial\mu}{\partial b_2} = 1 \tag{S.28}$$
$$\frac{\partial\mu}{\partial(W_2)_{1k}} = a((z_1)_k) \tag{S.29}$$
$$\frac{\partial\mu}{\partial(W_1)_{jk}} = (W_2)_j a'((z_1)_j)x_k \tag{S.30}$$

so that

$$\nabla\mu = \begin{pmatrix}(W_2)_1\mathbf{u}_1^\intercal & (W_2)_2\mathbf{u}_2^\intercal & \cdots & (W_2)_d\mathbf{u}_d^\intercal & a((z_1)_1) & a((z_1)_2) & \cdots & a((z_1)_d) & 1\end{pmatrix}^\intercal \tag{S.31}$$

We then decompose the gradient into the eigenspace of the Hessian,

$$\begin{aligned}\nabla\mu &= \sum_{k=1}^{d}\left[\frac{(W_2)_k|\mathbf{u}_k| + a((z_1)_k)}{\sqrt{2}}\mathbf{v}_{k+} + \frac{(W_2)_k|\mathbf{u}_k| - a((z_1)_k)}{\sqrt{2}}\mathbf{v}_{k-}\right] + \underbrace{\begin{pmatrix}0 & \cdots & 0 & 1\end{pmatrix}^\intercal}_{\mathbf{e}_{dp+d+1}}\\ &= \mathbf{V}\mathbf{c}\end{aligned} \tag{S.32}$$

where $\mathbf{V}$ is the matrix where the columns are the eigenvectors

$$\mathbf{V} = \begin{pmatrix}\mathbf{v}_{1+} & \mathbf{v}_{1-} & \mathbf{v}_{2+} & \mathbf{v}_{2-} & \cdots & \mathbf{v}_{d+} & \mathbf{v}_{d-} & \mathbf{e}_{dp+d+1}\end{pmatrix}, \tag{S.33}$$

and $\mathbf{c}$ is a column vector corresponding to the coefficients in Eq. S.32.

Putting it all together, the Jacobians of the transformation are, for the log-likelihood descent tranformation,

$$\begin{aligned}\mathbf{I} - h\nabla\nabla^{\intercal}\log\ell = \mathbf{I} - h\Bigg\{&[y(1-\sigma(\mu)) - (1-y)\sigma(\mu)]\overbrace{\left[\sum_{k=1}^{d}\left(|\mathbf{u}_k|\mathbf{v}_{k+}\mathbf{v}_{k+}^{\intercal} - |\mathbf{u}_k|\mathbf{v}_{k-}\mathbf{v}_{k-}^{\intercal}\right)\right]}^{\nabla\nabla^{\intercal}\mu}\\ &- \sigma(\mu)(1-\sigma(\mu))\mathbf{V}\mathbf{c}\mathbf{c}^{\intercal}\mathbf{V}^{\intercal}\Bigg\}.\end{aligned} \tag{S.34}$$

To compute the determinant of Eq. S.34 we first make two observations. First, two similar matrices have the same determinant, so that

$$\begin{aligned}\det\left[\mathbf{I} - h\nabla\nabla^{\intercal}\log\ell\right] &= \det\left[\mathbf{I} - h\left[[y(1-\sigma(\mu)) - (1-y)\sigma(\mu)]\mathbf{V}\mathrm{diag}(\lambda_i)\mathbf{V}^{\intercal} - \sigma(\mu)(1-\sigma(\mu))\mathbf{V}\mathbf{c}\mathbf{c}^{\intercal}\mathbf{V}^{\intercal}\right]\right]\\ &= \det\left[\underbrace{\mathbf{I} - h\left[[y(1-\sigma(\mu)) - (1-y)\sigma(\mu)]\mathrm{diag}(\lambda_i)\right]}_{\text{diagonal terms from } \mathbf{I} \text{ and } \nabla\nabla^{\intercal}\mu} + h \underbrace{\sigma(\mu)(1-\sigma(\mu))\mathbf{c}\mathbf{c}^{\intercal}}_{\text{from gradient outer product term}}\right],\end{aligned} \tag{S.35}$$

and second that the non-diagonal elements of the matrix in Eq. S.35 are rank-one so that

$$\begin{aligned}\mathcal{J}_{T^{\mathrm{LL}}}(\boldsymbol{\theta}) = &\prod_{j=1}^{d}\left(1 - h^2[y(1-\sigma(\mu)) - (1-y)\sigma(\mu)]^2|\mathbf{u}_j|^2\right)\\ &\times\left(1 + h\sigma(\mu)(1-\sigma(\mu))\left[\sum_{k=1}^{d}\left[\frac{[(W_2)_k|\mathbf{u}_k| + a((z_1)_k)]^2}{2[1 - h[y(1-\sigma(\mu)) - (1-y)\sigma(\mu)]|\mathbf{u}_k|]}\right.\right.\right.\\ &\left.\left.\left.+\frac{[(W_2)_k|\mathbf{u}_k| - a((z_1)_k)]^2}{2[1 + h[y(1-\sigma(\mu)) - (1-y)\sigma(\mu)]|\mathbf{u}_k|]}\right] + 1\right]\right).\end{aligned} \tag{S.36}$$

S.3 Modified Moment Matching method

The first two transformation methods presented in [Paananen et al., 2021] match the first moment and the first two moments respectively of the proposal distribution and the target distribution, independently for each scalar component of each model parameter. We generalize those distributions subject to a tunable scalar constant ρ,

$$T_1(\boldsymbol{\theta}) = \boldsymbol{\theta} + \rho(\bar{\boldsymbol{\theta}}_w - \bar{\boldsymbol{\theta}}) \tag{S.37}$$

$$T_2(\boldsymbol{\theta}) = \boldsymbol{\theta} + \rho\left(\mathbf{v}_w^{1/2} \circ \mathbf{v}^{-1/2} \circ (\boldsymbol{\theta} - \bar{\boldsymbol{\theta}}) + \bar{\boldsymbol{\theta}}_w - \boldsymbol{\theta}\right) \tag{S.38}$$

$$\bar{\boldsymbol{\theta}} = \frac{1}{s}\sum_{k=1}^{s}\boldsymbol{\theta}_k \tag{S.39}$$

$$\bar{\boldsymbol{\theta}}_w = \frac{\sum_{k=1}^{s}\nu_k\boldsymbol{\theta}_k}{\sum_k \nu_k}$$

$$\mathbf{v} = \frac{1}{s}\sum_{k=1}^{s}(\boldsymbol{\theta}_k - \bar{\boldsymbol{\theta}}) \circ (\boldsymbol{\theta}_k - \bar{\boldsymbol{\theta}}) \tag{S.40}$$

$$\mathbf{v}_w = \frac{\sum_{k=1}^{s}\nu_k(\boldsymbol{\theta}_k - \bar{\boldsymbol{\theta}}) \circ (\boldsymbol{\theta}_k - \bar{\boldsymbol{\theta}})}{\sum_{k=1}^{s}\nu_k} \tag{S.41}$$

where $\rho = 1$ recovers the original transformations.

S.4 Supplemental Results

S.4.1 Bayesian logistic regression

In Fig. S.1 we display the posterior expectation for the regresion coefficients. Fig. S.2, provides a more-detailed version of Fig. 2 in the main text, where each point is labeled by its corresponding vable of $\log_4(\rho)$.

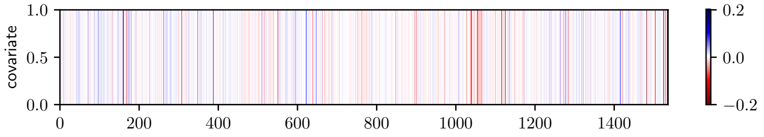

Figure S.1: **Ovarian cancer logistic regression coefficients** for MCMC-based fitting

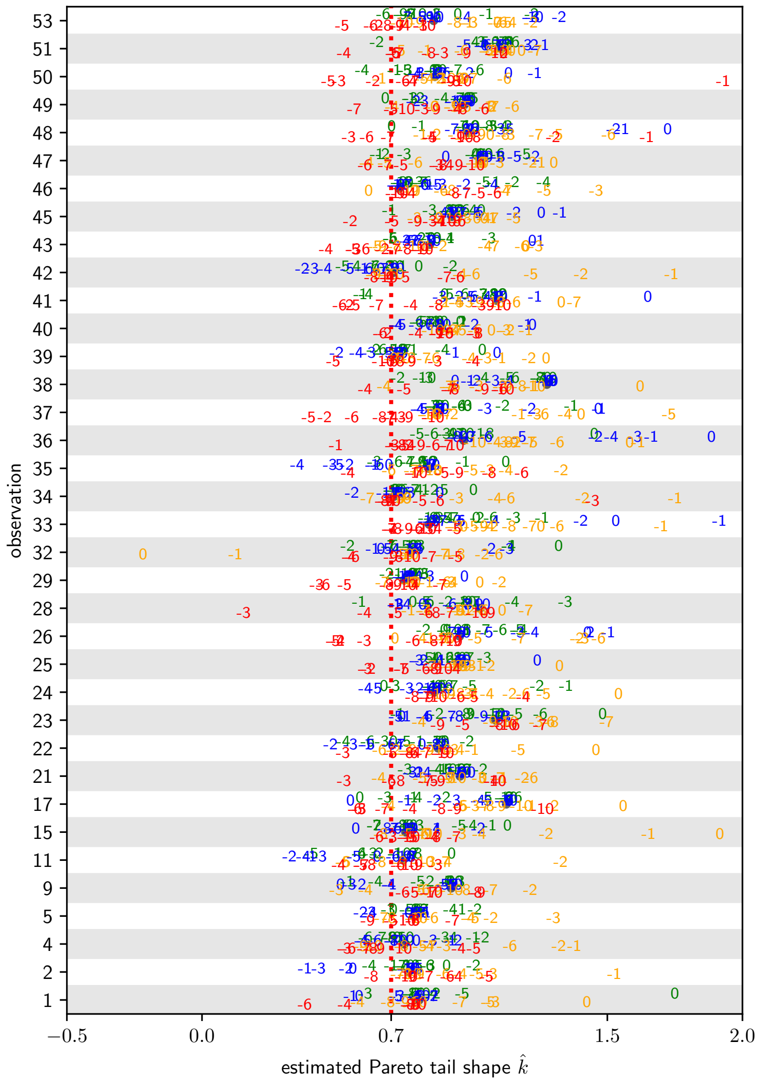

Figure S.2: **Estimated Pareto tail shape diagnostic** $\hat{k}$, for logistic regression on the ovarian cancer dataset, for observations where $\hat{k} > 0.7$ (shown as dots •). Post-transformed values of $\hat{k}$ for KL descent (green) and Variance descent (blue), LL descent (orange), MM1 (red) plotted, with location of the minimum observed value for each transformation labeled. Adaptation is successful if $\hat{k} < 0.7$.

S.4.2 Bayesian ReLU-net

We also implemented our methods for shallow Bayesian ReLU-nets. In section S.6, we re-analyze the same ovarian microarray dataset using a ReLU-activated Bayesian neural network with a hidden layer of size three.

S.5 Jupyter notebooks for logistic regression

In the following pages, please find Jupyter notebooks that we used for producing the results in the main text.

In [1]:
```
%matplotlib inline
from itertools import chain, combinations
import matplotlib
from matplotlib import pyplot as plt
matplotlib.rcParams['pdf.fonttype'] = 42
matplotlib.rcParams['ps.fonttype'] = 42
matplotlib.rcParams['text.usetex'] = True
from abc import ABC
from cmdstanpy import CmdStanModel
import numpy as npe
import pandas as pd
from pathlib import Path
from tqdm import tqdm
import nest_asyncio
nest_asyncio.apply()
from collections import defaultdict
from typing import Any

from sklearn import metrics
from matplotlib import pyplot as plt
```

In [2]:
```
import tensorflow as tf
import tensorflow_probability as tfp
import tensorflow_probability.python.bijectors as tfb

# pip install github:https://github.com/mederrata/bayesianquilts
from bayesianquilts.models.logistic_regression_reparam import LogisticRegression2
from bayesianquilts.metrics.classification import classification_metrics, auroc, auprc
from bayesianquilts.sampler import psis, nppsis
```

```
2024-10-20 08:42:16.913902: I external/local_tsl/tsl/cuda/cudart_stub.cc:31] Could not find cuda drivers on your machine, GPU will not be used.
2024-10-20 08:42:16.937684: E external/local_xla/xla/stream_executor/cuda/cuda_dnn.cc:9261] Unable to register cuDNN factory: Attempting to register factory for plugin cuDNN when one has alread
y been registered
2024-10-20 08:42:16.937710: E external/local_xla/xla/stream_executor/cuda/cuda_fft.cc:607] Unable to register cuFFT factory: Attempting to register factory for plugin cuFFT when one has already
been registered
2024-10-20 08:42:16.938552: E external/local_xla/xla/stream_executor/cuda/cuda_blas.cc:1515] Unable to register cuBLAS factory: Attempting to register factory for plugin cuBLAS when one has alr
eady been registered
2024-10-20 08:42:16.942787: I external/local_tsl/tsl/cuda/cudart_stub.cc:31] Could not find cuda drivers on your machine, GPU will not be used.
2024-10-20 08:42:16.943613: I tensorflow/core/platform/cpu_feature_guard.cc:182] This TensorFlow binary is optimized to use available CPU instructions in performance-critical operations.
To enable the following instructions: AVX2 FMA, in other operations, rebuild TensorFlow with the appropriate compiler flags.
2024-10-20 08:42:17.630884: W tensorflow/compiler/tf2tensorrt/utils/py_utils.cc:38] TF-TRT Warning: Could not find TensorRT
```

In [ ]:
```
X_ = pd.read_csv(Path.home() / "workspace" / "bayesianquilts"/ "bayesianquilts"/ "data" / "overianx.csv", header=None)
y_ = pd.read_table(Path.home() / "workspace" / "bayesianquilts"/ "bayesianquilts"/ "data" / "overiany.csv", header=None)


X_scaled = (X_ - X_.mean())/X_.std()
X_scaled = X_scaled.fillna(0).to_numpy()
y_ = y_.to_numpy()
N = X_scaled.shape[0]
d = X_scaled.shape[1]

print((N, d))
```

```
(54, 1536)
```

In [4]:
```
logistic_horseshoe_code = """
data {
  int <lower=0> N;                // number  of  observations
  int <lower=0> d;                // number  of  predictors
  array[N] int<lower=0,upper=1> y;      // outputs
  matrix[N,d] x;                  // inputs
  real <lower=0>  scale_icept;    // prior  std for  the  intercept
  real <lower=0>  scale_global;   // scale  for  the half -t prior  for  tau
  real <lower=1>  nu_global;      // degrees  of  freedom  for the half -t prior for tau
  real <lower=1> nu_local;        // degrees  of  freedom  for  the half -t priors for  lambdas
  real <lower=0>  slab_scale;     // slab  scale  for  the  regularized  horseshoe
  real <lower=0> slab_df;         // slab  degrees  of  freedom  for the  regularized horseshoe

  //int<lower=0> N_tilde;
  //matrix[N_tilde, d] x_tilde;
  //array[N_tilde] int<lower=0,upper=1> y_obs;
}
parameters {
  real  beta0;
  vector[d] z;
  real <lower=0> tau;             // global  shrinkage  parameter
  vector <lower =0>[d] lambda;    // local  shrinkage  parameter
  real <lower=0> caux;
}
transformed  parameters {
  vector <lower =0>[d] lambda_tilde;    // 'truncated ' local  shrinkage  parameter
  real <lower=0> c;                     // slab  scale
  vector[d] beta;                       // regression  coefficients
  vector[N] f;                          // latent  function  values
  c = slab_scale * sqrt(caux);
  lambda_tilde = sqrt( c^2 * square(lambda) ./ (c^2 + tau^2* square(lambda )) );
  beta = z .*  lambda_tilde*tau;
  f = beta0 + x*beta;
}
model {
  z ~ normal(0.0, 1.0); // half -t priors  for  lambdas  and tau , and  inverse -gamma  for c^2
  lambda ~ student_t(nu_local , 0.0, 1.0);
  tau ~ student_t(nu_global , 0.0, scale_global);
  caux ~ inv_gamma (0.5* slab_df , 0.5* slab_df );
  beta0 ~ normal(0.0,  scale_icept );
  y ~ bernoulli_logit(f);
}
generated quantities {
  vector[N] log_lik;
  // vector[N_tilde] loo_log_lik;

  for (nn in 1:N)
    log_lik[nn] = bernoulli_logit_lpmf(y[nn] | x[nn] * beta + beta0);

  //for (nn in 1:N_tilde)
  //  loo_log_lik[nn] = bernoulli_logit_lpmf(y_obs[nn] | x_tilde[nn] * beta + beta0);
}
"""

with open("ovarian_model.stan", 'w') as f:
  f.writelines(logistic_horseshoe_code)
```

In [5]:
```
sm = CmdStanModel(stan_file="ovarian_model.stan")
```

```
08:42:18 - cmdstanpy - INFO - compiling stan file /home/josh/workspace/bayesianquilts/ovarian_model.stan to exe file /home/josh/workspace/bayesianquilts/ovarian_model
08:42:35 - cmdstanpy - INFO - compiled model executable: /home/josh/workspace/bayesianquilts/ovarian_model
```

In [ ]:
```python
guessnumrelevcov = N / 10  # 20.
slab_scale = 2.5
scale_icept = 5.0
nu_global = 1
nu_local = 1
slab_df = 1
scale_global = guessnumrelevcov / ((d - guessnumrelevcov) * np.sqrt(N))

control = {"adapt_delta": 0.9999, "max_treedepth": 15}

# shuffle

# shuffled_ndx = np.random.shuffle(np.arange(N))

ovarian_data = {
    "N": N,
    "d": d,
    "slab_df": slab_df,
    "slab_scale": slab_scale,
    "scale_icept": scale_icept,
    "nu_global": 1,
    "nu_local": 1,
    "scale_global": scale_global,
    "y": y_.astype(int)[:, 0].tolist(),
    "x": X_scaled.tolist(),
}

import json

with open("ovarian_data.json", "w") as f:
    json.dump(ovarian_data, f)
```

In [7]:
```python
print(d)
```

```
1536
```

In [ ]:
```python
S = 1800
fit = sm.sample(
    data="ovarian_data.json",
    iter_warmup=15000,
    iter_sampling=S,
    thin=2,
    **control
)
```

In [9]:
```python
print(fit)
```

```
CmdStanMCMC: model=ovarian_model chains=4['method=sample', 'num_samples=1800', 'num_warmup=15000', 'thin=2', 'algorithm=hmc', 'engine=nuts', 'max_depth=15', 'adapt', 'engaged=1', 'delta=0.999
9']
 csv_files:
        /tmp/tmpkex9ydkx/ovarian_modelqdm05s9e/ovarian_model-20241018171301_1.csv
        /tmp/tmpkex9ydkx/ovarian_modelqdm05s9e/ovarian_model-20241018171301_2.csv
        /tmp/tmpkex9ydkx/ovarian_modelqdm05s9e/ovarian_model-20241018171301_3.csv
        /tmp/tmpkex9ydkx/ovarian_modelqdm05s9e/ovarian_model-20241018171301_4.csv
 output_files:
        /tmp/tmpkex9ydkx/ovarian_modelqdm05s9e/ovarian_model-20241018171301_0-stdout.txt
        /tmp/tmpkex9ydkx/ovarian_modelqdm05s9e/ovarian_model-20241018171301_1-stdout.txt
        /tmp/tmpkex9ydkx/ovarian_modelqdm05s9e/ovarian_model-20241018171301_2-stdout.txt
        /tmp/tmpkex9ydkx/ovarian_modelqdm05s9e/ovarian_model-20241018171301_3-stdout.txt
```

In [ ]:
```python
print(fit.diagnose())
```

```
Processing csv files: /tmp/tmpkex9ydkx/ovarian_modelqdm05s9e/ovarian_model-20241018171301_1.csv, /tmp/tmpkex9ydkx/ovarian_modelqdm05s9e/ovarian_model-20241018171301_2.csv, /tmp/tmpkex9ydkx/ovar
ian_modelqdm05s9e/ovarian_model-20241018171301_3.csv, /tmp/tmpkex9ydkx/ovarian_modelqdm05s9e/ovarian_model-20241018171301_4.csv

Checking sampler transitions treedepth.
Treedepth satisfactory for all transitions.

Checking sampler transitions for divergences.
No divergent transitions found.

Checking E-BFMI - sampler transitions HMC potential energy.
E-BFMI satisfactory.

Effective sample size satisfactory.

Split R-hat values satisfactory all parameters.

Processing complete, no problems detected.
```

In [11]:
```python
params = fit.stan_variables()
params.keys()
params['c'] = params['c'][:, tf.newaxis]
params['tau'] = params['tau'][:, tf.newaxis]
params['caux'] = params['caux'][:, tf.newaxis]
params['beta0'] = params['beta0'][:, tf.newaxis]
```

In [ ]:
```python
c = slab_scale * tf.math.sqrt(params["caux"])
lambda_tilde = tf.math.sqrt(
    c**2
    * params["lambda"] ** 2
    / (c**2 + params["tau"] ** 2 * params["lambda"] ** 2)
)
beta_fitted = params["z"] * lambda_tilde * params["tau"]
beta_fitted.shape
```

Out[ ]:
```
TensorShape([3600, 1536])
```

In [ ]:
```python
beta_fitted = np.mean(beta_fitted, axis=0, keepdims=True)
fig, ax = plt.subplots(1, 1, figsize=(8, 1))
im = ax.pcolormesh(beta_fitted, cmap='seismic_r', vmin=-0.2, vmax=0.2)
_ = plt.ylabel("covariate")

plt.colorbar(im)
plt.savefig("ovarian_mcmc_fit.pdf", bbox_inches='tight')
```

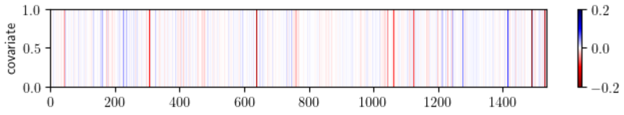

In [14]:
```python
lr_model = LogisticRegression2(
    dim_regressors=d,
    slab_df=slab_df,
    slab_scale=slab_scale,
```

```
    scale_icept=scale_icept,
    nu_global=1,
    nu_local=1,
    scale_global=scale_global,
)
```

In [15]:

```
tfdata = tf.data.Dataset.from_tensor_slices({'X': X_scaled, 'y':y_})
batch_size = N
def data_factory_factory(batch_size=batch_size, repeat=False, shuffle=False):
    def data_factory(batch_size=batch_size):
        if shuffle:
            out = tfdata.shuffle(batch_size*10)
        else:
            out = tfdata

        if repeat:
            out = out.repeat()
        return out.batch(batch_size)
    return data_factory

batch = next(iter(data_factory_factory()()))
```

In [16]:

```
def prediction_fn(data):
    pred = lr_model.predictive_distribution(data, **params)["logits"]
    return tf.reduce_mean(pred, axis=0)

bench = classification_metrics(
    data_factory=data_factory_factory(),
    prediction_fn=prediction_fn,
    outcome_label='y',
    by_vars=[]
)

fig, ax = plt.subplots(1, 2, figsize=(9, 2))
ax[0].plot(bench['auroc']['fpr'], bench['auroc']['tpr'])
ax[0].text(0.5, 0.1, f"AUROC: {round(bench['auroc']['auroc'], 2)}")
ax[0].set_xlim((0, 1))
ax[0].set_ylim((0, 1))
ax[0].set_title("ROC")

ax[1].plot(bench['auprc']['recall'], bench['auprc']['precision'])
ax[1].text(0.5, 0.8, f"AUPRC: {round(bench['auprc']['auprc'], 2)}")
ax[1].set_title("Precision-Recall")
ax[1].set_xlim((0, 1))
ax[1].set_ylim((0, 1))
```

```
0it [00:00, ?it/s]2024-10-18 17:42:41.606010: W external/local_tsl/tsl/framework/cpu_allocator_impl.cc:83] Allocation of 2388787200 exceeds 10% of free system memory.
1it [00:00,  1.57it/s]
```

Out[16]: (0.0, 1.0)

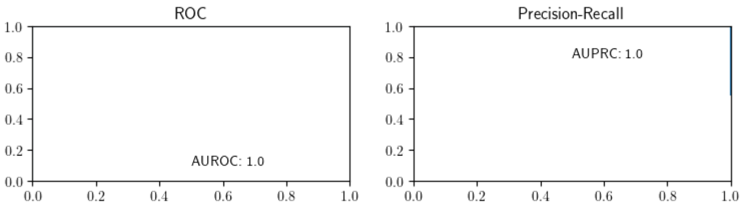

In [ ]:

```
def entropy(probs):
    return -tf.math.xlogy(probs, probs)


def adaptive_is_loo(
    self: LogisticRegression2,
    data: dict[str, tf.Tensor],
    params,
    hbar=1.0,
    variational=True,
) -> dict[str, Any]:
    """_summary_

    Args:
        data (_type_): _description_
        params (_type_): _description_
        hbar (float, optional): _description_. Defaults to 1.0.
        variational (bool, optional):
            Should we trust the variational approximation?
            If False, assumes that one is passing in all the data at once in a single batch.
            Defaults to True.

    Returns:
        _type_: _description_
    """

    # scaled (theta - bar(theta))/Sigma
    c = self.slab_scale * tf.math.sqrt(params["caux"])
    lambda_tilde = tf.math.sqrt(
        c**2
        * params["lambda"] ** 2
        / (c**2 + params["tau"] ** 2 * params["lambda"] ** 2)
    )
    beta = params["z"] * lambda_tilde * params["tau"]
    intercept = params["beta0"]
    _X = tf.cast(data["X"], self.dtype)
    _y = tf.cast(data["y"], self.dtype)[:, 0]
    mu = beta[..., tf.newaxis, :] * _X
    mu = tf.reduce_sum(mu, -1) + params["beta0"]
    sigma = tf.math.sigmoid(mu)
    ell = _y * (sigma) + (1 - _y) * (1 - sigma)
    log_ell = tf.math.xlogy(_y, sigma) + tf.math.xlogy(1 - _y, 1 - sigma)
    log_ell_prime = _y * (1 - sigma) - (1 - _y) * sigma
    log_ell_doubleprime = -sigma * (1 - sigma)
    _, khat0 = nppsis.psislw(-log_ell)

    """
    sigma.shape is samples x datapoints
    """

    ndx_to_transform = np.where(khat0 > 0.7)[0]
    ndx_to_leave = np.where((khat0 <= 0.7))[0]

    def IS(T, X, y):
        mu = beta[..., tf.newaxis, :] * X
```

```python
        mu = tf.reduce_sum(mu, -1) + params["beta0"]
        sigma = tf.math.sigmoid(mu)
        ell = y * (sigma) + (1 - y) * (1 - sigma)
        log_ell = tf.math.xlogy(y, sigma) + tf.math.xlogy(1 - y, 1 - sigma)
        log_ell_prime = y * (1 - sigma) - (1 - y) * sigma
        log_ell_doubleprime = -sigma * (1 - sigma)
        # compute # \nabla\log\pi(\btheta|\calD)
        if variational:
            # \nabla\log\pi = -\Sigma^{-1}(theta - \bar{\theta})
            grad_log_pi = tf.concat(
                [
                    -(
                        intercept
                        - self.surrogate_distribution.model["intercept__"].mean()
                    )
                    / self.surrogate_distribution.model["intercept__"].variance(),
                    -(beta - self.surrogate_distribution.model["beta__"].mean())
                    / self.surrogate_distribution.model["beta__"].variance(),
                ],
                axis=-1,
            )
            intercept_sd = (
                self.surrogate_distribution.model["intercept__"].variance() ** 0.5
            )
            beta_sd = self.surrogate_distribution.model["beta__"].variance() ** 0.5

            log_pi = self.surrogate_distribution.model["beta__"].log_prob(
                params["beta__"]
            ) + self.surrogate_distribution.model["intercept__"].log_prob(
                params["intercept__"]
            )
            log_pi -= tf.reduce_max(log_pi, axis=0)
            # log_pi.shape: [samples]
        else:
            """
            Recall Bayes rule:
            \log pi(\btheta|\calD) = \sum_i\log ell_i(\btheta) + \log\pi(\btheta) + const

            so
            \nabla\log\pi(\btheta|\calD) = \sum_i (ell_i)'x + grad\log\pi(\btheta)

            """
            log_prior = self.prior_distribution.log_prob_parts(params)
            log_prior = log_prior["z"] + log_prior["beta0"]

            log_pi = tf.reduce_sum(log_ell, axis=1, keepdims=True)[:, 0]

            # pi \propto
            grad_log_pi = tf.concat(
                [
                    tf.reduce_sum(
                        log_ell_prime[..., tf.newaxis], axis=1, keepdims=True
                    ),
                    tf.reduce_sum(
                        log_ell_prime[..., tf.newaxis] * X, axis=1, keepdims=True
                    ),
                ],
                axis=-1,
            )

            grad_log_prior = -0.5 * tf.concat(
                [(params["beta0"] / self.scale_icept) ** 2, (params["z"]) ** 2],
                axis=-1,
            )
            grad_log_pi += grad_log_prior[:, tf.newaxis, :]

            intercept_sd = tf.math.reduce_std(intercept, 0, keepdims=True)
            beta_sd = tf.math.reduce_std(beta, 0, keepdims=True)

        beta_new, intercept_new, logJ = T(
            X,
            y,
            log_pi=log_pi,
            grad_log_pi=grad_log_pi,
            beta_sd=beta_sd,
            intercept_sd=intercept_sd,
        )
        mu_new = tf.reduce_sum(beta_new * X, axis=-1) + intercept_new[..., 0]
        sigma_new = tf.math.sigmoid(mu_new)
        ell_new = y * (sigma_new) + (1 - y) * (1 - sigma_new)
        log_ell_new = tf.math.xlogy(y, sigma_new) + tf.math.xlogy(1 - y, 1 - sigma_new)
        c = self.slab_scale * tf.math.sqrt(params["caux"])
        lambda_tilde = tf.math.sqrt(
            c**2
            * params["lambda"] ** 2
            / (c**2 + params["tau"] ** 2 * params["lambda"] ** 2)
        )
        transformed = params.copy()
        transformed["z"] = beta_new / (
            lambda_tilde[:, tf.newaxis, :] * params["tau"][..., tf.newaxis]
        )
        transformed["beta0"] = intercept_new

        if variational:
            # We trust the variational approximation, so \hat{pi} = pi
            # N_samples x N_data
            delta_log_pi = (
                self.surrogate_distribution.log_prob(transformed)
                - log_pi[:, tf.newaxis]
            )
            delta_log_pi = delta_log_pi - tf.reduce_max(
                delta_log_pi, axis=0, keepdims=True
            )
            pass
        else:
            # we don't trust the variational approximation
            # Need to compute log_pi directly by summing over the likelihood

            ell_cross = tf.math.sigmoid(
                tf.reduce_sum(beta_new[..., tf.newaxis, :] * X, -1) + intercept_new
            )
            ell_cross = tf.math.xlogy(y, ell_cross) + tf.math.xlogy(
                1 - y, 1 - ell_cross
            )
            ell_cross = tf.math.reduce_sum(ell_cross, axis=-1)

            log_prior_new = self.prior_distribution.log_prob_parts(transformed)
            log_prior_new = log_prior_new["z"] + log_prior_new["beta0"]
            log_pi_new = ell_cross
```

```python
            delta_log_prior = log_prior_new - log_prior[:, tf.newaxis]
            # Incorporate the prior
            delta_log_pi = log_pi_new - log_pi[:, tf.newaxis] + delta_log_prior

        # regularize delta_log_pi

        def regularize(tensor):
            min_finite = tf.reduce_min(
                tf.where(
                    tf.math.is_finite(tensor),
                    tensor,
                    tf.zeros_like(tensor),
                )
            )
            return tf.where(
                tf.math.is_finite(tensor),
                tensor,
                min_finite * tf.ones_like(tensor),
            )

        delta_log_pi = regularize(delta_log_pi)
        delta_log_pi -= tf.reduce_max(delta_log_pi, axis=0)
        log_eta_weights = delta_log_pi - regularize(log_ell_new) + logJ
        log_eta_weights = log_eta_weights - tf.reduce_max(log_eta_weights, axis=0)
        psis_weights, khat = nppsis.psislw(log_eta_weights)

        eta_weights = tf.math.exp(log_eta_weights)
        eta_weights = eta_weights / tf.reduce_sum(eta_weights, axis=0, keepdims=True)

        psis_weights = tf.math.exp(psis_weights)
        psis_weights = psis_weights / tf.math.reduce_sum(
            psis_weights, axis=0, keepdims=True
        )

        weight_entropy = self.entropy(eta_weights)
        psis_entropy = self.entropy(psis_weights)

        p_loo_new = tf.reduce_sum(sigma_new * eta_weights, axis=0)
        p_loo_psis = tf.reduce_sum(sigma_new * psis_weights, axis=0)
        p_loo_sd = tf.math.reduce_std(sigma_new * eta_weights, axis=0)
        ll_loo_new = tf.reduce_sum(eta_weights * ell_new, axis=0)
        ll_loo_psis = tf.reduce_sum(psis_weights * ell_new, axis=0)
        ll_loo_sd = tf.math.reduce_std(eta_weights * ell_new, axis=0)
        return (
            eta_weights,
            psis_weights,
            p_loo_new,
            p_loo_sd,
            ll_loo_new,
            ll_loo_sd,
            weight_entropy,
            khat,
            p_loo_psis,
            ll_loo_psis,
        )

    # log-likelihood descent

    def T_ll(X, y, log_pi, beta_sd, intercept_sd, **kwargs):
        mu = beta[..., tf.newaxis, :] * X
        mu = tf.reduce_sum(mu, -1) + params["beta0"]
        sigma = tf.math.sigmoid(mu)

        log_ell_prime = y * (1 - sigma) - (1 - y) * sigma

        Q_beta = -log_ell_prime[..., tf.newaxis] * X
        Q_intercept = -log_ell_prime[..., tf.newaxis]

        standardized = tf.concat(
            [Q_beta / beta_sd, Q_intercept / intercept_sd], axis=-1
        )
        standardized = tf.reduce_max(tf.math.abs(standardized), axis=-1)
        standardized = tf.reduce_max(standardized, axis=0, keepdims=True)[
            ..., tf.newaxis
        ]

        h = hbar / standardized
        logJ = tf.math.log1p(
            tf.math.abs(
                h
                * (1 + tf.math.reduce_sum(X**2, -1, keepdims=True))[tf.newaxis, :, :]
                * (sigma * (1 - sigma))[..., tf.newaxis]
            )[..., 0]
        )
        beta_ll = beta[..., tf.newaxis, :] + h * Q_beta
        intercept_ll = intercept[..., tf.newaxis, :] + h * Q_intercept
        return beta_ll, intercept_ll, logJ

    def T_kl(X, y, log_pi, grad_log_pi, beta_sd, intercept_sd):
        log_pi_ = log_pi - tf.reduce_max(log_pi, axis=0, keepdims=True)
        mu = beta[..., tf.newaxis, :] * X
        mu = tf.reduce_sum(mu, -1) + params["beta0"]
        Q_beta = ((-1) ** y * tf.math.exp(log_pi_[..., tf.newaxis] + mu * (1 - 2 * y)))[
            ..., tf.newaxis
        ] * X
        Q_intercept = (
            ((-1) ** y) * tf.math.exp(log_pi_[..., tf.newaxis] + mu * (1 - 2 * y))
        )[..., tf.newaxis]

        dQ = (-1) ** y[tf.newaxis, :] * tf.math.exp(
            log_pi_[..., tf.newaxis] + mu * (1 - 2 * y[tf.newaxis, :])
        )
        dQ *= (
            grad_log_pi[..., 0]
            + (1 - 2 * y)[tf.newaxis, :]
            + tf.reduce_sum(
                X
                * (
                    grad_log_pi[..., 1:]
                    + (1 - 2 * y)[:, tf.newaxis] * X[tf.newaxis, ...]
                ),
                axis=-1,
            )
        )

        standardized = tf.concat(
            [Q_beta / beta_sd, Q_intercept / intercept_sd], axis=-1
        )
        standardized = tf.reduce_max(tf.math.abs(standardized), axis=-1)
```

```
        standardized = tf.reduce_max(standardized, axis=0, keepdims=True)[
            ..., tf.newaxis
        ]

        h = hbar / standardized

        intercept_kl = intercept[..., tf.newaxis] + h * Q_intercept
        beta_kl = beta[..., tf.newaxis, :] + h * Q_beta

        logJ = tf.math.log1p(tf.math.abs(h[..., 0] * dQ))
        return beta_kl, intercept_kl, logJ

    def T_I(X, y, **kwargs):
        mu = beta[..., tf.newaxis, :] * X
        mu = tf.reduce_sum(mu, -1) + params["beta0"]
        sigma = tf.math.sigmoid(mu)

        log_ell = tf.math.xlogy(y, sigma) + tf.math.xlogy(1 - y, 1 - sigma)
        Q = tf.zeros_like(log_ell)
        return (
            beta[:, tf.newaxis, :] + Q[..., tf.newaxis],
            intercept[..., tf.newaxis] + Q[..., tf.newaxis],
            tf.zeros_like(Q),
        )

    def T_var(X, y, log_pi, grad_log_pi, beta_sd, intercept_sd, **kwargs):
        log_pi_ = log_pi - tf.reduce_max(log_pi, axis=0, keepdims=True)
        mu = beta[..., tf.newaxis, :] * X
        mu = tf.reduce_sum(mu, -1) + params["beta0"]
        Q_beta = (
            (-1) ** y * tf.math.exp(log_pi_[..., tf.newaxis] + 2 * mu * (1 - 2 * y))
        )[..., tf.newaxis] * X
        Q_intercept = (
            (-1) ** y * tf.math.exp(log_pi_[..., tf.newaxis] + 2 * mu * (1 - 2 * y))
        )[..., tf.newaxis]

        dQ = (
            (-1) ** y[tf.newaxis, :]
            * tf.math.exp(
                log_pi_[..., tf.newaxis] + 2 * mu * (1 - 2 * y[tf.newaxis, :])
            )
            * (
                grad_log_pi[..., 0]
                + (1 - 2 * y)[tf.newaxis, :]
                + tf.reduce_sum(
                    X * (grad_log_pi[..., 1:] + 2 * (1 - 2 * y)[:, tf.newaxis] * X),
                    axis=-1,
                )
            )
        )

        standardized = tf.concat(
            [Q_beta / beta_sd, Q_intercept / intercept_sd], axis=-1
        )
        standardized = tf.reduce_max(tf.math.abs(standardized), axis=-1)
        standardized = tf.reduce_max(standardized, axis=0, keepdims=True)[
            ..., tf.newaxis
        ]

        h = hbar / standardized

        intercept_kl = intercept[..., tf.newaxis, :] + h * Q_intercept
        beta_kl = beta[..., tf.newaxis, :] + h * Q_beta

        logJ = tf.math.log1p(tf.math.abs(h[..., 0] * dQ))
        return beta_kl, intercept_kl, logJ

    def T_MM1(X, y, **kwargs):
        c = self.slab_scale * tf.math.sqrt(params["caux"])
        lambda_tilde = tf.math.abs(c) / tf.math.sqrt(
            (c / params["lambda"]) ** 2 + params["tau"] ** 2
        )
        beta = params["z"] * lambda_tilde * params["tau"]
        mu = beta[..., tf.newaxis, :] * X
        mu = tf.reduce_sum(mu, -1) + params["beta0"]
        sigma = tf.math.sigmoid(mu)
        ell = y * (sigma) + (1 - y) * (1 - sigma)  # S x N

        weights = (
            1
            / ell[..., tf.newaxis]
            / tf.reduce_sum(1 / ell[..., tf.newaxis], axis=0, keepdims=True)
        )

        def theta_hat(param):  # param is S x K
            hat = tf.reduce_mean(param[:, tf.newaxis, ...], axis=0, keepdims=True)
            hat_w = param[:, tf.newaxis, ...] * weights
            hat_w = tf.reduce_sum(hat_w, axis=0, keepdims=True)
            v = tf.reduce_mean((param[:, tf.newaxis, ...] - hat) ** 2)
            v_w = tf.reduce_sum(
                weights * (param[:, tf.newaxis, ...] - hat) ** 2, axis=0, keepdims=True
            )
            return hat, hat_w, v, v_w

        beta_hat, beta_hat_w, beta_v, beta_v_w = theta_hat(beta)
        beta0_hat, beta0_hat_w, beta0_v, beta0_v_w = theta_hat(params["beta0"])

        # tf.math.exp(0.5*(tf.math.log(beta_v_w) - tf.math.log(beta_v)))-tf.math.sqrt(beta_v_w / beta_v)

        beta_adj = beta[:, tf.newaxis, ...] + hbar * (-beta_hat + beta_hat_w)
        beta0_adj = params["beta0"][:, tf.newaxis, ...] + hbar * (
            -beta0_hat + beta0_hat_w
        )

        # shift by mean
        return beta_adj, beta0_adj, tf.zeros_like(ell)

    def T_MM2(X, y, **kwargs):
        c = self.slab_scale * tf.math.sqrt(params["caux"])
        lambda_tilde = tf.math.abs(c) / tf.math.sqrt(
            (c / params["lambda"]) ** 2 + params["tau"] ** 2
        )
        beta = params["z"] * lambda_tilde * params["tau"]
        mu = beta[..., tf.newaxis, :] * X
        mu = tf.reduce_sum(mu, -1) + params["beta0"]
        sigma = tf.math.sigmoid(mu)
        ell = y * (sigma) + (1 - y) * (1 - sigma)  # S x N

        weights = (
```

```
            1
            / ell[..., tf.newaxis]
            / tf.reduce_sum(1 / ell[..., tf.newaxis], axis=0, keepdims=True)
        )

        def theta_hat(param):  # param is S x K
            hat = tf.reduce_mean(param[:, tf.newaxis, ...], axis=0, keepdims=True)
            hat_w = param[:, tf.newaxis, ...] * weights
            hat_w = tf.reduce_sum(hat_w, axis=0, keepdims=True)
            v = tf.reduce_mean((param[:, tf.newaxis, ...] - hat) ** 2)
            v_w = tf.reduce_sum(
                weights * (param[:, tf.newaxis, ...] - hat) ** 2, axis=0, keepdims=True
            )
            return hat, hat_w, v, v_w

        beta_hat, beta_hat_w, beta_v, beta_v_w = theta_hat(beta)
        beta0_hat, beta0_hat_w, beta0_v, beta0_v_w = theta_hat(params["beta0"])

        # tf.math.exp(0.5*(tf.math.log(beta_v_w) - tf.math.log(beta_v)))-tf.math.sqrt(beta_v_w / beta_v)

        beta_adj = beta[:, tf.newaxis, ...] + hbar * (
            (tf.math.sqrt(beta_v_w / beta_v) - 1) * beta[:, tf.newaxis, ...]
            - tf.math.sqrt(beta_v_w / beta_v) * beta_hat
            + beta_hat_w
        )
        beta0_adj = params["beta0"][:, tf.newaxis, ...] + hbar * (
            (tf.math.sqrt(beta0_v_w / beta0_v) - 1)
            * params["beta0"][:, tf.newaxis, ...]
            - tf.math.sqrt(beta0_v_w / beta0_v) * beta0_hat
            + beta0_hat_w
        )

        lJ = tf.math.log(
            1
            + hbar
            * (
                beta.shape[-1]
                * tf.ones_like(ell)
                * (tf.math.sqrt(beta0_v_w / beta0_v) - 1)[..., 0]
            )
        )
        lJ += tf.math.log(1 + hbar * (tf.math.sqrt(beta0_v_w / beta0_v) - 1)[..., 0])

        # shift by mean
        return beta_adj, beta0_adj, lJ

    X_ = tf.gather(_X, ndx_to_leave)
    y_ = tf.gather(_y, ndx_to_leave)
    (
        eta_I,
        eta_I_psis,
        p_loo_I,
        p_loo_I_sd,
        ll_loo_I,
        ll_loo_I_sd,
        S_I,
        k_I,
        p_psis_I,
        ll_psis_I,
    ) = IS(T_I, X_, y_)

    out = {
        "I0": {
            "p_loo": p_loo_I,
            "p_loo_sd": p_loo_I_sd,
            "ll_loo": ll_loo_I,
            "ll_loo_sd": ll_loo_I_sd,
            "S": S_I,
            "khat": k_I,
            "p_psis": p_psis_I,
            "ll_psis": ll_psis_I,
            "ndx": ndx_to_leave,
        }
    }

    transforms = {
        "MM1": T_MM1,
        "MM2": T_MM2,
        "I": T_I,
        "KL": T_kl,
        "Var": T_var,
        "LL": T_ll,
    }

    X_ = tf.gather(_X, ndx_to_transform)
    y_ = tf.gather(_y, ndx_to_transform)
    for lab, fun in transforms.items():
        (
            eta_,
            eta_psis_,
            p_loo_,
            p_loo_sd_,
            ll_loo_,
            ll_loo_s_d,
            S_,
            k_,
            p_psis_,
            ll_psis_,
        ) = IS(fun, X_, y_)

        out = {
            **out,
            lab: {
                "p_loo": p_loo_,
                "p_loo_sd": p_loo_sd_,
                "ll_loo": ll_loo_,
                "ll_loo_sd": ll_loo_s_d,
                "S": S_,
                "khat": k_,
                "p_psis": p_psis_,
                "ll_psis": ll_psis_,
                "ndx": ndx_to_transform,
            },
        }

    return {**out, "ndx_transformed": ndx_to_transform}


nparams = 64
```

```python
loo = adaptive_is_loo(
    lr_model,
    batch,
    {k: v[:nparams, ...] for k, v in params.items()},
    1e-5,
    variational=False,
)

# loo = adaptive_is_loo(lr_model, test_batch, param_test, 0.01, variational=False)


for T in ["I", "LL", "KL", "Var", "MM1", "MM2"]:
    print(
        f"{T}: {np.sqrt(np.sum(loo[T]['p_loo_sd']**2))} entropy: {np.sqrt(np.sum(loo[T]['S']))} khat>0.7: {np.sum(loo[T]['khat']>0.7)}"
    )
```

```
I: 0.07711837689332257 entropy: 8.969121307221462 khat>0.7: 21
LL: 0.07762175810916011 entropy: 8.96801912939596 khat>0.7: 17
KL: 0.07663946525876737 entropy: 8.971062943369231 khat>0.7: 21
Var: 0.07667947516120609 entropy: 8.97159062957394 khat>0.7: 20
MM1: 0.07661524503411589 entropy: 8.919609574679024 khat>0.7: 18
MM2: 0.08668591151068142 entropy: 8.644117750969603 khat>0.7: 13
```

In [18]:

```python
base = 4
h_vals = [base**-r for r in range(11)]

loo_output = []

for _ in tqdm(range(1)):
    loo_khat = {}
    reduced_ndx = {}
    high_khat_ndx = {}
    n_sample = [1000]
    records = []
    for n_samples in n_sample:
        sample_ndx = np.random.choice(range(fit.stan_variables()['beta0'].shape[0]), size=n_samples, replace=False)
        reduced_ndx[n_samples] = {}
        high_khat_ndx[n_samples] = {}
        # print(f"Samples: {n_samples}")
        loo_khat[n_samples] = {}
        params_ = fit.stan_variables()
        params_ = {k: v[sample_ndx] for k, v in params_.items()}

        params_["c"] = params_["c"][:, tf.newaxis]
        params_["tau"] = params_["tau"][:, tf.newaxis]
        params_["caux"] = params_["caux"][:, tf.newaxis]
        params_["beta0"] = params_["beta0"][:, tf.newaxis]

        for h in h_vals:
            loo_khat[n_samples][h] = {}
            loo = adaptive_is_loo(lr_model, batch, params_, h, variational=False)
            reduced_ndx[n_samples][h] = {}
            # print(f"rho={h}\n")
            for T in ["I0", "I", "LL", "KL", "Var", "MM1", "MM2"]:
                loo_khat[n_samples][h][T] = np.array(loo[T]["khat"])

                records += [
                    {
                        "h": h,
                        "T": T,
                        "S": n_samples,
                        "khat": loo[T]["khat"],
                        "p_psis": loo[T]["p_psis"].numpy(),
                        "n>0.7": (np.where((loo[T]["khat"] > 0.7))[0]).shape,
                        "ndx": loo[T]["ndx"]
                    }
                ]
                reduced_ndx[n_samples][h][T] = [loo[T]["ndx"][k] for k in np.where((loo[T]["khat"] <= 0.7))[0]]
                # print(reduced_ndx[n_samples][h])


print(reduced_ndx)
```

```
  0%|          | 0/1 [00:00<?, ?it/s]2024-10-18 17:44:41.808201: W external/local_tsl/tsl/framework/cpu_allocator_impl.cc:83] Allocation of 3981312000 exceeds 10% of free system memory.
2024-10-18 17:44:43.063752: W external/local_tsl/tsl/framework/cpu_allocator_impl.cc:83] Allocation of 15925248000 exceeds 10% of free system memory.
2024-10-18 17:44:46.797639: W external/local_tsl/tsl/framework/cpu_allocator_impl.cc:83] Allocation of 15925248000 exceeds 10% of free system memory.
2024-10-18 17:44:49.744335: W external/local_tsl/tsl/framework/cpu_allocator_impl.cc:83] Allocation of 15925248000 exceeds 10% of free system memory.
100%|██████████| 1/1 [03:43<00:00, 223.55s/it]
{1000: {1: {'I0': [2, 3, 4, 5, 7, 9, 10, 11, 12, 13, 14, 16, 19, 34, 35, 42, 46, 52], 'I': [], 'LL': [32], 'KL': [44], 'Var': [23, 44], 'MM1': [], 'MM2': []}, 0.25: {'I0': [2, 3, 4, 5, 7, 9, 1
0, 11, 12, 13, 14, 16, 19, 34, 35, 42, 46, 52], 'I': [], 'LL': [27, 32], 'KL': [22, 26, 44], 'Var': [18, 20, 44], 'MM1': [49, 50, 53], 'MM2': []}, 0.0625: {'I0': [2, 3, 4, 5, 7, 9, 10, 11, 12,
13, 14, 16, 19, 34, 35, 42, 46, 52], 'I': [], 'LL': [44], 'KL': [0, 31, 44, 53], 'Var': [0, 18, 20, 22, 23, 31, 39, 40, 44], 'MM1': [28, 36], 'MM2': []}, 0.015625: {'I0': [2, 3, 4, 5, 7, 9, 10,
11, 12, 13, 14, 16, 19, 34, 35, 42, 46, 52], 'I': [], 'LL': [0, 23, 44], 'KL': [6, 8, 18, 22, 23, 40], 'Var': [0, 18, 20, 22, 23, 24, 30, 39, 44], 'MM1': [8, 15, 18, 21, 25, 26, 37, 38, 41, 43,
45, 48, 53], 'MM2': []}, 0.00390625: {'I0': [2, 3, 4, 5, 7, 9, 10, 11, 12, 13, 14, 16, 19, 34, 35, 42, 46, 52], 'I': [], 'LL': [17, 18], 'KL': [17, 18, 20, 32, 36, 40, 44], 'Var': [20, 22, 23,
30, 39, 43], 'MM1': [22, 27, 36, 40, 41], 'MM2': []}, 0.0009765625: {'I0': [2, 3, 4, 5, 7, 9, 10, 11, 12, 13, 14, 16, 19, 34, 35, 42, 46, 52], 'I': [], 'LL': [31, 51], 'KL': [17, 18, 23], 'Va
r': [0, 1, 18, 20, 23, 30, 44, 51], 'MM1': [6, 20, 24, 26, 27, 29, 30, 36, 45, 48, 51, 53], 'MM2': []}, 0.000244140625: {'I0': [2, 3, 4, 5, 7, 9, 10, 11, 12, 13, 14, 16, 19, 34, 35, 42, 46, 5
2], 'I': [], 'LL': [0, 22, 24], 'KL': [0, 22, 23, 31], 'Var': [0, 23, 31], 'MM1': [8, 17, 21, 26, 28, 29, 32, 37, 39, 40, 45, 47, 50, 53], 'MM2': [6, 8, 15, 17, 21, 22, 25, 27, 28, 29, 33, 36,
37, 38, 39, 40, 41, 43, 45, 47, 48, 49, 50, 51, 53]}, 6.103515625e-05: {'I0': [2, 3, 4, 5, 7, 9, 10, 11, 12, 13, 14, 16, 19, 34, 35, 42, 46, 52], 'I': [], 'LL': [0], 'KL': [0, 23], 'Var': [23],
'MM1': [21, 22, 24, 31, 37, 38, 40, 41, 43, 50, 51], 'MM2': [0, 6, 8, 15, 17, 18, 21, 22, 24, 25, 26, 27, 28, 29, 33, 36, 37, 38, 39, 40, 41, 43, 45, 47, 48, 49, 50, 51, 53]}, 1.52587890625e-0
5: {'I0': [2, 3, 4, 5, 7, 9, 10, 11, 12, 13, 14, 16, 19, 34, 35, 42, 46, 52], 'I': [], 'LL': [], 'KL': [23], 'Var': [23], 'MM1': [27, 36, 43], 'MM2': []}, 3.814697265625e-06: {'I0': [2, 3, 4,
5, 7, 9, 10, 11, 12, 13, 14, 16, 19, 34, 35, 42, 46, 52], 'I': [], 'LL': [23], 'KL': [], 'Var': [], 'MM1': [23], 'MM2': []}, 9.5367431640625e-07: {'I0': [2, 3, 4, 5, 7, 9, 10, 11, 12, 13, 14, 1
6, 19, 34, 35, 42, 46, 52], 'I': [], 'LL': [], 'KL': [], 'Var': [], 'MM1': [45], 'MM2': [0, 6, 8, 17, 18, 21, 22, 25, 27, 37, 39, 43, 47, 48, 50, 51, 53]}}}
```

Look at S=1000 case

In [19]:

```python
df = pd.DataFrame(records)
df['rho_rank'] = np.emath.logn(base, df['h'])
df.head()
I0_ = df.loc[(df['S']==n_sample[-1]) & (df['T']=="I0")]

kl_ = df.loc[(df['S']==n_sample[-1]) & (df['T']=="KL")]
var_ = df.loc[(df['S']==n_sample[-1]) & (df['T']=="Var")]
ll_ = df.loc[(df['S']==n_sample[-1]) & (df['T']=="LL")]
mm1_ = df.loc[(df['S']==n_sample[-1]) & (df['T']=="MM1")]
I_ = df.loc[(df['S']==n_sample[-1]) & (df['T']=="I")]

kl_ = kl_.explode(['khat', 'ndx'])
var_ = var_.explode(['khat', 'ndx'])
ll_ = ll_.explode(['khat', 'ndx'])
I_ = I_.explode(['khat', 'ndx'])
mm1_ = mm1_.explode(['khat', 'ndx'])

original = I_[['khat', 'ndx']].drop_duplicates()
```

In [ ]:

```python
fig = plt.figure(figsize=(4, 7))
ndx_high_khat = loo['KL']['ndx'].tolist()
ymax = (len(ndx_high_khat))
_ = plt.scatter(original.khat, np.array([ndx_high_khat.index(i) for i in original['ndx']]) ,color="black", s=12, alpha=0.6)
_ = plt.scatter(kl_.khat, np.array([ndx_high_khat.index(i) for i in kl_['ndx']]) + 0.2, s=1, color='green', alpha=0.2)
```

```
_ = plt.scatter(var_.khat, np.array([ndx_high_khat.index(i) for i in var_['ndx']]) - 0.2, s=1, color='purple', alpha=0.2)

kl_ = kl_[kl_.khat<3]
ll_ = ll_[ll_.khat<3]
var_ = var_[var_.khat<3]
_ = plt.axvline(x=0.7, linestyle='dashed', linewidth=2, color='red', alpha=0.5)

kl_vals = defaultdict(list)
var_vals = defaultdict(list)
ll_vals = defaultdict(list)

for x, y, s in zip( kl_.khat, kl_['ndx'], kl_['rho_rank'].values.astype('int')):
    kl_vals[y] += [x]

for x, y, s in zip( var_.khat, var_['ndx'], var_['rho_rank'].values.astype('int')):
    var_vals[y] += [x]

for x, v in kl_vals.items():
    plt.plot(min(v), ndx_high_khat.index(x)+0.2, color='green', alpha=0.3)
    plt.text(min(v), ndx_high_khat.index(x) + 0.2, "KL",  ha='center', va='center', color='green', fontsize=8)

for x, v in var_vals.items():
    plt.plot(min(v), ndx_high_khat.index(x)-0.2, color='purple', alpha=0.3)
    plt.text(min(v), ndx_high_khat.index(x)-0.2, "Var",  ha='center', va='center', color='blue', fontsize=8)


#for x, v in var_vals.items():
#    plt.text(min(v), x, "LL",  ha='center', va='center', color='purple', fontsize=8)

_ = plt.ylabel("observation")
_ = plt.ylim(-0.5, ymax - 0.5)
_ = plt.xlabel(r'estimated Pareto tail shape $\hat{k}$')
_ = plt.yticks(ticks=np.arange(ymax), labels=loo['KL']['ndx'])
_ = plt.xlim((-0.5, 2))
_ = plt.xticks([-0.5, 0, 0.7, 1.5, 2])


for y0, y1 in zip(np.arange(ymax+ 1)[::2]-0.5, np.arange(ymax + 1)[::2] + 0.5):
    plt.axhspan(y0, y1, color='black', alpha=0.1, zorder=0)
#_ = plt.xlim((-0.1, 2))
_ = plt.savefig("khat.pdf", bbox_inches='tight')
```

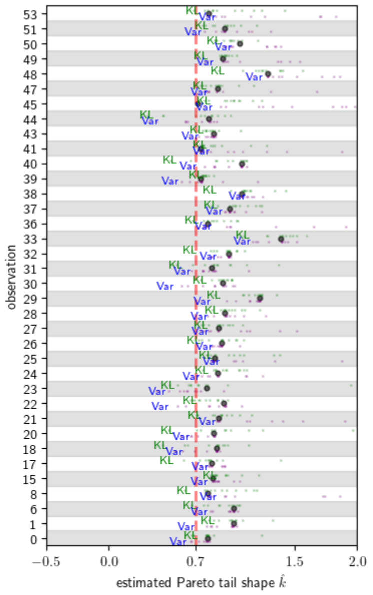

In [21]:
```
len(ndx_high_khat)
```

Out[21]: 36

In [ ]:
```
fig = plt.figure(figsize=(6, 9))

_ = plt.scatter(original.khat, np.array([ndx_high_khat.index(i) for i in original['ndx']]) ,color="black", s=12, alpha=0.6)
_ = plt.scatter(kl_.khat, np.array([ndx_high_khat.index(i) for i in kl_['ndx']]), s=0, color='green', alpha=0.2)
_ = plt.scatter(var_.khat, np.array([ndx_high_khat.index(i) for i in var_['ndx']]), s=0, color='purple', alpha=0.2)
_ = plt.scatter(ll_.khat, np.array([ndx_high_khat.index(i) for i in ll_['ndx']]), s=0, color='green', alpha=0.2)
_ = plt.scatter(mm1_.khat, np.array([ndx_high_khat.index(i) for i in mm1_['ndx']]), s=0, color='purple', alpha=0.2)

kl_ = kl_[kl_.khat<2]
ll_ = ll_[ll_.khat<2]
var_ = var_[var_.khat<2]
mm1_ = mm1_[mm1_.khat<2]
_ = plt.axvline(x=0.7, linestyle='dotted', linewidth=2, color='red')

for x, y, s in zip( kl_.khat, kl_['ndx'], kl_['rho_rank'].values.astype('int')):
    if s > 7:
        continue
    plt.text(x, ndx_high_khat.index(y) + 0.2, str(s),  ha='center', va='center', color='green', fontsize=8)

for x, y, s in zip( var_.khat, var_['ndx'], var_['rho_rank'].values.astype('int')):
    if s > 7:
        continue
    plt.text(x, ndx_high_khat.index(y) + 0.1, str(s),  ha='center', va='center', color='blue', fontsize=8)

for x, y, s in zip( ll_.khat, ll_['ndx'], ll_['rho_rank'].values.astype('int')):
    if s > 3:
        continue
    plt.text(x, ndx_high_khat.index(y) - 0.1, str(s),  ha='center', va='center', color='orange', fontsize=8)

for x, y, s in zip( mm1_.khat, mm1_['ndx'], mm1_['rho_rank'].values.astype('int')):
    if s > 3:
        continue
    plt.text(x, ndx_high_khat.index(y) - 0.2, str(s),  ha='center', va='center', color='red', fontsize=8)

_ = plt.ylabel("observation")
_ = plt.ylim(-0.5, ymax - 0.5)
_ = plt.xlabel(r'estimated Pareto tail shape $\hat{k}$')
```

```
_ = plt.yticks(ticks=np.arange(ymax), labels=loo['KL']['ndx'])
_ = plt.xlim((-0.5, 2))
_ = plt.xticks([-0.5, 0, 0.7, 1.5, 2])


for y0, y1 in zip(np.arange(ymax+ 1)[::2]-0.5, np.arange(ymax + 1)[::2] + 0.5):
    plt.axhspan(y0, y1, color='black', alpha=0.1, zorder=0)
#_ = plt.xlim((-0.1, 2))
_ = plt.savefig("khat_detailed.pdf", bbox_inches='tight')
```

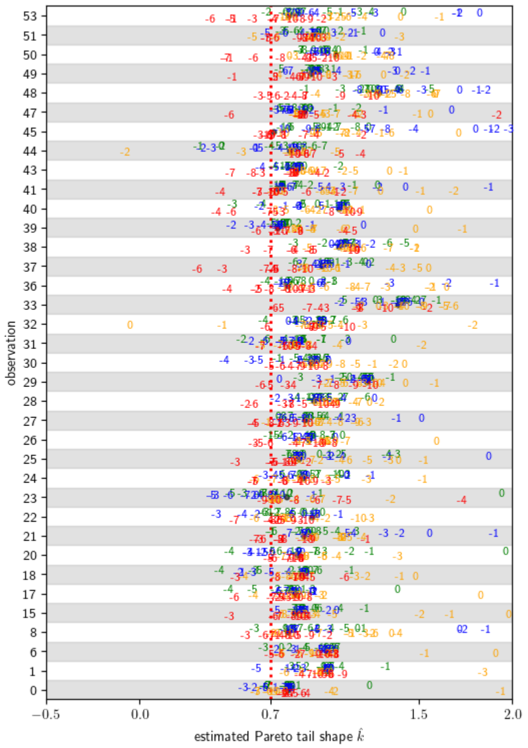

In [23]:
```
df_ = df.explode(['khat', "p_psis", 'ndx'])[['ndx', 'p_psis', 'khat', 'T']]
#df_ = df_[df_['p_psis']!=1]
#df_ = df_[df_['p_psis']>1e-5]
df_ = df_[df_['T']!="MM2"]

df_ = df_.groupby(['ndx'])[['khat', 'p_psis']].min()

pd.set_option('display.max_rows', 500)

df_ = df_.reset_index()
df_['y'] = [y_.tolist()[i][0] for i in df_['ndx'].values]
```

In [24]:
```
fpr, tpr, thresholds = metrics.roc_curve(df_['y'], df_.p_psis, pos_label=1)
precision, recall, thresholds_pr = metrics.precision_recall_curve(df_['y'], df_.p_psis)
```

Cross-tabulations

In [25]:
```
df__ = df.explode(['khat', 'ndx'])[['ndx', 'khat', 'T']]
transforms = ["KL", "Var", "LL", "MM1", "MM2"]
```

In [ ]:
```
for T in transforms:
    _df__ = df__.loc[(df__['T']==T) & (df__['khat']<0.7)]
    print(f"{T}: {_df__.groupby('ndx').min().shape[0]}")
print(len(df__[df__['T']!="I0"].ndx.unique()))
```

```
KL: 15
Var: 14
LL: 11
MM1: 32
MM2: 29
36
```

In [27]:
```
print(f"{df__.loc[((df__['T']=='KL') | (df__['T']=='Var')) & (df__['khat']<0.7)].groupby('ndx').min().shape[0]}")
```

```
21
```

In [28]:
```
print(f"{df__.loc[((df__['T']=='LL') | (df__['T']=='MM1')) &(df__['T']!='I0') & (df__['khat']<0.7)].groupby('ndx').min().shape[0]}")
```

```
34
```

In [29]:
```
print(f"{df__.loc[(df__['T']!='MM1') &(df__['T']!='I0') & (df__['khat']<0.7)].groupby('ndx').min().shape[0]}")
```

```
36
```

In [30]:
```
len(df__[df__['T']!="I0"].ndx.unique())
```

Out[30]:
```
36
```

Compare with variational model

In [31]:
```
lr_model2 = LogisticRegression2(
    dim_regressors=d,
    slab_df=slab_df,
    slab_scale=slab_scale,
    scale_icept=scale_icept,
    nu_global=1,
    nu_local=1,
    scale_global=scale_global,
)
```

In [ ]:
```python
losses = lr_model2.fit(
    data_factory_factory(shuffle=True, repeat=True),
    dataset_size=N,
    batches_per_step=9,
    check_every=int(N/batch_size)*50,
    batch_size=batch_size,
    num_steps=12000,
    max_decay_steps=10,
    max_plateau_epochs=100,
    sample_size=36,
    learning_rate=0.0005,
    lr_decay_factor=0.9
    )
```

In [33]:
```python
base = 4
h_vals = [4**-r for r in range(9)]

loo_output = []

for _ in tqdm(range(1)):
    loo_khat_advi = {}
    reduced_ndx_advi = {}
    n_sample = [1000]
    records_advi = []
    for n_samples in n_sample:
        sample_ndx = np.random.choice(range(fit.stan_variables()['beta0'].shape[0]), size=n_samples, replace=False)
        reduced_ndx_advi[n_samples] = {}
        # print(f"Samples: {n_samples}")
        loo_khat_advi[n_samples] = {}
        params_ = lr_model2.sample(n_samples)

        for h in h_vals:
            loo_khat_advi[n_samples][h] = {}
            loo = adaptive_is_loo(lr_model2, batch, params_, h, variational=False)
            reduced_ndx_advi[n_samples][h] = {}
            # print(f"rho={h}\n")
            for T in ["I0", "I", "LL", "KL", "Var", "MM1", "MM2"]:
                loo_khat_advi[n_samples][h][T] = np.array(loo[T]["khat"])

                records_advi += [
                    {
                        "h": h,
                        "T": T,
                        "S": n_samples,
                        "khat": loo[T]["khat"],
                        "p_psis": loo[T]["p_psis"].numpy(),
                        "n>0.7": (np.where((loo[T]["khat"] > 0.7))[0]).shape,
                        "ndx": loo[T]["ndx"]
                    }
                ]
                reduced_ndx_advi[n_samples][h][T] = [loo[T]["ndx"][k] for k in np.where((loo[T]["khat"] <= 0.7))[0]]
                # print(reduced_ndx[n_samples][h])


print(reduced_ndx_advi)
```

```
100%|██████████| 1/1 [02:33<00:00, 153.75s/it]
{1000: {1: {'I0': [2, 5, 6, 7, 8, 9, 11, 12, 13, 14, 16, 18, 22, 25, 28, 29, 32, 36, 40, 45, 48, 50], 'I': [], 'LL': [3, 10, 17, 20, 21, 23, 27, 38, 39, 46, 47, 51, 52, 53], 'KL': [0, 17, 19, 3
3], 'Var': [0, 1, 19, 20, 23, 27, 30, 44, 53], 'MM1': [], 'MM2': []}, 0.25: {'I0': [2, 5, 6, 7, 8, 9, 11, 12, 13, 14, 16, 18, 22, 25, 28, 29, 32, 36, 40, 45, 48, 50], 'I': [], 'LL': [4, 24, 30,
31, 34, 42, 43, 46], 'KL': [1, 17, 19, 20, 21, 53], 'Var': [0, 1, 17, 19, 20, 21, 44, 49, 53], 'MM1': [21, 33, 53], 'MM2': []}, 0.0625: {'I0': [2, 5, 6, 7, 8, 9, 11, 12, 13, 14, 16, 18, 22, 25,
28, 29, 32, 36, 40, 45, 48, 50], 'I': [], 'LL': [0, 1, 4, 10, 15, 33, 43, 44, 53], 'KL': [1, 17, 20, 21, 49, 53], 'Var': [1, 15, 17, 19, 20, 21, 44, 49, 53], 'MM1': [17, 21, 33, 49], 'MM2':
[]}, 0.015625: {'I0': [2, 5, 6, 7, 8, 9, 11, 12, 13, 14, 16, 18, 22, 25, 28, 29, 32, 36, 40, 45, 48, 50], 'I': [], 'LL': [17, 19, 26, 34, 43, 52, 53], 'KL': [17, 21, 23, 49, 52, 53], 'Var': [1,
17, 19, 53], 'MM1': [1, 10, 19, 23, 26, 27, 30, 38, 43, 47, 53], 'MM2': [17, 21, 30, 33, 34, 53]}, 0.00390625: {'I0': [2, 5, 6, 7, 8, 9, 11, 12, 13, 14, 16, 18, 22, 25, 28, 29, 32, 36, 40, 45,
48, 50], 'I': [], 'LL': [17, 19, 21, 23, 27, 30, 43, 49, 53], 'KL': [17, 21, 49, 53], 'Var': [17], 'MM1': [0, 10, 19, 27, 30, 34, 39, 41, 47, 52, 53], 'MM2': [0, 17, 19, 21, 26, 27, 30, 33, 34,
41, 47, 49, 52, 53]}, 0.0009765625: {'I0': [2, 5, 6, 7, 8, 9, 11, 12, 13, 14, 16, 18, 22, 25, 28, 29, 32, 36, 40, 45, 48, 50], 'I': [], 'LL': [1, 17, 19, 20, 21, 43, 53], 'KL': [17, 19, 53], 'V
ar': [], 'MM1': [1, 17, 19, 21, 43, 52], 'MM2': [1, 17, 21, 30, 49, 53]}, 0.000244140625: {'I0': [2, 5, 6, 7, 8, 9, 11, 12, 13, 14, 16, 18, 22, 25, 28, 29, 32, 36, 40, 45, 48, 50], 'I': [], 'L
L': [1, 20, 42], 'KL': [], 'Var': [], 'MM1': [1, 21, 27, 43], 'MM2': [1, 17, 21, 49, 53]}, 6.103515625e-05: {'I0': [2, 5, 6, 7, 8, 9, 11, 12, 13, 14, 16, 18, 22, 25, 28, 29, 32, 36, 40, 45, 48,
50], 'I': [], 'LL': [17, 49], 'KL': [], 'Var': [], 'MM1': [], 'MM2': [17]}, 1.52587890625e-05: {'I0': [2, 5, 6, 7, 8, 9, 11, 12, 13, 14, 16, 18, 22, 25, 28, 29, 32, 36, 40, 45, 48, 50], 'I':
[], 'LL': [], 'KL': [], 'Var': [], 'MM1': [], 'MM2': [21]}}}
```

In [34]:
```python
df_advi = pd.DataFrame(records_advi)
df_advi['rho_rank'] = np.emath.logn(base, df_advi['h'])

I0_advi = df_advi.loc[(df_advi['S']==n_sample[-1]) & (df_advi['T']=="I0")]

kl_advi = df_advi.loc[(df_advi['S']==n_sample[-1]) & (df_advi['T']=="KL")]
var_advi = df_advi.loc[(df_advi['S']==n_sample[-1]) & (df_advi['T']=="Var")]
ll_advi = df_advi.loc[(df_advi['S']==n_sample[-1]) & (df_advi['T']=="LL")]
mm1_advi = df_advi.loc[(df_advi['S']==n_sample[-1]) & (df_advi['T']=="MM1")]
I_advi = df_advi.loc[(df_advi['S']==n_sample[-1]) & (df_advi['T']=="I")]

kl_advi = kl_advi.explode(['khat', 'ndx'])
var_advi = var_advi.explode(['khat', 'ndx'])
ll_advi = ll_advi.explode(['khat', 'ndx'])
I_advi = I_advi.explode(['khat', 'ndx'])
mm1_advi = mm1_advi.explode(['khat', 'ndx'])

original_advi = I_advi[['khat', 'ndx']].drop_duplicates()
```

In [35]:
```python
df_advi_ = df_advi.explode(['khat', "p_psis", 'ndx'])[['ndx', 'p_psis', 'khat', 'T']]
#df_ = df_[df_['p_psis']!=1]
#df_ = df_[df_['p_psis']>1e-5]
df_advi_ = df_advi_[df_advi_['T']!="MM2"]

df_advi_ = df_advi_.groupby(['ndx'])[['khat', 'p_psis']].min()

pd.set_option('display.max_rows', 500)

df_advi_ = df_advi_.reset_index()
df_advi_['y'] = [y_.tolist()[i][0] for i in df_advi_['ndx'].values]
```

In [36]:
```python
fpr_advi, tpr_advi, thresholds_advi = metrics.roc_curve(df_advi_['y'], df_advi_.p_psis, pos_label=1)
precision_advi, recall_advi, thresholds_pr_advi = metrics.precision_recall_curve(df_advi_['y'], df_advi_.p_psis)
```

In [37]:
```python
fig, ax = plt.subplots(1, 2, figsize=(9, 2))
ax[0].plot(fpr, tpr, linewidth=3, color="blue")
ax[0].text(0.5, 0.25, f"MCMC-AUROC: {metrics.auc(fpr, tpr):.3f}", color="blue")

ax[0].plot(fpr_advi, tpr_advi, color="red")
ax[0].text(0.5, 0.1, f"ADVI-AUROC: {metrics.auc(fpr_advi, tpr_advi):.3f}", color="red")

ax[0].set_xlim((0, 1))
ax[0].set_ylim((0, 1))
ax[0].set_title("ROC")

ax[1].plot(recall, precision, linewidth=3, color="blue")
ax[1].plot(recall_advi, precision_advi, color="red")
```

```python
ax[1].text(0.5, 0.8, f"MCMC-AUPRC: {metrics.auc(recall, precision):.3f}", color="blue")
ax[1].text(0.5, 0.65, f"ADVI-AUPRC: {metrics.auc(recall_advi, precision_advi):.3f}", color="red")
ax[1].set_title("Precision-Recall")
ax[1].set_xlim((0, 1))
ax[1].set_ylim((0, 1))

_ = plt.savefig("roccurve.pdf", bbox_inches='tight')
```

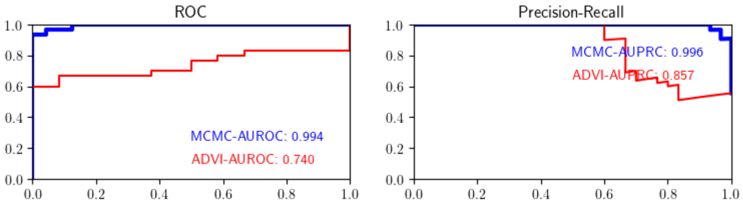

In [38]:
```python
def powerset(iterable):
    "powerset([1,2,3]) --> () (1,) (2,) (3,) (1,2) (1,3) (2,3) (1,2,3)"
    s = list(iterable)  # allows duplicate elements
    return chain.from_iterable(combinations(s, r) for r in range(len(s)+1))
```

In [ ]:
```python
base = 4
h_vals = [base**-r for r in range(11)]

loo_output = []
success = []

for _ in tqdm(range(10)):
    loo_khat = {}
    reduced_ndx = {}
    high_khat_ndx = {}
    n_sample = [1000]
    records = []
    for n_samples in n_sample:
        sample_ndx = np.random.choice(
            range(params["beta0"].shape[0]), size=n_samples, replace=False
        )
        reduced_ndx[n_samples] = {}
        high_khat_ndx[n_samples] = {}
        # print(f"Samples: {n_samples}")
        loo_khat[n_samples] = {}

        params_ = {k: v[sample_ndx] for k, v in params.items()}

        params_["c"] = params_["c"][:, tf.newaxis]
        params_["tau"] = params_["tau"][:, tf.newaxis]
        params_["caux"] = params_["caux"][:, tf.newaxis]
        params_["beta0"] = params_["beta0"][:, tf.newaxis]

        for h in h_vals:
            loo_khat[n_samples][h] = {}
            loo = adaptive_is_loo(lr_model, batch, params_, h, variational=False)
            reduced_ndx[n_samples][h] = {}
            # print(f"rho={h}\n")
            for T in ["I0", "I", "LL", "KL", "Var", "MM1", "MM2"]:
                loo_khat[n_samples][h][T] = np.array(loo[T]["khat"])

                records += [
                    {
                        "h": h,
                        "T": T,
                        "S": n_samples,
                        "khat": loo[T]["khat"],
                        "p_psis": loo[T]["p_psis"].numpy(),
                        "n>0.7": (np.where((loo[T]["khat"] > 0.7))[0]).shape,
                        "ndx": loo[T]["ndx"],
                    }
                ]
                reduced_ndx[n_samples][h][T] = [
                    loo[T]["ndx"][k] for k in np.where((loo[T]["khat"] <= 0.7))[0]
                ]
                # print(reduced_ndx[n_samples][h])

    df = pd.DataFrame(records)
    df["rho_rank"] = np.emath.logn(base, df["h"])
    __df__ = df.explode(["khat", "ndx"])[["ndx", "khat", "T"]]

    counts = {}
    counts["n"] = len(__df__[__df__["T"] != "I0"].ndx.unique())
    for T in transforms:
        ___df__ = __df__.loc[(__df__["T"] == T) & (__df__["khat"] < 0.7)]
        counts[T] = ___df__.groupby("ndx").min().shape[0]
    ___df__ = __df__.loc[
        ((__df__["T"] == "KL") | (__df__["T"] == "Var")) & (__df__["khat"] < 0.7)
    ]
    counts[("KL", "Var")] = ___df__.groupby("ndx").min().shape[0]
    ___df__ = __df__.loc[
        ((__df__["T"] == "MM1") | (__df__["T"] == "MM2") | (__df__["T"] == "LL"))
        & (__df__["khat"] < 0.7)
    ]
    counts[("LL", "MM1", "MM2")] = ___df__.groupby("ndx").min().shape[0]
    ___df__ = __df__.loc[
        ((__df__["T"] == "MM1") | (__df__["T"] == "MM2") | (__df__["T"] == "LL") | (__df__["T"] == "KL") | (__df__["T"] == "Var"))
        & (__df__["khat"] < 0.7)
    ]
    counts[("KL", "Var", "LL", "MM1", "MM2")] = ___df__.groupby("ndx").min().shape[0]
    success += [counts]
```

In [41]:
```python
success = pd.DataFrame(success)
success_ = success.copy()
success_.iloc[:, 1:] = -success_.iloc[:, 1:].sub(success_.iloc[:, 0], axis=1)
```

In [43]:
```python
success.agg(['mean','std'])
```

Out[43]:

	n	KL	Var	LL	MM1	MM2	(KL, Var)	(LL, MM1, MM2)	(KL, Var, LL, MM1, MM2)
**mean**	34.700000	17.900000	15.700000	12.700000	31.600000	32.300000	22.400000	33.900000	34.600000
**std**	2.830391	3.071373	2.406011	2.945807	2.988868	3.497618	3.272783	2.998148	3.025815

In [44]:
```python
(-success.iloc[:, 1:].sub(success.iloc[:, 0], axis=0)).agg(['mean','std'])
```

Out[44]:

	KL	Var	LL	MM1	MM2	(KL, Var)	(LL, MM1, MM2)	(KL, Var, LL, MM1, MM2)
**mean**	16.800000	19.000000	22.000000	3.100000	2.400000	12.300000	0.800000	0.100000
**std**	1.619328	1.825742	1.563472	1.286684	1.646545	2.002776	0.632456	0.316228

In [46]:

```
_df = df.explode(['khat','ndx'])[['rho_rank', 'T', 'ndx', 'khat']]
_df['rho_rank'] *= -1
```

In [47]:

```
base_khat = _df[_df['T']=="I"][['ndx', 'khat']].drop_duplicates().to_dict(orient='records')
base_khat = {r['ndx']: r['khat'] for r in base_khat}
```

In [49]:

```
_df = _df[(_df["T"]!="I") & (_df["T"]!="I0")]
_df[r'$\log(\hat{k}/\hat{k}_0)$'] = _df.apply(lambda x: x.khat/base_khat[x.ndx], axis=1)
_df[r'$-log_4(\rho)$'] = _df['rho_rank']
```

```
/tmp/ipykernel_3246782/2716067429.py:2: SettingWithCopyWarning:
A value is trying to be set on a copy of a slice from a DataFrame.
Try using .loc[row_indexer,col_indexer] = value instead

See the caveats in the documentation: https://pandas.pydata.org/pandas-docs/stable/user_guide/indexing.html#returning-a-view-versus-a-copy
  _df[r'$\log(\hat{k}/\hat{k}_0)$'] = _df.apply(lambda x: x.khat/base_khat[x.ndx], axis=1)
/tmp/ipykernel_3246782/2716067429.py:3: SettingWithCopyWarning:
A value is trying to be set on a copy of a slice from a DataFrame.
Try using .loc[row_indexer,col_indexer] = value instead

See the caveats in the documentation: https://pandas.pydata.org/pandas-docs/stable/user_guide/indexing.html#returning-a-view-versus-a-copy
  _df[r'$-log_4(\rho)$'] = _df['rho_rank']
```

In [50]:

```
_df = _df[_df[r'$\log(\hat{k}/\hat{k}_0)$']<4]
```

In [51]:

```
from plotnine import ggplot, geom_line, geom_point, aes, stat_smooth, facet_wrap, theme, ylim
from plotnine.data import mtcars


plot = (
    ggplot(_df, aes(r'$-log_4(\rho)$', r'$\log(\hat{k}/\hat{k}_0)$', color="T"))
    + geom_line()
    + facet_wrap("ndx", ncol=4)
    + theme(figure_size=(15, 16), legend_position=(.5, .0), legend_direction='horizontal') + ylim((0, 2))

)
plot.show()
plot.save("rho_rank.pdf")
```

```
/home/josh/workspace/readmission/env/lib/python3.10/site-packages/plotnine/geoms/geom_path.py:100: PlotnineWarning: geom_path: Removed 1 rows containing missing values.
```

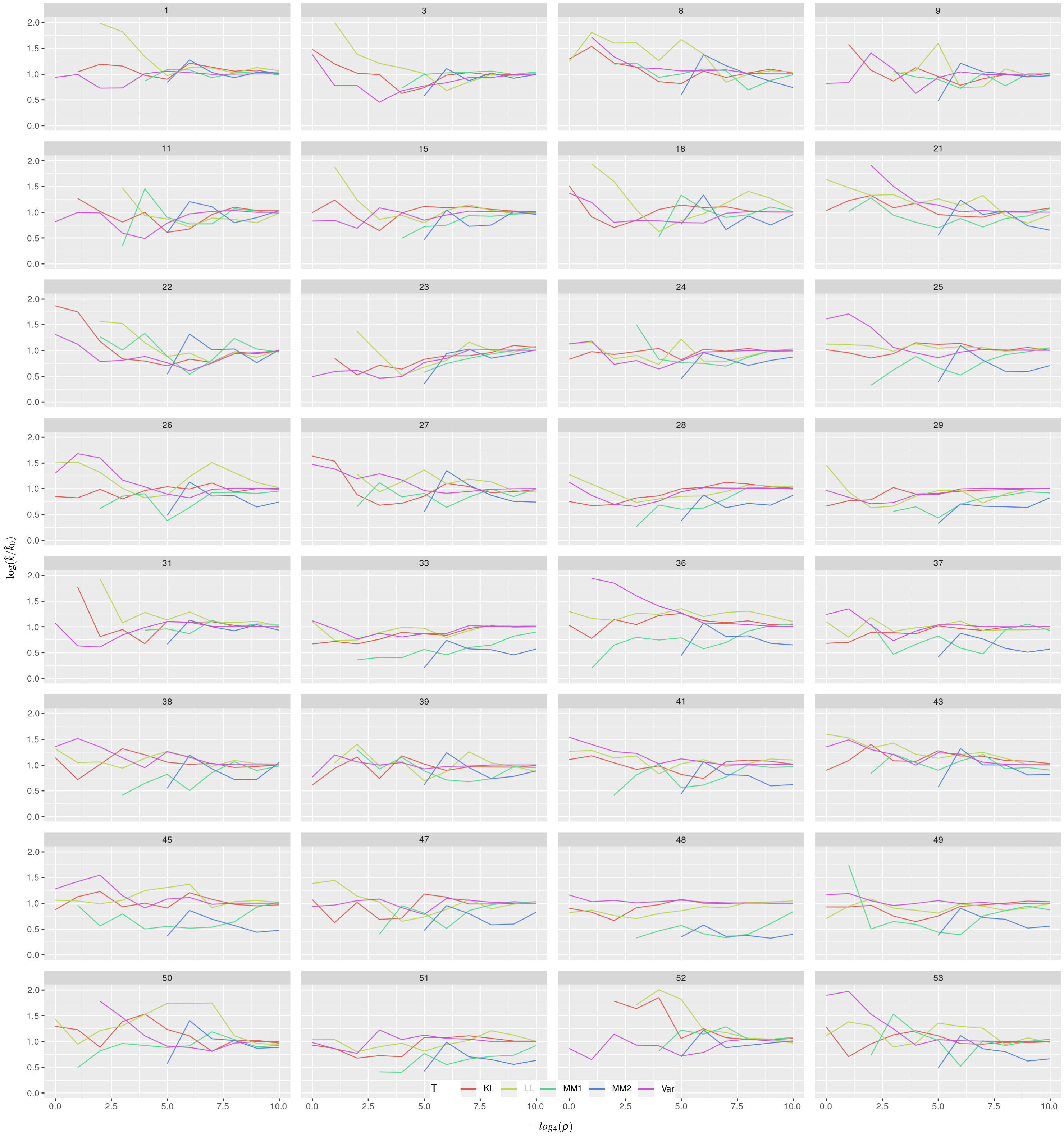


```
/home/josh/workspace/readmission/env/lib/python3.10/site-packages/plotnine/ggplot.py:606: PlotnineWarning: Saving 15 x 16 in image.
/home/josh/workspace/readmission/env/lib/python3.10/site-packages/plotnine/ggplot.py:607: PlotnineWarning: Filename: rho_rank.pdf
/home/josh/workspace/readmission/env/lib/python3.10/site-packages/plotnine/geoms/geom_path.py:100: PlotnineWarning: geom_path: Removed 1 rows containing missing values.
```

S.6 Jupyter notebooks for shallow Relu-net

In the following pages, please find Jupyter notebooks that we used for re-analysis of the data using a shallow Bayesian ReLU-net.

In [1]:
```
%matplotlib inline
from itertools import chain, combinations
import matplotlib
from matplotlib import pyplot as plt
matplotlib.rcParams['pdf.fonttype'] = 42
matplotlib.rcParams['ps.fonttype'] = 42
matplotlib.rcParams['text.usetex'] = True
from abc import ABC
from cmdstanpy import CmdStanModel
import numpy as np
import pandas as pd
from pathlib import Path
from tqdm import tqdm
import nest_asyncio
nest_asyncio.apply()
from collections import defaultdict
from typing import Any
import os

from sklearn import metrics
from matplotlib import pyplot as plt
```

In [2]:
```
import tensorflow as tf
import tensorflow_probability as tfp
import tensorflow_probability.python.bijectors as tfb

# pip install github:https://github.com/mederrata/bayesianquilts
from bayesianquilts.models.logistic_regression_reparam import LogisticRegression2
from bayesianquilts.models.logistic_relunet import ShallowGaussianRelunet
from bayesianquilts.metrics.classification import classification_metrics, auroc, auprc
from bayesianquilts.sampler import psis, nppsis
```

```
2024-10-17 18:21:06.396967: I external/local_tsl/tsl/cuda/cudart_stub.cc:31] Could not find cuda drivers on your machine, GPU will not be used.
2024-10-17 18:21:06.433622: E external/local_xla/xla/stream_executor/cuda/cuda_dnn.cc:9261] Unable to register cuDNN factory: Attempting to register factory for plugin cuDNN when one has alread
y been registered
2024-10-17 18:21:06.433652: E external/local_xla/xla/stream_executor/cuda/cuda_fft.cc:607] Unable to register cuFFT factory: Attempting to register factory for plugin cuFFT when one has already
been registered
2024-10-17 18:21:06.434718: E external/local_xla/xla/stream_executor/cuda/cuda_blas.cc:1515] Unable to register cuBLAS factory: Attempting to register factory for plugin cuBLAS when one has alr
eady been registered
2024-10-17 18:21:06.440048: I external/local_tsl/tsl/cuda/cudart_stub.cc:31] Could not find cuda drivers on your machine, GPU will not be used.
2024-10-17 18:21:06.440646: I tensorflow/core/platform/cpu_feature_guard.cc:182] This TensorFlow binary is optimized to use available CPU instructions in performance-critical operations.
To enable the following instructions: AVX2 FMA, in other operations, rebuild TensorFlow with the appropriate compiler flags.
2024-10-17 18:21:07.474315: W tensorflow/compiler/tf2tensorrt/utils/py_utils.cc:38] TF-TRT Warning: Could not find TensorRT
```

In [3]:
```
X_ = pd.read_csv(Path.home() / "workspace" / "bayesianquilts"/ "bayesianquilts"/ "data" / "overianx.csv", header=None)
y_ = pd.read_table(Path.home() / "workspace" / "bayesianquilts"/ "bayesianquilts"/ "data" / "overiany.csv", header=None)


X_scaled = (X_ - X_.mean())/X_.std()
X_scaled = X_scaled.fillna(0).to_numpy()
y_ = y_.to_numpy()
N = X_scaled.shape[0]
d = X_scaled.shape[1]

print((N, d))
```

```
(54, 1536)
```

In [4]:
```
logistic_relu_horseshoe_code = """
data {
  int<lower=0> N;               // number of observations
  int<lower=0> D_in;            // number of input features
  int<lower=0> D_hidden;        // number of hidden units
  matrix[N, D_in] X;            // input data (N x D_in matrix)
  array[N] int<lower=0, upper=1> y;   // binary target labels (0 or 1)
}

parameters {
  matrix[D_in, D_hidden] w_0;    // weights for the first layer
  vector[D_hidden] b_0;          // biases for the first layer

  vector[D_hidden] w_1;          // weights for the output layer
  real b_1;                      // bias for the output layer
}

transformed parameters {
  vector[N] z_output;           // pre-activation output (logits)
  vector[D_hidden] z_hidden;    // hidden layer (latent outputs)
  for (n in 1:N) {
    // Compute hidden layer activations with ReLU
    for (j in 1:D_hidden) {
      z_hidden[j] = X[n] * w_0[, j] + b_0[j];
      z_hidden[j] = fmax(0, z_hidden[j]);  // ReLU activation
    }

    // Compute the output logits (before applying sigmoid)
    z_output[n] = dot_product(w_1, z_hidden) + b_1;
  }
}

model {
  // Priors on weights and biases (adjust based on your problem)
  to_vector(w_0) ~ normal(0, 1);
  b_0 ~ normal(0, 1);
  w_1 ~ normal(0, 1);
  b_1 ~ normal(0, 1);

  // Likelihood (logistic sigmoid output)
  y ~ bernoulli_logit(z_output);
}


"""

with open("ovarian_relu_model.stan", 'w') as f:
  f.writelines(logistic_relu_horseshoe_code)
```

In [5]:
```
sm = CmdStanModel(stan_file="ovarian_relu_model.stan")
```

```
18:21:12 - cmdstanpy - INFO - compiling stan file /home/josh/workspace/bayesianquilts/ovarian_relu_model.stan to exe file /home/josh/workspace/bayesianquilts/ovarian_relu_model
18:21:27 - cmdstanpy - INFO - compiled model executable: /home/josh/workspace/bayesianquilts/ovarian_relu_model
```

In [6]:
```
d_hiden = 3

# shuffle

# shuffled_ndx = np.random.shuffle(np.arange(N))
```

```
ovarian_data = {
    "N": N,
    "D_in": d,
    "D_hidden": d_hiden,
    "y": y_.astype(int)[:, 0].tolist(),
    "X": X_scaled.tolist()
}

import json

with open("ovarian_relu_data.json", "w") as f:
    json.dump(ovarian_data, f)
```

In [7]:
```
print(d)
```

1536

In [8]:
```
S = 2000
if not os.path.isfile('ovarian_relunet_params.npy'):
    fit = sm.sample(
        data="ovarian_relu_data.json",
        iter_warmup=30000,
        iter_sampling=S,
        thin=2,
        # **control
    )
    print(fit)
    print(fit.diagnose())
```

In [21]:
```
if not os.path.isfile('ovarian_relunet_params.npy'):
    params = fit.stan_variables()
    {k: p.shape for k, p in params.items()}
    params['b_1'] = params['b_1'][..., tf.newaxis]
    params['w_1'] = params['w_1'][..., tf.newaxis]
    np.save('ovarian_relunet_params.npy', params)
else:
    params = np.load('ovarian_relunet_params.npy', allow_pickle=True).tolist()
```

In [22]:
```
nn_model = ShallowGaussianRelunet(
    dim_regressors=d,
    hidden_size=d_hiden
)
```

In [23]:
```
params = {k: tf.cast(v, nn_model.dtype) for k, v in params.items()}
```

In [24]:
```
tfdata = tf.data.Dataset.from_tensor_slices({'X': X_scaled, 'y':y_})
batch_size = N
def data_factory_factory(batch_size=batch_size, repeat=False, shuffle=False):
    def data_factory(batch_size=batch_size):
        if shuffle:
            out = tfdata.shuffle(batch_size*10)
        else:
            out = tfdata

        if repeat:
            out = out.repeat()
        return out.batch(batch_size)
    return data_factory

batch = next(iter(data_factory_factory()()))
```

In [26]:
```
def prediction_fn(data):
    pred = nn_model.predictive_distribution(data, **params)["logits"][..., 1]
    return tf.reduce_mean(pred, axis=0)

bench = classification_metrics(
    data_factory=data_factory_factory(),
    prediction_fn=prediction_fn,
    outcome_label='y',
    by_vars=[]
)

fig, ax = plt.subplots(1, 2, figsize=(9, 2))
ax[0].plot(bench['auroc']['fpr'], bench['auroc']['tpr'])
ax[0].text(0.5, 0.1, f"AUROC: {round(bench['auroc']['auroc'], 2)}")
ax[0].set_xlim((0, 1))
ax[0].set_ylim((0, 1))
ax[0].set_title("ROC")

ax[1].plot(bench['auprc']['recall'], bench['auprc']['precision'])
ax[1].text(0.5, 0.8, f"AUPRC: {round(bench['auprc']['auprc'], 2)}")
ax[1].set_title("Precision-Recall")
ax[1].set_xlim((0, 1))
ax[1].set_ylim((0, 1))
```

```
1it [00:00,  6.77it/s]
```

Out[26]: (0.0, 1.0)

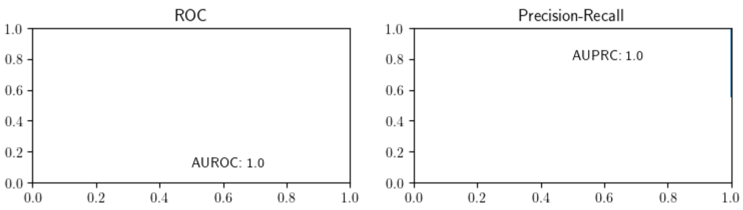

In [120…
```
def entropy(probs):
    return -tf.math.xlogy(probs, probs)


def heaviside(x):
    out = tf.where(x > 0, tf.ones_like(x), tf.zeros_like(x))
    return out


def adaptive_is_loo(
    self: ShallowGaussianRelunet,
    data: dict[str, tf.Tensor],
    params,
    hbar=1.0,
    variational=True,
    approx_jacobians=True,
) -> dict[str, Any]:
```

```
    """_summary_

    Args:
        data (_type_): _description_
        params (_type_): _description_
        hbar (float, optional): _description_. Defaults to 1.0.
        variational (bool, optional):
            Should we trust the variational approximation?
            If False, assumes that one is passing in all the data at once in a single batch.
            Defaults to True.

    Returns:
        _type_: _description_
    """

    # scaled (theta - bar(theta))/Sigma

    _X = tf.cast(data["X"], self.dtype)
    _y = tf.cast(data["y"], self.dtype)[:, 0]
    mu = self.eval(_X, params)[..., 0]
    sigma = tf.math.sigmoid(mu)
    ell = _y * (sigma) + (1 - _y) * (1 - sigma)
    log_ell = tf.math.xlogy(_y, sigma) + tf.math.xlogy(1 - _y, 1 - sigma)
    log_ell_prime = _y * (1 - sigma) - (1 - _y) * sigma
    log_ell_doubleprime = -sigma * (1 - sigma)
    _, khat0 = nppsis.psislw(-log_ell)

    """
    sigma.shape is samples x datapoints
    """

    ndx_to_transform = np.where(khat0 > 0.7)[0]
    ndx_to_leave = np.where((khat0 <= 0.7))[0]

    def IS(T, X, y):
        mu = self.eval(X, params)[..., 0]
        sigma = tf.math.sigmoid(mu)
        ell = y * (sigma) + (1 - y) * (1 - sigma)
        log_ell = tf.math.xlogy(y, sigma) + tf.math.xlogy(1 - y, 1 - sigma)
        log_ell_prime = y * (1 - sigma) - (1 - y) * sigma
        log_ell_doubleprime = -sigma * (1 - sigma)
        # compute # \nabla\log\pi(\btheta|\calD)
        if variational:
            # \nabla\log\pi = -\Sigma^{-1}(theta - \bar{\theta})
            grad_log_pi = {
                "b_0": -(
                    params["b_0"] - self.surrogate_distribution.model["b_0"].mean()
                )
                / self.surrogate_distribution.model["b_0"].variance(),
                "b_1": -(
                    params["b_1"] - self.surrogate_distribution.model["b_1"].mean()
                )
                / self.surrogate_distribution.model["b_1"].variance(),
                "w_0": -(
                    params["w_0"] - self.surrogate_distribution.model["w_0"].mean()
                )
                / self.surrogate_distribution.model["w_0"].variance(),
                "w_1": -(
                    params["w_1"] - self.surrogate_distribution.model["w_1"].mean()
                )
                / self.surrogate_distribution.model["w_1"].variance(),
            }
            b0_sd = self.surrogate_distribution.model["b_0"].variance() ** 0.5
            b1_sd = self.surrogate_distribution.model["b_1"].variance() ** 0.5
            w0_sd = self.surrogate_distribution.model["w_0"].variance() ** 0.5
            w1_sd = self.surrogate_distribution.model["w_1"].variance() ** 0.5
            log_pi = (
                self.surrogate_distribution.model["b_0"].log_prob(params["b_0"])
                + self.surrogate_distribution.model["b_1"].log_prob(params["b_1"])
                + self.surrogate_distribution.model["w_1"].log_prob(params["w_1"])
                + self.surrogate_distribution.model["w_0"].log_prob(params["w_0"])
            )
            log_pi -= tf.reduce_max(log_pi, axis=0)
            # log_pi.shape: [samples]
        else:
            """
            Recall Bayes rule:
            \log pi(\btheta|\calD) = \sum_i\log ell_i(\btheta) + \log\pi(\btheta) + const

            so
            \nabla\log\pi(\btheta|\calD) = \sum_i (ell_i)'x + grad\log\pi(\btheta)

            """
            log_prior = self.prior_distribution.log_prob(params)

            log_pi = tf.reduce_sum(log_ell, axis=1, keepdims=True)[:, 0]
            z1 = tf.nn.relu(tf.matmul(X, params["w_0"]))
            # pi \propto #@TODO

            grad_log_pi = {
                "b_0": (
                    tf.reduce_sum(
                        (
                            params["w_1"][..., tf.newaxis, :, :]
                            * log_ell_prime[..., tf.newaxis, tf.newaxis]
                        )
                        * heaviside(z1)[..., tf.newaxis],
                        axis=1,
                        keepdims=False,
                    )[..., 0]
                    - 0.5 * params["b_0"] ** 2
                ),
                "b_1": tf.reduce_sum(
                    log_ell_prime[..., tf.newaxis], axis=1, keepdims=False
                )
                - 0.5 * params["b_1"] ** 2,
                "w_0": (
                    tf.reduce_sum(
                        (
                            (
                                params["w_1"][..., tf.newaxis, :, :]
                                * log_ell_prime[..., tf.newaxis, tf.newaxis]
                                * heaviside(z1)[..., tf.newaxis]
                            )[..., tf.newaxis, :, 0]
                            * X[..., tf.newaxis]
                        ),
                        axis=1,
                        keepdims=False,
                    )  # want S x N x k
```

```
                - 0.5 * params["w_0"] ** 2
            ),
            "w_1": tf.reduce_sum(
                log_ell_prime[..., tf.newaxis, tf.newaxis] * z1[..., tf.newaxis],
                axis=1,
                keepdims=False,
            )
            - 0.5 * params["w_1"] ** 2,
        }

        grad_log_prior = {
            "b_0": -0.5 * params["b_0"] ** 2
            - 0.5 * tf.cast(tf.math.sqrt(2 * np.pi), self.dtype),
            "b_1": -0.5 * params["b_1"] ** 2
            - 0.5 * tf.cast(tf.math.sqrt(2 * np.pi), self.dtype),
            "w_0": -0.5 * params["w_0"] ** 2
            - 0.5 * tf.cast(tf.math.sqrt(2 * np.pi), self.dtype),
            "w_1": -0.5 * params["w_1"] ** 2
            - 0.5 * tf.cast(tf.math.sqrt(2 * np.pi), self.dtype),
        }
        # grad_log_pi += grad_log_prior[:, tf.newaxis, :]

        b0_sd = tf.math.reduce_std(params["b_0"], 0, keepdims=True)
        b1_sd = tf.math.reduce_std(params["b_1"], 0, keepdims=True)
        w0_sd = tf.math.reduce_std(params["w_0"], 0, keepdims=True)
        w1_sd = tf.math.reduce_std(params["w_1"], 0, keepdims=True)

    b0_adj, b1_adj, w0_adj, w1_adj, logJ = T(
        X,
        y,
        log_pi=log_pi,
        grad_log_pi=grad_log_pi,
        b0_sd=b0_sd,
        b1_sd=b1_sd,
        w0_sd=w0_sd,
        w1_sd=w1_sd,
    )
    transformed = {"b_0": b0_adj, "b_1": b1_adj, "w_0": w0_adj, "w_1": w1_adj}
    mu_new = self.nn.eval(X[:, tf.newaxis, :], [w0_adj, b0_adj, w1_adj, b1_adj])[
        ..., 0, 0
    ]
    sigma_new = tf.math.sigmoid(mu_new)
    ell_new = y * (sigma_new) + (1 - y) * (1 - sigma_new)
    log_ell_new = tf.math.xlogy(y, sigma_new) + tf.math.xlogy(1 - y, 1 - sigma_new)

    if variational:
        # We trust the variational approximation, so \hat{pi} = pi
        # N_samples x N_data
        delta_log_pi = (
            self.surrogate_distribution.log_prob(transformed)
            - log_pi[:, tf.newaxis]
        )
        delta_log_pi = delta_log_pi - tf.reduce_max(
            delta_log_pi, axis=0, keepdims=True
        )

    else:
        # we don't trust the variational approximation
        # Need to compute log_pi directly by summing over the likelihood

        ell_cross = tf.math.sigmoid(
            self.nn.eval(X, [w0_adj, b0_adj, w1_adj, b1_adj])[..., 0]
        )
        ell_cross = tf.math.xlogy(y, ell_cross) + tf.math.xlogy(
            1 - y, 1 - ell_cross
        )
        ell_cross = tf.math.reduce_sum(ell_cross, axis=-1)

        log_prior_new = self.prior_distribution.log_prob(transformed)

        log_pi_new = ell_cross
        delta_log_prior = log_prior_new - log_prior[:, tf.newaxis]
        # Incorporate the prior
        delta_log_pi = log_pi_new - log_pi[:, tf.newaxis] + delta_log_prior

    # regularize delta_log_pi

    def regularize(tensor):
        min_finite = tf.reduce_min(
            tf.where(
                tf.math.is_finite(tensor),
                tensor,
                tf.zeros_like(tensor),
            )
        )
        return tf.where(
            tf.math.is_finite(tensor),
            tensor,
            min_finite * tf.ones_like(tensor),
        )

    delta_log_pi = regularize(delta_log_pi)
    delta_log_pi -= tf.reduce_max(delta_log_pi, axis=0)
    log_eta_weights = delta_log_pi - regularize(log_ell_new) + logJ
    log_eta_weights = log_eta_weights - tf.reduce_max(log_eta_weights, axis=0)
    psis_weights, khat = nppsis.psislw(log_eta_weights)

    eta_weights = tf.math.exp(log_eta_weights)
    eta_weights = eta_weights / tf.reduce_sum(eta_weights, axis=0, keepdims=True)

    psis_weights = tf.math.exp(psis_weights)
    psis_weights = psis_weights / tf.math.reduce_sum(
        psis_weights, axis=0, keepdims=True
    )

    weight_entropy = entropy(eta_weights)
    psis_entropy = entropy(psis_weights)

    p_loo_new = tf.reduce_sum(sigma_new * eta_weights, axis=0)
    p_loo_psis = tf.reduce_sum(sigma_new * psis_weights, axis=0)
    p_loo_sd = tf.math.reduce_std(sigma_new * eta_weights, axis=0)
    ll_loo_new = tf.reduce_sum(eta_weights * ell_new, axis=0)
    ll_loo_psis = tf.reduce_sum(psis_weights * ell_new, axis=0)
    ll_loo_sd = tf.math.reduce_std(eta_weights * ell_new, axis=0)
    out = (
        eta_weights,
        psis_weights,
        p_loo_new,
        p_loo_sd,
```

```
            ll_loo_new,
            ll_loo_sd,
            weight_entropy,
            khat,
            p_loo_psis,
            ll_loo_psis,
        )
        return out

    def T_kl(X, y, log_pi, grad_log_pi, b0_sd, b1_sd, w0_sd, w1_sd, **kwargs):
        log_pi_ = log_pi - tf.reduce_max(log_pi, axis=0, keepdims=True)
        mu = self.eval(X, params)[..., 0]
        sigma = tf.math.sigmoid(mu)
        pre = ((-1) ** y * tf.math.exp(log_pi_[..., tf.newaxis] + mu * (1 - 2 * y)))[
            ..., tf.newaxis
        ]
        z1 = tf.nn.relu(tf.matmul(X, params["w_0"]))
        Q_b1 = pre
        Q_w1 = (pre * z1)[..., tf.newaxis]
        Q_b0 = (
            pre[..., tf.newaxis]
            * heaviside(z1)[..., tf.newaxis]
            * params["w_1"][..., tf.newaxis, :, :]
        )
        Q_w0 = (
            pre[..., tf.newaxis, :]
            * heaviside(z1)[..., tf.newaxis, :]
            * X[..., tf.newaxis]
            * params["w_1"][..., tf.newaxis, tf.newaxis, :, 0]
        )

        standardized = tf.concat(
            [
                tf.reduce_max(tf.math.abs(Q_b0 / tf.transpose(b0_sd)), axis=-1),
                tf.math.abs(Q_b1 / b1_sd),
                tf.reduce_max(tf.math.abs(Q_w0 / w0_sd), axis=-1),
                (Q_w1 / w1_sd)[..., 0],
            ],
            axis=-1,
        )
        standardized = tf.reduce_max(tf.math.abs(standardized), axis=-1)
        standardized = tf.reduce_max(standardized, axis=0, keepdims=True)[
            ..., tf.newaxis
        ]

        h = hbar / standardized

        b0_adj = params["b_0"][..., tf.newaxis, :] + h * Q_b0[..., 0]
        b1_adj = params["b_1"][..., tf.newaxis, :] + h * Q_b1
        w0_adj = params["w_0"][..., tf.newaxis, :, :] + h[..., tf.newaxis] * Q_w0
        w1_adj = params["w_1"][..., tf.newaxis, :, :] + h[..., tf.newaxis] * Q_w1

        eigen_plus = tf.reduce_sum(
            heaviside(z1)[..., tf.newaxis] * X[:, tf.newaxis] ** 2, axis=-1
        )
        eigen_plus = tf.math.sqrt(eigen_plus)

        u_k = heaviside(z1)[..., tf.newaxis] * X[:, tf.newaxis]
        u_k = tf.math.sqrt(tf.math.reduce_sum(u_k**2, axis=-1))  # norm |u_k|

        #
        Jpre = (-1) ** y[tf.newaxis, :] * tf.math.exp(
            log_pi_[..., tf.newaxis] + mu * (1 - 2 * y[tf.newaxis, :])
        )

        # assumeble the Jacobian approximation
        # |J| = | 1 + h\nabla\cdot Q|

        dQ_b0 = (grad_log_pi["b_0"] * tf.math.exp(log_pi_)[:, tf.newaxis])[
            :, tf.newaxis, :, tf.newaxis
        ] + (1 - 2 * y[ tf.newaxis, :, tf.newaxis, tf.newaxis]) * (
            heaviside(z1)[..., tf.newaxis] * params["w_1"][..., tf.newaxis, :, :]
        )

        dQ_w0 = (
            grad_log_pi["w_0"][..., tf.newaxis, :, :]
            * tf.math.exp(log_pi_)[:, tf.newaxis, tf.newaxis, tf.newaxis]
            + (1 - 2 * y[tf.newaxis, :, tf.newaxis, tf.newaxis])
            * heaviside(z1)[..., tf.newaxis, :]
            * X[tf.newaxis, :, :, tf.newaxis]
            * params["w_1"][..., 0][..., tf.newaxis, tf.newaxis, :]
        )

        dQ_b1 = (grad_log_pi["b_1"] * tf.math.exp(log_pi_)[:, tf.newaxis])[
            :, tf.newaxis, :
        ] + (1 - 2 * y[tf.newaxis, :, tf.newaxis])

        dQ_w1 = (
            grad_log_pi["w_1"][..., tf.newaxis, :, :]
            * tf.math.exp(log_pi_)[:, tf.newaxis, tf.newaxis, tf.newaxis]
            + (1 - 2 * y[tf.newaxis, :, tf.newaxis, tf.newaxis])
            * z1[..., tf.newaxis]
        )

        dQ_b0 = tf.reduce_sum(h[..., tf.newaxis]*dQ_b0*Q_b0, axis=[-1, -2])
        dQ_b1 = tf.reduce_sum(h*dQ_b1*Q_b1, axis=[-1])
        dQ_w0 = tf.reduce_sum(h[..., tf.newaxis]*dQ_w0*Q_w0, axis=[-1, -2])
        dQ_w1 = tf.reduce_sum(h[..., tf.newaxis]*dQ_w1*Q_w1, axis=[-1, -2])

        logJ = tf.math.log1p(dQ_b0 + dQ_b1 + dQ_w0 + dQ_w1)
        return b0_adj, b1_adj, w0_adj, w1_adj, logJ

    def T_I(X, y, **kwargs):
        mu = self.eval(X, params)[..., 0]
        sigma = tf.math.sigmoid(mu)

        log_ell = tf.math.xlogy(y, sigma) + tf.math.xlogy(1 - y, 1 - sigma)
        Q = tf.zeros_like(log_ell)
        out = (
            params["b_0"][..., tf.newaxis, :] + Q[..., tf.newaxis],
            params["b_1"][..., tf.newaxis, :] + Q[..., tf.newaxis],
            params["w_0"][..., tf.newaxis, :, :] + Q[..., tf.newaxis, tf.newaxis],
            params["w_1"][..., tf.newaxis, :, :] + Q[..., tf.newaxis, tf.newaxis],
            tf.zeros_like(Q),
        )
        return out

    def T_var(X, y, log_pi, grad_log_pi, b0_sd, b1_sd, w0_sd, w1_sd, **kwargs):
        log_pi_ = log_pi - tf.reduce_max(log_pi, axis=0, keepdims=True)
```

```
        mu = self.eval(X, params)[..., 0]
        sigma = tf.math.sigmoid(mu)
        pre = ((-1) ** y * tf.math.exp(log_pi_[..., tf.newaxis] + 2* mu * (1 - 2 * y)))[
            ..., tf.newaxis
        ]
        z1 = tf.nn.relu(tf.matmul(X, params["w_0"]))
        Q_b1 = pre
        Q_w1 = (pre * z1)[..., tf.newaxis]
        Q_b0 = (
            pre[..., tf.newaxis]
            * heaviside(z1)[..., tf.newaxis]
            * params["w_1"][..., tf.newaxis, :, :]
        )
        Q_w0 = (
            pre[..., tf.newaxis, :]
            * heaviside(z1)[..., tf.newaxis, :]
            * X[..., tf.newaxis]
            * params["w_1"][..., tf.newaxis, tf.newaxis, :, 0]
        )

        standardized = tf.concat(
            [
                tf.reduce_max(tf.math.abs(Q_b0 / tf.transpose(b0_sd)), axis=-1),
                tf.math.abs(Q_b1 / b1_sd),
                tf.reduce_max(tf.math.abs(Q_w0 / w0_sd), axis=-1),
                (Q_w1 / w1_sd)[..., 0],
            ],
            axis=-1,
        )
        standardized = tf.reduce_max(tf.math.abs(standardized), axis=-1)
        standardized = tf.reduce_max(standardized, axis=0, keepdims=True)[
            ..., tf.newaxis
        ]

        h = hbar / standardized

        b0_adj = params["b_0"][..., tf.newaxis, :] + h * Q_b0[..., 0]
        b1_adj = params["b_1"][..., tf.newaxis, :] + h * Q_b1
        w0_adj = params["w_0"][..., tf.newaxis, :, :] + h[..., tf.newaxis] * Q_w0
        w1_adj = params["w_1"][..., tf.newaxis, :, :] + h[..., tf.newaxis] * Q_w1

        eigen_plus = tf.reduce_sum(
            heaviside(z1)[..., tf.newaxis] * X[:, tf.newaxis] ** 2, axis=-1
        )
        eigen_plus = tf.math.sqrt(eigen_plus)

        u_k = heaviside(z1)[..., tf.newaxis] * X[:, tf.newaxis]
        u_k = tf.math.sqrt(tf.math.reduce_sum(u_k**2, axis=-1))  # norm |u_k|

        #
        Jpre = (-1) ** y[tf.newaxis, :] * tf.math.exp(
            log_pi_[..., tf.newaxis] + mu * (1 - 2 * y[tf.newaxis, :])
        )

        # assumeble the Jacobian approximation
        # |J| = | 1 + h\nabla\cdot Q|

        dQ_b0 = (grad_log_pi["b_0"] * tf.math.exp(log_pi_)[:, tf.newaxis])[
            :, tf.newaxis, :, tf.newaxis
        ] + 2*(1 - 2 * y[ tf.newaxis, :, tf.newaxis, tf.newaxis]) * (
            heaviside(z1)[..., tf.newaxis] * params["w_1"][..., tf.newaxis, :, :]
        )

        dQ_w0 = (
            grad_log_pi["w_0"][..., tf.newaxis, :, :]
            * tf.math.exp(log_pi_)[:, tf.newaxis, tf.newaxis, tf.newaxis]
            + 2*(1 - 2 * y[tf.newaxis, :, tf.newaxis, tf.newaxis])
            * heaviside(z1)[..., tf.newaxis, :]
            * X[tf.newaxis, :, :, tf.newaxis]
            * params["w_1"][..., 0][..., tf.newaxis, tf.newaxis, :]
        )

        dQ_b1 = (grad_log_pi["b_1"] * tf.math.exp(log_pi_)[:, tf.newaxis])[
            :, tf.newaxis, :
        ] + 2*(1 - 2 * y[tf.newaxis, :, tf.newaxis])

        dQ_w1 = (
            grad_log_pi["w_1"][..., tf.newaxis, :, :]
            * tf.math.exp(log_pi_)[:, tf.newaxis, tf.newaxis, tf.newaxis]
            + 2*(1 - 2 * y[tf.newaxis, :, tf.newaxis, tf.newaxis])
            * z1[..., tf.newaxis]
        )

        dQ_b0 = tf.reduce_sum(h[..., tf.newaxis]*dQ_b0*Q_b0, axis=[-1, -2])
        dQ_b1 = tf.reduce_sum(h*dQ_b1*Q_b1, axis=[-1])
        dQ_w0 = tf.reduce_sum(h[..., tf.newaxis]*dQ_w0*Q_w0, axis=[-1, -2])
        dQ_w1 = tf.reduce_sum(h[..., tf.newaxis]*dQ_w1*Q_w1, axis=[-1, -2])

        logJ = tf.math.log1p(dQ_b0 + dQ_b1 + dQ_w0 + dQ_w1)
        return b0_adj, b1_adj, w0_adj, w1_adj, logJ

    def T_MM1(X, y, **kwargs):
        # need to transform b_0, b_1, w_0, w_1
        mu = self.eval(X, params)[..., 0]
        sigma = tf.math.sigmoid(mu)
        ell = y * (sigma) + (1 - y) * (1 - sigma)  # S x N

        weights = (
            1
            / ell[..., tf.newaxis]
            / tf.reduce_sum(1 / ell[..., tf.newaxis], axis=0, keepdims=True)
        )

        def theta_bar(param, weights):  # param is S x K
            hat = tf.reduce_mean(param[:, tf.newaxis, ...], axis=0, keepdims=True)
            if len(param.shape) > 2:
                weights = weights[..., tf.newaxis]
            hat_w = param[:, tf.newaxis, ...] * weights
            hat_w = tf.reduce_sum(hat_w, axis=0, keepdims=True)
            v = tf.reduce_mean((param[:, tf.newaxis, ...] - hat) ** 2)
            v_w = tf.reduce_sum(
                weights * (param[:, tf.newaxis, ...] - hat) ** 2, axis=0, keepdims=True
            )
            return hat, hat_w, v, v_w

        bar_b0, bar_b0_w, b0_v, b0_v_w = theta_bar(params["b_0"], weights)
        bar_b1, bar_b1_w, b1_v, b1_v_w = theta_bar(params["b_1"], weights)

        bar_w0, bar_w0_w, w0_v, w0_v_w = theta_bar(params["w_0"], weights)
```

```
        bar_w1, bar_w1_w, w1_v, w1_v_w = theta_bar(params["w_1"], weights)

        # tf.math.exp(0.5*(tf.math.log(beta_v_w) - tf.math.log(beta_v)))-tf.math.sqrt(beta_v_w / beta_v)

        b0_adj = params["b_0"][:, tf.newaxis, ...] + hbar * (-bar_b0 + bar_b0_w)
        b1_adj = params["b_1"][:, tf.newaxis, ...] + hbar * (-bar_b1 + bar_b1_w)
        w0_adj = params["w_0"][:, tf.newaxis, ...] + hbar * (-bar_w0 + bar_w0_w)
        w1_adj = params["w_1"][:, tf.newaxis, ...] + hbar * (-bar_w1 + bar_w1_w)

        return b0_adj, b1_adj, w0_adj, w1_adj, tf.zeros_like(ell)

    def T_MM2(X, y, **kwargs):

        mu = self.eval(X, params)[..., 0]
        sigma = tf.math.sigmoid(mu)
        ell = y * (sigma) + (1 - y) * (1 - sigma)  # S x N

        weights = (
            1
            / ell[..., tf.newaxis]
            / tf.reduce_sum(1 / ell[..., tf.newaxis], axis=0, keepdims=True)
        )

        def theta_bar(param, weights):  # param is S x K
            hat = tf.reduce_mean(param[:, tf.newaxis, ...], axis=0, keepdims=True)
            if len(param.shape) > 2:
                weights = weights[..., tf.newaxis]
            hat_w = param[:, tf.newaxis, ...] * weights
            hat_w = tf.reduce_sum(hat_w, axis=0, keepdims=True)
            v = tf.reduce_mean((param[:, tf.newaxis, ...] - hat) ** 2)
            v_w = tf.reduce_sum(
                weights * (param[:, tf.newaxis, ...] - hat) ** 2, axis=0, keepdims=True
            )
            return hat, hat_w, v, v_w

        bar_b0, bar_b0_w, b0_v, b0_v_w = theta_bar(params["b_0"], weights)
        bar_b1, bar_b1_w, b1_v, b1_v_w = theta_bar(params["b_1"], weights)

        bar_w0, bar_w0_w, w0_v, w0_v_w = theta_bar(params["w_0"], weights)
        bar_w1, bar_w1_w, w1_v, w1_v_w = theta_bar(params["w_1"], weights)

        # tf.math.exp(0.5*(tf.math.log(beta_v_w) - tf.math.log(beta_v)))-tf.math.sqrt(beta_v_w / beta_v)

        b0_adj = params["b_0"][:, tf.newaxis, ...] + hbar * (
            (tf.math.sqrt(b0_v_w / b0_v) - 1) * params["b_0"][:, tf.newaxis, ...]
            - tf.math.sqrt(b0_v_w / b0_v) * bar_b0
            + bar_b0_w
        )
        b1_adj = params["b_1"][:, tf.newaxis, ...] + hbar * (
            (tf.math.sqrt(b1_v_w / b1_v) - 1) * params["b_1"][:, tf.newaxis, ...]
            - tf.math.sqrt(b1_v_w / b1_v) * bar_b1
            + bar_b0_w
        )
        w0_adj = params["w_0"][:, tf.newaxis, ...] + hbar * (
            (tf.math.sqrt(w0_v_w / w0_v) - 1) * params["w_0"][:, tf.newaxis, ...]
            - tf.math.sqrt(w0_v_w / w0_v) * bar_w0
            + bar_w0_w
        )
        w1_adj = params["w_1"][:, tf.newaxis, ...] + hbar * (
            (tf.math.sqrt(w1_v_w / w1_v) - 1) * params["w_1"][:, tf.newaxis, ...]
            - tf.math.sqrt(w1_v_w / w1_v) * bar_w1
            + bar_w1_w
        )

        # piece together the Jacobian
        # every component gets multiplied by something like (sqrt(beta0_v_w/beta0_v)-1)

        J_b0 = tf.math.sqrt(b0_v_w / b0_v) - 1
        J_b1 = tf.math.sqrt(b1_v_w / b1_v) - 1
        J_w0 = tf.math.sqrt(w0_v_w / w0_v) - 1
        J_w1 = tf.math.sqrt(w1_v_w / w1_v) - 1

        J_b0 = tf.reduce_sum(tf.math.log1p(hbar * J_b0), axis=-1)
        J_b1 = tf.reduce_sum(tf.math.log1p(hbar * J_b1), axis=-1)
        J_w0 = tf.reduce_sum(tf.math.log1p(hbar * J_w0), axis=[-1, -2])
        J_w1 = tf.reduce_sum(tf.math.log1p(hbar * J_w1), axis=[-1, -2])
        # shift by mean
        return (
            b0_adj,
            b1_adj,
            w0_adj,
            w1_adj,
            tf.zeros_like(ell) + J_b0 + J_b1 + J_w0 + J_w1,
        )

    X_ = tf.gather(_X, ndx_to_leave)
    y_ = tf.gather(_y, ndx_to_leave)
    (
        eta_I,
        eta_I_psis,
        p_loo_I,
        p_loo_I_sd,
        ll_loo_I,
        ll_loo_I_sd,
        S_I,
        k_I,
        p_psis_I,
        ll_psis_I,
    ) = IS(T_I, X_, y_)

    out = {
        "I0": {
            "p_loo": p_loo_I,
            "p_loo_sd": p_loo_I_sd,
            "ll_loo": ll_loo_I,
            "ll_loo_sd": ll_loo_I_sd,
            "S": S_I,
            "khat": k_I,
            "p_psis": p_psis_I,
            "ll_psis": ll_psis_I,
            "ndx": ndx_to_leave,
        }
    }

    transforms = {
        "MM1": T_MM1,
        "MM2": T_MM2,
        "I": T_I,
        "KL": T_kl,
```

```
        "Var": T_var,
        # "LL": T_ll,
    }

    X_ = tf.gather(_X, ndx_to_transform)
    y_ = tf.gather(_y, ndx_to_transform)
    for lab, fun in transforms.items():
        (
            eta_,
            eta_psis_,
            p_loo_,
            p_loo_sd_,
            ll_loo_,
            ll_loo_s_d,
            S_,
            k_,
            p_psis_,
            ll_psis_,
        ) = IS(fun, X_, y_)

        out = {
            **out,
            lab: {
                "p_loo": p_loo_,
                "p_loo_sd": p_loo_sd_,
                "ll_loo": ll_loo_,
                "ll_loo_sd": ll_loo_s_d,
                "S": S_,
                "khat": k_,
                "p_psis": p_psis_,
                "ll_psis": ll_psis_,
                "ndx": ndx_to_transform,
            },
        }

    return {**out, "ndx_transformed": ndx_to_transform}


nparams = 64
loo = adaptive_is_loo(
    nn_model,
    batch,
    {k: v[:nparams, ...] for k, v in params.items()},
    1e-5,
    variational=False,
)

# loo = adaptive_is_loo(lr_model, test_batch, param_test, 0.01, variational=False)


for T in ["I", "KL", "Var", "MM1", "MM2"]:
    print(
        f"{T}: {np.sqrt(np.sum(loo[T]['p_loo_sd']**2))} entropy: {np.sqrt(np.sum(loo[T]['S']))} khat>0.7: {np.sum(loo[T]['khat']>0.7)}"
    )
```

```
I: 0.03684039339225807 entropy: 13.169230304298633 khat>0.7: 43
KL: 0.03684639439575884 entropy: 13.16906485482308 khat>0.7: 43
Var: 0.03685327183311002 entropy: 13.168805732146133 khat>0.7: 43
MM1: 0.03666070813045722 entropy: 13.171922678738264 khat>0.7: 43
MM2: 0.057355294557717776 entropy: 12.838782417377582 khat>0.7: 13
```

In [122…

```
base = 4
h_vals = [base**-r for r in range(-3, 7)]

loo_output = []

for _ in tqdm(range(1)):
    loo_khat = {}
    reduced_ndx = {}
    high_khat_ndx = {}
    n_sample = [1000]
    records = []
    for n_samples in n_sample:
        sample_ndx = np.random.choice(range(params['w_0'].shape[0]), size=n_samples, replace=False)
        reduced_ndx[n_samples] = {}
        high_khat_ndx[n_samples] = {}
        # print(f"Samples: {n_samples}")
        loo_khat[n_samples] = {}
        params_ = {k: v.numpy()[sample_ndx] for k, v in params.items()}
        params_ = {k: tf.cast(v, nn_model.dtype) for k, v in params_.items()}

        for h in h_vals:
            loo_khat[n_samples][h] = {}
            loo = adaptive_is_loo(nn_model, batch, params_, h, variational=False)
            reduced_ndx[n_samples][h] = {}
            # print(f"rho={h}\n")
            for T in ["I0", "I", "KL", "Var", "MM1", "MM2"]:
                loo_khat[n_samples][h][T] = np.array(loo[T]["khat"])

                records += [
                    {
                        "h": h,
                        "T": T,
                        "S": n_samples,
                        "khat": loo[T]["khat"],
                        "p_psis": loo[T]["p_psis"].numpy(),
                        "n>0.7": (np.where((loo[T]["khat"] > 0.7))[0]).shape,
                        "ndx": loo[T]["ndx"]
                    }
                ]
                reduced_ndx[n_samples][h][T] = [loo[T]["ndx"][k] for k in np.where((loo[T]["khat"] <= 0.7))[0]]
                # print(reduced_ndx[n_samples][h])


print(reduced_ndx)
```

```
100%|██████████| 1/1 [02:09<00:00, 129.01s/it]
```

```
{1000: {64: {'I0': [0, 1, 2, 3, 4, 5, 9, 10, 13, 17, 20, 22, 23, 24, 25, 28, 29, 30, 31, 34, 35, 37, 38, 39, 40, 41, 42, 43, 46, 47, 48, 49, 51, 52], 'I': [], 'KL': [6, 8, 11, 14, 16, 19, 26, 3
2, 44, 45, 50, 53], 'Var': [32], 'MM1': [], 'MM2': []}, 16: {'I0': [0, 1, 2, 3, 4, 5, 9, 10, 13, 17, 20, 22, 23, 24, 25, 28, 29, 30, 31, 34, 35, 37, 38, 39, 40, 41, 42, 43, 46, 47, 48, 49, 51,
52], 'I': [], 'KL': [21, 26, 27, 32, 44, 50], 'Var': [27], 'MM1': [], 'MM2': []}, 4: {'I0': [0, 1, 2, 3, 4, 5, 9, 10, 13, 17, 20, 22, 23, 24, 25, 28, 29, 30, 31, 34, 35, 37, 38, 39, 40, 41, 42,
43, 46, 47, 48, 49, 51, 52], 'I': [], 'KL': [26, 32, 44, 50], 'Var': [], 'MM1': [50], 'MM2': []}, 1: {'I0': [0, 1, 2, 3, 4, 5, 9, 10, 13, 17, 20, 22, 23, 24, 25, 28, 29, 30, 31, 34, 35, 37, 38,
39, 40, 41, 42, 43, 46, 47, 48, 49, 51, 52], 'I': [], 'KL': [26, 50, 53], 'Var': [18], 'MM1': [6, 7, 19, 26, 27, 33, 36, 45, 50, 53], 'MM2': [7, 19, 27]}, 0.25: {'I0': [0, 1, 2, 3, 4, 5, 9, 10,
13, 17, 20, 22, 23, 24, 25, 28, 29, 30, 31, 34, 35, 37, 38, 39, 40, 41, 42, 43, 46, 47, 48, 49, 51, 52], 'I': [], 'KL': [26, 27], 'Var': [], 'MM1': [6, 7, 11, 15, 16, 19, 21, 26, 27, 33, 36, 4
5, 50, 53], 'MM2': [6, 7, 11, 15, 16, 19, 21, 26, 27, 33, 36, 45, 50, 53]}, 0.0625: {'I0': [0, 1, 2, 3, 4, 5, 9, 10, 13, 17, 20, 22, 23, 24, 25, 28, 29, 30, 31, 34, 35, 37, 38, 39, 40, 41, 42,
43, 46, 47, 48, 49, 51, 52], 'I': [], 'KL': [26, 27, 33, 36, 45], 'Var': [], 'MM1': [11, 12, 14, 15, 16, 18, 19, 21, 26, 27, 32, 44], 'MM2': [6, 7, 11, 12, 14, 15, 16, 18, 19, 21, 26, 27, 32, 3
3, 36, 44, 45, 50, 53]}, 0.015625: {'I0': [0, 1, 2, 3, 4, 5, 9, 10, 13, 17, 20, 22, 23, 24, 25, 28, 29, 30, 31, 34, 35, 37, 38, 39, 40, 41, 42, 43, 46, 47, 48, 49, 51, 52], 'I': [], 'KL': [36,
45], 'Var': [], 'MM1': [8, 12, 14, 15, 18, 32, 44], 'MM2': [8, 12, 14, 15, 18, 27, 32, 33, 36, 44, 45, 50]}, 0.00390625: {'I0': [0, 1, 2, 3, 4, 5, 9, 10, 13, 17, 20, 22, 23, 24, 25, 28, 29, 30,
31, 34, 35, 37, 38, 39, 40, 41, 42, 43, 46, 47, 48, 49, 51, 52], 'I': [], 'KL': [45], 'Var': [], 'MM1': [12, 14, 15, 44], 'MM2': [8, 12, 14, 15, 32]}, 0.0009765625: {'I0': [0, 1, 2, 3, 4, 5, 9,
10, 13, 17, 20, 22, 23, 24, 25, 28, 29, 30, 31, 34, 35, 37, 38, 39, 40, 41, 42, 43, 46, 47, 48, 49, 51, 52], 'I': [], 'KL': [45], 'Var': [], 'MM1': [12], 'MM2': [12, 15, 32]}, 0.000244140625:
{'I0': [0, 1, 2, 3, 4, 5, 9, 10, 13, 17, 20, 22, 23, 24, 25, 28, 29, 30, 31, 34, 35, 37, 38, 39, 40, 41, 42, 43, 46, 47, 48, 49, 51, 52], 'I': [], 'KL': [], 'Var': [], 'MM1': [], 'MM2': [12, 1
5, 32]}}}
```


Look at S=2000 case

In [124…

```python
df = pd.DataFrame(records)
df['rho_rank'] = np.emath.logn(base, df['h'])
df.head()
I0_ = df.loc[(df['S']==n_sample[-1]) & (df['T']=="I0")]

kl_ = df.loc[(df['S']==n_sample[-1]) & (df['T']=="KL")]
var_ = df.loc[(df['S']==n_sample[-1]) & (df['T']=="Var")]
ll_ = df.loc[(df['S']==n_sample[-1]) & (df['T']=="LL")]
mm1_ = df.loc[(df['S']==n_sample[-1]) & (df['T']=="MM1")]
I_ = df.loc[(df['S']==n_sample[-1]) & (df['T']=="I")]

kl_ = kl_.explode(['khat', 'ndx'])
var_ = var_.explode(['khat', 'ndx'])
ll_ = ll_.explode(['khat', 'ndx'])
I_ = I_.explode(['khat', 'ndx'])
mm1_ = mm1_.explode(['khat', 'ndx'])

original = I_[['khat', 'ndx']].drop_duplicates()
```

In [125…

```python
fig = plt.figure(figsize=(4, 7))
ndx_high_khat = loo['KL']['ndx'].tolist()
ymax = (len(ndx_high_khat))
_ = plt.scatter(original.khat, np.array([ndx_high_khat.index(i) for i in original['ndx']]) ,color="black", s=12, alpha=0.6)
_ = plt.scatter(kl_.khat, np.array([ndx_high_khat.index(i) for i in kl_['ndx']]) + 0.2, s=1, color='green', alpha=0.2)
_ = plt.scatter(var_.khat, np.array([ndx_high_khat.index(i) for i in var_['ndx']]) - 0.2, s=1, color='purple', alpha=0.2)

kl_ = kl_[kl_.khat<3]
ll_ = ll_[ll_.khat<3]
var_ = var_[var_.khat<3]
_ = plt.axvline(x=0.7, linestyle='dashed', linewidth=2, color='red', alpha=0.5)

kl_vals = defaultdict(list)
var_vals = defaultdict(list)
ll_vals = defaultdict(list)

for x, y, s in zip( kl_.khat, kl_['ndx'], kl_['rho_rank'].values.astype('int')):
    kl_vals[y] += [x]

for x, y, s in zip( var_.khat, var_['ndx'], var_['rho_rank'].values.astype('int')):
    var_vals[y] += [x]

for x, v in kl_vals.items():
    plt.plot(min(v), ndx_high_khat.index(x)+0.2, color='green', alpha=0.3)
    plt.text(min(v), ndx_high_khat.index(x) + 0.2, "KL",  ha='center', va='center', color='green', fontsize=8)

for x, v in var_vals.items():
    plt.plot(min(v), ndx_high_khat.index(x)-0.2, color='purple', alpha=0.3)
    plt.text(min(v), ndx_high_khat.index(x)-0.2, "Var",  ha='center', va='center', color='blue', fontsize=8)


#for x, v in var_vals.items():
#    plt.text(min(v), x, "LL",  ha='center', va='center', color='purple', fontsize=8)

_ = plt.ylabel("observation")
_ = plt.ylim(-0.5, ymax - 0.5)
_ = plt.xlabel(r'estimated Pareto tail shape $\hat{k}$')
_ = plt.yticks(ticks=np.arange(ymax), labels=loo['KL']['ndx'])
_ = plt.xlim((-0.5, 3))
_ = plt.xticks([-0.5, 0, 0.7, 1.5, 2])


for y0, y1 in zip(np.arange(ymax+ 1)[::2]-0.5, np.arange(ymax + 1)[::2] + 0.5):
    plt.axhspan(y0, y1, color='black', alpha=0.1, zorder=0)
#_ = plt.xlim((-0.1, 2))
_ = plt.savefig("khat_relu.pdf", bbox_inches='tight')
```

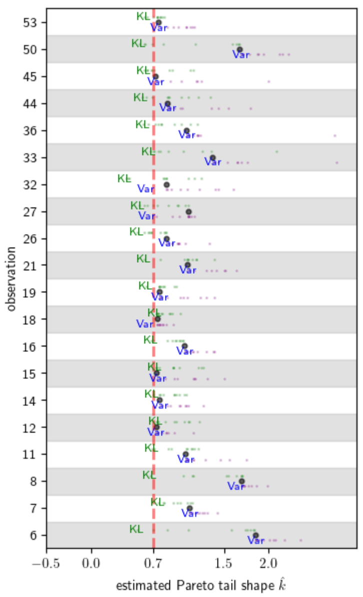

In [126…
```
len(ndx_high_khat)
```

Out[126… 20

In [127…
```
fig = plt.figure(figsize=(6, 9))

_ = plt.scatter(original.khat, np.array([ndx_high_khat.index(i) for i in original['ndx']]) ,color="black", s=12, alpha=0.6)
_ = plt.scatter(kl_.khat, np.array([ndx_high_khat.index(i) for i in kl_['ndx']]), s=0, color='green', alpha=0.2)
_ = plt.scatter(var_.khat, np.array([ndx_high_khat.index(i) for i in var_['ndx']]), s=0, color='purple', alpha=0.2)
_ = plt.scatter(ll_.khat, np.array([ndx_high_khat.index(i) for i in ll_['ndx']]), s=0, color='green', alpha=0.2)
_ = plt.scatter(mm1_.khat, np.array([ndx_high_khat.index(i) for i in mm1_['ndx']]), s=0, color='purple', alpha=0.2)

kl_ = kl_[kl_.khat<2]
ll_ = ll_[ll_.khat<2]
var_ = var_[var_.khat<2]
mm1_ = mm1_[mm1_.khat<2]
_ = plt.axvline(x=0.7, linestyle='dotted', linewidth=2, color='red')

for x, y, s in zip( kl_.khat, kl_['ndx'], kl_['rho_rank'].values.astype('int')):
    if s > 7:
        continue
    plt.text(x, ndx_high_khat.index(y) + 0.2, str(s),  ha='center', va='center', color='green', fontsize=8)

for x, y, s in zip( var_.khat, var_['ndx'], var_['rho_rank'].values.astype('int')):
    if s > 7:
        continue
    plt.text(x, ndx_high_khat.index(y) + 0.1, str(s),  ha='center', va='center', color='blue', fontsize=8)

for x, y, s in zip( ll_.khat, ll_['ndx'], ll_['rho_rank'].values.astype('int')):
    if s > 3:
        continue
    plt.text(x, ndx_high_khat.index(y) - 0.1, str(s),  ha='center', va='center', color='orange', fontsize=8)

for x, y, s in zip( mm1_.khat, mm1_['ndx'], mm1_['rho_rank'].values.astype('int')):
    if s > 3:
        continue
    plt.text(x, ndx_high_khat.index(y) - 0.2, str(s),  ha='center', va='center', color='red', fontsize=8)

_ = plt.ylabel("observation")
_ = plt.ylim(-0.5, ymax - 0.5)
_ = plt.xlabel(r'estimated Pareto tail shape $\hat{k}$')
_ = plt.yticks(ticks=np.arange(ymax), labels=loo['KL']['ndx'])
_ = plt.xlim((-0.5, 3))
_ = plt.xticks([-0.5, 0, 0.7, 1.5, 2])


for y0, y1 in zip(np.arange(ymax+ 1)[::2]-0.5, np.arange(ymax + 1)[::2] + 0.5):
    plt.axhspan(y0, y1, color='black', alpha=0.1, zorder=0)
#_ = plt.xlim((-0.1, 2))
_ = plt.savefig("khat_detailed_relu.pdf", bbox_inches='tight')
```

```
 43%|████      | 4274/10000 [33:15<44:33,  2.14it/s]
 10%|█         | 1030/10000 [25:10<3:39:17,  1.47s/it]
```

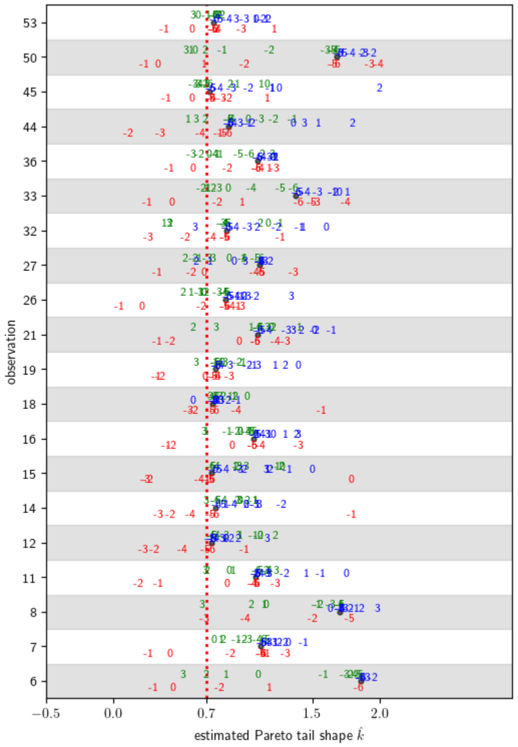

In [128…
```
df_ = df.explode(['khat', "p_psis", 'ndx'])[['ndx', 'p_psis', 'khat', 'T']]
#df_ = df_[df_['p_psis']!=1]
#df_ = df_[df_['p_psis']>1e-5]
df_ = df_[df_['T']!="MM2"]

df_ = df_.groupby(['ndx'])[['khat', 'p_psis']].min()

pd.set_option('display.max_rows', 500)

df_ = df_.reset_index()
df_['y'] = [y_.tolist()[i][0] for i in df_['ndx'].values]
```

In [129…
```
fpr, tpr, thresholds = metrics.roc_curve(df_['y'], df_.p_psis, pos_label=1)
precision, recall, thresholds_pr = metrics.precision_recall_curve(df_['y'], df_.p_psis)
```

In [130…
```
plt.plot(fpr, tpr)
plt.plot(recall, precision)
```

Out[130…
```
[<matplotlib.lines.Line2D at 0x7f21ca51d570>]
```

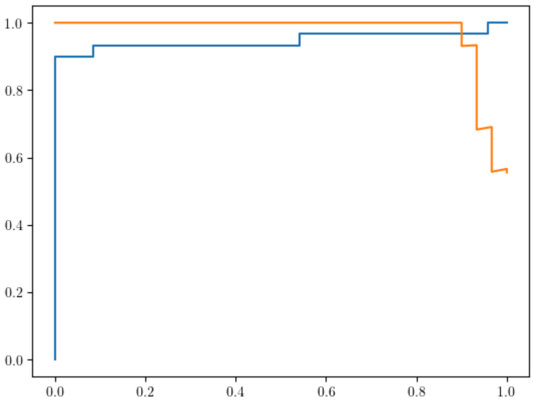

Cross-tabulations

In [131…
```
df__ = df.explode(['khat', 'ndx'])[['ndx', 'khat', 'T']]
transforms = ["KL", "Var", "LL", "MM1", "MM2"]
```

In [132…
```
for T in transforms:
    _df__ = df__.loc[(df__['T']==T) & (df__['khat']<0.7)]
    print(f"{T}: {_df__.groupby('ndx').min().shape[0]}")
print(len(df__[df__['T']!="I0"].ndx.unique()))
```

```
KL: 16
Var: 3
LL: 0
MM1: 20
MM2: 20
20
```

In [133…
```
print(f"{df__.loc[((df__['T']=='KL') | (df__['T']=='Var')) & (df__['khat']<0.7)].groupby('ndx').min().shape[0]}")
```

```
17
```

In [134…
```
print(f"{df__.loc[((df__['T']=='LL') | (df__['T']=='MM1')) &(df__['T']!='I0') & (df__['khat']<0.7)].groupby('ndx').min().shape[0]}")
```

```
20
```

In [135…
```
print(f"{df__.loc[(df__['T']!='MM1') &(df__['T']!='I0') & (df__['khat']<0.7)].groupby('ndx').min().shape[0]}")
```

```
20
```

In [136…
```
len(df__[df__['T']!="I0"].ndx.unique())
```

Out[136… 20

Compare with variational model

In [137…
```
lr_model2 = ShallowGaussianRelunet(
    dim_regressors=d,
    hidden_size=3
)
```

In [138…
```
losses = lr_model2.fit(
    data_factory_factory(shuffle=True, repeat=True),
    dataset_size=N,
    batches_per_step=9,
    check_every=int(N/batch_size)*50,
    batch_size=batch_size,
    num_steps=10000,
    max_decay_steps=10,
    max_plateau_epochs=100,
    sample_size=36,
    learning_rate=0.0005,
    lr_decay_factor=0.9
    )
```

```
Running optimization for 10000 steps of 9 accumulated batches, checking every 50 steps
Saved a checkpoint: /tmp/tfcheckpoints/d510a85a-e51a-4212-9da7-088b616c232f/d510a85a-e51a-4212-9da7-088b616c232f-1
  0%|          | 49/10000 [00:05<14:51, 11.16it/s]
Step 50: average-batch loss:18881.612214866844 rel loss: 5.296157916073652e+303
Saved a checkpoint: /tmp/tfcheckpoints/d510a85a-e51a-4212-9da7-088b616c232f/d510a85a-e51a-4212-9da7-088b616c232f-2
  1%|          | 99/10000 [00:09<14:51, 11.11it/s]
Step 100: average-batch loss:18786.37956286894 rel loss: 0.005069239215528761
Saved a checkpoint: /tmp/tfcheckpoints/d510a85a-e51a-4212-9da7-088b616c232f/d510a85a-e51a-4212-9da7-088b616c232f-3
  1%|▏         | 149/10000 [00:14<14:39, 11.20it/s]
Step 150: average-batch loss:18655.325915810925 rel loss: 0.007024999061900267
Saved a checkpoint: /tmp/tfcheckpoints/d510a85a-e51a-4212-9da7-088b616c232f/d510a85a-e51a-4212-9da7-088b616c232f-4
  2%|▏         | 199/10000 [00:19<14:58, 10.91it/s]
Step 200: average-batch loss:18508.82975635226 rel loss: 0.007914933649891407
Saved a checkpoint: /tmp/tfcheckpoints/d510a85a-e51a-4212-9da7-088b616c232f/d510a85a-e51a-4212-9da7-088b616c232f-5
  2%|▏         | 249/10000 [00:23<14:49, 10.96it/s]
Step 250: average-batch loss:18352.78312552288 rel loss: 0.008502614004759238
Saved a checkpoint: /tmp/tfcheckpoints/d510a85a-e51a-4212-9da7-088b616c232f/d510a85a-e51a-4212-9da7-088b616c232f-6
  3%|▎         | 299/10000 [00:28<14:51, 10.88it/s]
Step 300: average-batch loss:18207.71532734037 rel loss: 0.007967380617197975
Saved a checkpoint: /tmp/tfcheckpoints/d510a85a-e51a-4212-9da7-088b616c232f/d510a85a-e51a-4212-9da7-088b616c232f-7
  3%|▎         | 349/10000 [00:32<14:36, 11.01it/s]
Step 350: average-batch loss:18065.784413391633 rel loss: 0.007856338296804235
Saved a checkpoint: /tmp/tfcheckpoints/d510a85a-e51a-4212-9da7-088b616c232f/d510a85a-e51a-4212-9da7-088b616c232f-8
  4%|▍         | 399/10000 [00:37<14:33, 10.99it/s]
Step 400: average-batch loss:17928.038543375165 rel loss: 0.007683264941851005
Saved a checkpoint: /tmp/tfcheckpoints/d510a85a-e51a-4212-9da7-088b616c232f/d510a85a-e51a-4212-9da7-088b616c232f-9
  4%|▍         | 449/10000 [00:41<14:31, 10.96it/s]
Step 450: average-batch loss:17791.812630519722 rel loss: 0.007656662965400394
Saved a checkpoint: /tmp/tfcheckpoints/d510a85a-e51a-4212-9da7-088b616c232f/d510a85a-e51a-4212-9da7-088b616c232f-10
  5%|▍         | 499/10000 [00:46<15:20, 10.32it/s]
Step 500: average-batch loss:17663.02229876991 rel loss: 0.00729152290991459
Saved a checkpoint: /tmp/tfcheckpoints/d510a85a-e51a-4212-9da7-088b616c232f/d510a85a-e51a-4212-9da7-088b616c232f-11
  5%|▌         | 549/10000 [00:51<14:49, 10.63it/s]
Step 550: average-batch loss:17530.228631076534 rel loss: 0.007575124688218065
Saved a checkpoint: /tmp/tfcheckpoints/d510a85a-e51a-4212-9da7-088b616c232f/d510a85a-e51a-4212-9da7-088b616c232f-12
  6%|▌         | 599/10000 [00:56<14:38, 10.70it/s]
Step 600: average-batch loss:17401.745427381247 rel loss: 0.007383351528239304
Saved a checkpoint: /tmp/tfcheckpoints/d510a85a-e51a-4212-9da7-088b616c232f/d510a85a-e51a-4212-9da7-088b616c232f-13
  6%|▋         | 649/10000 [01:00<15:02, 10.36it/s]
Step 650: average-batch loss:17271.02727659943 rel loss: 0.007568637851607544
Saved a checkpoint: /tmp/tfcheckpoints/d510a85a-e51a-4212-9da7-088b616c232f/d510a85a-e51a-4212-9da7-088b616c232f-14
  7%|▋         | 699/10000 [01:05<14:08, 10.96it/s]
Step 700: average-batch loss:17141.458539587187 rel loss: 0.007558793011283835
Saved a checkpoint: /tmp/tfcheckpoints/d510a85a-e51a-4212-9da7-088b616c232f/d510a85a-e51a-4212-9da7-088b616c232f-15
  7%|▋         | 749/10000 [01:09<13:57, 11.05it/s]
Step 750: average-batch loss:17021.456480599616 rel loss: 0.007050046458970467
Saved a checkpoint: /tmp/tfcheckpoints/d510a85a-e51a-4212-9da7-088b616c232f/d510a85a-e51a-4212-9da7-088b616c232f-16
  8%|▊         | 799/10000 [01:14<13:55, 11.01it/s]
Step 800: average-batch loss:16899.171127811962 rel loss: 0.007236174594764704
Saved a checkpoint: /tmp/tfcheckpoints/d510a85a-e51a-4212-9da7-088b616c232f/d510a85a-e51a-4212-9da7-088b616c232f-17
  8%|▊         | 849/10000 [01:19<13:42, 11.12it/s]
Step 850: average-batch loss:16771.38460012096 rel loss: 0.007619318901677319
Saved a checkpoint: /tmp/tfcheckpoints/d510a85a-e51a-4212-9da7-088b616c232f/d510a85a-e51a-4212-9da7-088b616c232f-18
  9%|▉         | 899/10000 [01:23<13:51, 10.94it/s]
Step 900: average-batch loss:16649.90403097317 rel loss: 0.007296172333594418
Saved a checkpoint: /tmp/tfcheckpoints/d510a85a-e51a-4212-9da7-088b616c232f/d510a85a-e51a-4212-9da7-088b616c232f-19
  9%|▉         | 949/10000 [01:28<13:35, 11.09it/s]
Step 950: average-batch loss:16532.690459796242 rel loss: 0.007089806191071353
Saved a checkpoint: /tmp/tfcheckpoints/d510a85a-e51a-4212-9da7-088b616c232f/d510a85a-e51a-4212-9da7-088b616c232f-20
 10%|▉         | 999/10000 [01:32<13:37, 11.00it/s]
Step 1000: average-batch loss:16411.21370105442 rel loss: 0.0074020581874463115
Saved a checkpoint: /tmp/tfcheckpoints/d510a85a-e51a-4212-9da7-088b616c232f/d510a85a-e51a-4212-9da7-088b616c232f-21
 10%|█         | 1049/10000 [01:37<13:32, 11.02it/s]
Step 1050: average-batch loss:16287.213150740505 rel loss: 0.007613368178231194
Saved a checkpoint: /tmp/tfcheckpoints/d510a85a-e51a-4212-9da7-088b616c232f/d510a85a-e51a-4212-9da7-088b616c232f-22
 11%|█         | 1099/10000 [01:42<13:31, 10.96it/s]
Step 1100: average-batch loss:16169.213632805997 rel loss: 0.007297789528557936
Saved a checkpoint: /tmp/tfcheckpoints/d510a85a-e51a-4212-9da7-088b616c232f/d510a85a-e51a-4212-9da7-088b616c232f-23
 11%|█▏        | 1149/10000 [01:46<13:26, 10.97it/s]
Step 1150: average-batch loss:16045.168103734073 rel loss: 0.007731020845026581
Saved a checkpoint: /tmp/tfcheckpoints/d510a85a-e51a-4212-9da7-088b616c232f/d510a85a-e51a-4212-9da7-088b616c232f-24
 12%|█▏        | 1199/10000 [01:51<13:49, 10.61it/s]
Step 1200: average-batch loss:15927.77478245489 rel loss: 0.007370352913860709
Saved a checkpoint: /tmp/tfcheckpoints/d510a85a-e51a-4212-9da7-088b616c232f/d510a85a-e51a-4212-9da7-088b616c232f-25
 12%|█▏        | 1249/10000 [01:55<13:15, 11.00it/s]
Step 1250: average-batch loss:15812.748898958027 rel loss: 0.007274249672328778
Saved a checkpoint: /tmp/tfcheckpoints/d510a85a-e51a-4212-9da7-088b616c232f/d510a85a-e51a-4212-9da7-088b616c232f-26
 13%|█▎        | 1299/10000 [02:00<13:22, 10.84it/s]
Step 1300: average-batch loss:15687.185841116463 rel loss: 0.008004179915588197
Saved a checkpoint: /tmp/tfcheckpoints/d510a85a-e51a-4212-9da7-088b616c232f/d510a85a-e51a-4212-9da7-088b616c232f-27
 13%|█▎        | 1349/10000 [02:05<13:07, 10.99it/s]
Step 1350: average-batch loss:15574.35226911414 rel loss: 0.007244832404753376
Saved a checkpoint: /tmp/tfcheckpoints/d510a85a-e51a-4212-9da7-088b616c232f/d510a85a-e51a-4212-9da7-088b616c232f-28
```

```
 14%|█▍        | 1399/10000 [02:09<13:01, 11.00it/s]
Step 1400: average-batch loss:15451.434282055669 rel loss: 0.00795511826376024
Saved a checkpoint: /tmp/tfcheckpoints/d510a85a-e51a-4212-9da7-088b616c232f/d510a85a-e51a-4212-9da7-088b616c232f-29
 14%|█▍        | 1449/10000 [02:14<12:55, 11.03it/s]
Step 1450: average-batch loss:15337.04552811773 rel loss: 0.007458330467118195
Saved a checkpoint: /tmp/tfcheckpoints/d510a85a-e51a-4212-9da7-088b616c232f/d510a85a-e51a-4212-9da7-088b616c232f-30
 15%|█▍        | 1499/10000 [02:18<12:54, 10.97it/s]
Step 1500: average-batch loss:15223.598205290844 rel loss: 0.007452070219999471
Saved a checkpoint: /tmp/tfcheckpoints/d510a85a-e51a-4212-9da7-088b616c232f/d510a85a-e51a-4212-9da7-088b616c232f-31
 15%|█▌        | 1549/10000 [02:23<12:52, 10.94it/s]
Step 1550: average-batch loss:15107.30672014258 rel loss: 0.007697698028015294
Saved a checkpoint: /tmp/tfcheckpoints/d510a85a-e51a-4212-9da7-088b616c232f/d510a85a-e51a-4212-9da7-088b616c232f-32
 16%|█▌        | 1599/10000 [02:27<12:44, 10.99it/s]
Step 1600: average-batch loss:14991.117717965677 rel loss: 0.0077505229671874145
Saved a checkpoint: /tmp/tfcheckpoints/d510a85a-e51a-4212-9da7-088b616c232f/d510a85a-e51a-4212-9da7-088b616c232f-33
 16%|█▋        | 1649/10000 [02:32<12:45, 10.91it/s]
Step 1650: average-batch loss:14864.007746730029 rel loss: 0.008551527515424725
Saved a checkpoint: /tmp/tfcheckpoints/d510a85a-e51a-4212-9da7-088b616c232f/d510a85a-e51a-4212-9da7-088b616c232f-34
 17%|█▋        | 1699/10000 [02:37<12:38, 10.94it/s]
Step 1700: average-batch loss:14750.927717753693 rel loss: 0.007665960483301432
Saved a checkpoint: /tmp/tfcheckpoints/d510a85a-e51a-4212-9da7-088b616c232f/d510a85a-e51a-4212-9da7-088b616c232f-35
 17%|█▋        | 1749/10000 [02:41<12:31, 10.97it/s]
Step 1750: average-batch loss:14637.094564988889 rel loss: 0.00777703199630115
Saved a checkpoint: /tmp/tfcheckpoints/d510a85a-e51a-4212-9da7-088b616c232f/d510a85a-e51a-4212-9da7-088b616c232f-36
 18%|█▊        | 1799/10000 [02:46<12:31, 10.91it/s]
Step 1800: average-batch loss:14519.03814304339 rel loss: 0.008131146208336418
Saved a checkpoint: /tmp/tfcheckpoints/d510a85a-e51a-4212-9da7-088b616c232f/d510a85a-e51a-4212-9da7-088b616c232f-37
 18%|█▊        | 1849/10000 [02:50<12:25, 10.93it/s]
Step 1850: average-batch loss:14407.02695201781 rel loss: 0.007774760982861357
Saved a checkpoint: /tmp/tfcheckpoints/d510a85a-e51a-4212-9da7-088b616c232f/d510a85a-e51a-4212-9da7-088b616c232f-38
 19%|█▉        | 1899/10000 [02:55<12:19, 10.96it/s]
Step 1900: average-batch loss:14289.934111994302 rel loss: 0.008194078370538161
Saved a checkpoint: /tmp/tfcheckpoints/d510a85a-e51a-4212-9da7-088b616c232f/d510a85a-e51a-4212-9da7-088b616c232f-39
 19%|█▉        | 1949/10000 [03:00<12:14, 10.96it/s]
Step 1950: average-batch loss:14174.160219956078 rel loss: 0.008167954237967704
Saved a checkpoint: /tmp/tfcheckpoints/d510a85a-e51a-4212-9da7-088b616c232f/d510a85a-e51a-4212-9da7-088b616c232f-40
 20%|█▉        | 1999/10000 [03:04<12:08, 10.99it/s]
Step 2000: average-batch loss:14059.593862398142 rel loss: 0.008148624965927354
Saved a checkpoint: /tmp/tfcheckpoints/d510a85a-e51a-4212-9da7-088b616c232f/d510a85a-e51a-4212-9da7-088b616c232f-41
 20%|██        | 2049/10000 [03:09<12:01, 11.03it/s]
Step 2050: average-batch loss:13941.447743573 rel loss: 0.0084744512189996
Saved a checkpoint: /tmp/tfcheckpoints/d510a85a-e51a-4212-9da7-088b616c232f/d510a85a-e51a-4212-9da7-088b616c232f-42
 21%|██        | 2099/10000 [03:13<12:05, 10.89it/s]
Step 2100: average-batch loss:13831.268848801019 rel loss: 0.007965928215004837
Saved a checkpoint: /tmp/tfcheckpoints/d510a85a-e51a-4212-9da7-088b616c232f/d510a85a-e51a-4212-9da7-088b616c232f-43
 21%|██▏       | 2149/10000 [03:18<12:27, 10.51it/s]
Step 2150: average-batch loss:13712.787894599183 rel loss: 0.008640179889933226
Saved a checkpoint: /tmp/tfcheckpoints/d510a85a-e51a-4212-9da7-088b616c232f/d510a85a-e51a-4212-9da7-088b616c232f-44
 22%|██▏       | 2199/10000 [03:22<11:45, 11.06it/s]
Step 2200: average-batch loss:13595.9054743253 rel loss: 0.008596883855555317
Saved a checkpoint: /tmp/tfcheckpoints/d510a85a-e51a-4212-9da7-088b616c232f/d510a85a-e51a-4212-9da7-088b616c232f-45
 22%|██▏       | 2249/10000 [03:27<11:47, 10.96it/s]
Step 2250: average-batch loss:13481.37188313868 rel loss: 0.008495692588220125
Saved a checkpoint: /tmp/tfcheckpoints/d510a85a-e51a-4212-9da7-088b616c232f/d510a85a-e51a-4212-9da7-088b616c232f-46
 23%|██▎       | 2299/10000 [03:32<11:39, 11.01it/s]
Step 2300: average-batch loss:13364.674405022428 rel loss: 0.008731786093673699
Saved a checkpoint: /tmp/tfcheckpoints/d510a85a-e51a-4212-9da7-088b616c232f/d510a85a-e51a-4212-9da7-088b616c232f-47
 23%|██▎       | 2349/10000 [03:36<12:36, 10.11it/s]
Step 2350: average-batch loss:13252.08490592271 rel loss: 0.008495983831902502
Saved a checkpoint: /tmp/tfcheckpoints/d510a85a-e51a-4212-9da7-088b616c232f/d510a85a-e51a-4212-9da7-088b616c232f-48
 24%|██▍       | 2399/10000 [03:41<11:32, 10.98it/s]
Step 2400: average-batch loss:13134.987877307884 rel loss: 0.00891489430432773
Saved a checkpoint: /tmp/tfcheckpoints/d510a85a-e51a-4212-9da7-088b616c232f/d510a85a-e51a-4212-9da7-088b616c232f-49
 24%|██▍       | 2449/10000 [03:45<11:26, 11.00it/s]
Step 2450: average-batch loss:13016.53056368971 rel loss: 0.009100528980327236
Saved a checkpoint: /tmp/tfcheckpoints/d510a85a-e51a-4212-9da7-088b616c232f/d510a85a-e51a-4212-9da7-088b616c232f-50
 25%|██▍       | 2499/10000 [03:50<11:14, 11.12it/s]
Step 2500: average-batch loss:12907.302343097928 rel loss: 0.0084625135205104
Saved a checkpoint: /tmp/tfcheckpoints/d510a85a-e51a-4212-9da7-088b616c232f/d510a85a-e51a-4212-9da7-088b616c232f-51
 25%|██▌       | 2549/10000 [03:55<11:17, 10.99it/s]
Step 2550: average-batch loss:12791.804667149605 rel loss: 0.009029036868029326
Saved a checkpoint: /tmp/tfcheckpoints/d510a85a-e51a-4212-9da7-088b616c232f/d510a85a-e51a-4212-9da7-088b616c232f-52
 26%|██▌       | 2599/10000 [03:59<11:16, 10.94it/s]
Step 2600: average-batch loss:12684.453693904668 rel loss: 0.008463192490230975
Saved a checkpoint: /tmp/tfcheckpoints/d510a85a-e51a-4212-9da7-088b616c232f/d510a85a-e51a-4212-9da7-088b616c232f-53
 26%|██▋       | 2649/10000 [04:04<11:05, 11.05it/s]
Step 2650: average-batch loss:12562.883977457004 rel loss: 0.009676895581126945
Saved a checkpoint: /tmp/tfcheckpoints/d510a85a-e51a-4212-9da7-088b616c232f/d510a85a-e51a-4212-9da7-088b616c232f-54
 27%|██▋       | 2699/10000 [04:08<11:00, 11.06it/s]
Step 2700: average-batch loss:12453.191862081994 rel loss: 0.008808353439812098
Saved a checkpoint: /tmp/tfcheckpoints/d510a85a-e51a-4212-9da7-088b616c232f/d510a85a-e51a-4212-9da7-088b616c232f-55
 27%|██▋       | 2749/10000 [04:13<10:57, 11.02it/s]
Step 2750: average-batch loss:12339.157767614604 rel loss: 0.009241643280279968
Saved a checkpoint: /tmp/tfcheckpoints/d510a85a-e51a-4212-9da7-088b616c232f/d510a85a-e51a-4212-9da7-088b616c232f-56
 28%|██▊       | 2799/10000 [04:17<10:57, 10.95it/s]
Step 2800: average-batch loss:12224.556110110534 rel loss: 0.009374709107784044
Saved a checkpoint: /tmp/tfcheckpoints/d510a85a-e51a-4212-9da7-088b616c232f/d510a85a-e51a-4212-9da7-088b616c232f-57
 28%|██▊       | 2849/10000 [04:22<10:53, 10.94it/s]
Step 2850: average-batch loss:12111.00094945096 rel loss: 0.009376199468031785
Saved a checkpoint: /tmp/tfcheckpoints/d510a85a-e51a-4212-9da7-088b616c232f/d510a85a-e51a-4212-9da7-088b616c232f-58
 29%|██▉       | 2899/10000 [04:27<10:47, 10.96it/s]
Step 2900: average-batch loss:11996.381237980373 rel loss: 0.009554523918238118
Saved a checkpoint: /tmp/tfcheckpoints/d510a85a-e51a-4212-9da7-088b616c232f/d510a85a-e51a-4212-9da7-088b616c232f-59
 29%|██▉       | 2949/10000 [04:31<10:49, 10.85it/s]
Step 2950: average-batch loss:11888.577970516673 rel loss: 0.009067801694285784
Saved a checkpoint: /tmp/tfcheckpoints/d510a85a-e51a-4212-9da7-088b616c232f/d510a85a-e51a-4212-9da7-088b616c232f-60
 30%|██▉       | 2999/10000 [04:36<10:39, 10.95it/s]
Step 3000: average-batch loss:11774.80333392582 rel loss: 0.009662550903338094
Saved a checkpoint: /tmp/tfcheckpoints/d510a85a-e51a-4212-9da7-088b616c232f/d510a85a-e51a-4212-9da7-088b616c232f-61
 30%|███       | 3049/10000 [04:40<10:30, 11.03it/s]
Step 3050: average-batch loss:11655.126752703325 rel loss: 0.010268149266994176
Saved a checkpoint: /tmp/tfcheckpoints/d510a85a-e51a-4212-9da7-088b616c232f/d510a85a-e51a-4212-9da7-088b616c232f-62
 31%|███       | 3099/10000 [04:45<10:29, 10.96it/s]
Step 3100: average-batch loss:11547.103136311918 rel loss: 0.009355040404177862
Saved a checkpoint: /tmp/tfcheckpoints/d510a85a-e51a-4212-9da7-088b616c232f/d510a85a-e51a-4212-9da7-088b616c232f-63
 31%|███▏      | 3149/10000 [04:50<10:23, 10.98it/s]
Step 3150: average-batch loss:11431.659569890679 rel loss: 0.010098583299777503
Saved a checkpoint: /tmp/tfcheckpoints/d510a85a-e51a-4212-9da7-088b616c232f/d510a85a-e51a-4212-9da7-088b616c232f-64
 32%|███▏      | 3199/10000 [04:54<10:25, 10.88it/s]
```

```
Step 3200: average-batch loss:11321.38627830918 rel loss: 0.00974026403398778
Saved a checkpoint: /tmp/tfcheckpoints/d510a85a-e51a-4212-9da7-088b616c232f/d510a85a-e51a-4212-9da7-088b616c232f-65
 32%|███       | 3249/10000 [04:59<10:15, 10.96it/s]
Step 3250: average-batch loss:11206.293657816328 rel loss: 0.01027035557046788
Saved a checkpoint: /tmp/tfcheckpoints/d510a85a-e51a-4212-9da7-088b616c232f/d510a85a-e51a-4212-9da7-088b616c232f-66
 33%|███       | 3299/10000 [05:03<10:16, 10.86it/s]
Step 3300: average-batch loss:11095.855877611984 rel loss: 0.009953065488816689
Saved a checkpoint: /tmp/tfcheckpoints/d510a85a-e51a-4212-9da7-088b616c232f/d510a85a-e51a-4212-9da7-088b616c232f-67
 33%|███       | 3349/10000 [05:08<10:08, 10.93it/s]
Step 3350: average-batch loss:10982.222933778005 rel loss: 0.010346989359001193
Saved a checkpoint: /tmp/tfcheckpoints/d510a85a-e51a-4212-9da7-088b616c232f/d510a85a-e51a-4212-9da7-088b616c232f-68
 34%|███       | 3399/10000 [05:12<10:00, 10.98it/s]
Step 3400: average-batch loss:10875.274596545629 rel loss: 0.009834081547361229
Saved a checkpoint: /tmp/tfcheckpoints/d510a85a-e51a-4212-9da7-088b616c232f/d510a85a-e51a-4212-9da7-088b616c232f-69
 34%|███       | 3449/10000 [05:17<09:51, 11.07it/s]
Step 3450: average-batch loss:10762.635097299859 rel loss: 0.010465791902024906
Saved a checkpoint: /tmp/tfcheckpoints/d510a85a-e51a-4212-9da7-088b616c232f/d510a85a-e51a-4212-9da7-088b616c232f-70
 35%|███       | 3499/10000 [05:22<09:52, 10.97it/s]
Step 3500: average-batch loss:10649.03329303877 rel loss: 0.01066780440393116
Saved a checkpoint: /tmp/tfcheckpoints/d510a85a-e51a-4212-9da7-088b616c232f/d510a85a-e51a-4212-9da7-088b616c232f-71
 35%|███       | 3549/10000 [05:26<09:43, 11.06it/s]
Step 3550: average-batch loss:10530.63862708808 rel loss: 0.011242876158160347
Saved a checkpoint: /tmp/tfcheckpoints/d510a85a-e51a-4212-9da7-088b616c232f/d510a85a-e51a-4212-9da7-088b616c232f-72
 36%|███       | 3599/10000 [05:31<09:40, 11.03it/s]
Step 3600: average-batch loss:10422.257625204942 rel loss: 0.010398994707349 32
Saved a checkpoint: /tmp/tfcheckpoints/d510a85a-e51a-4212-9da7-088b616c232f/d510a85a-e51a-4212-9da7-088b616c232f-73
 36%|███       | 3649/10000 [05:35<09:38, 10.98it/s]
Step 3650: average-batch loss:10316.60516953823 rel loss: 0.010241009899135 34
Saved a checkpoint: /tmp/tfcheckpoints/d510a85a-e51a-4212-9da7-088b616c232f/d510a85a-e51a-4212-9da7-088b616c232f-74
 37%|███       | 3699/10000 [05:40<09:34, 10.97it/s]
Step 3700: average-batch loss:10204.908768241901 rel loss: 0.010945360103946526
Saved a checkpoint: /tmp/tfcheckpoints/d510a85a-e51a-4212-9da7-088b616c232f/d510a85a-e51a-4212-9da7-088b616c232f-75
 37%|███       | 3749/10000 [05:44<09:28, 10.99it/s]
Step 3750: average-batch loss:10088.336937934517 rel loss: 0.011555108738393447
Saved a checkpoint: /tmp/tfcheckpoints/d510a85a-e51a-4212-9da7-088b616c232f/d510a85a-e51a-4212-9da7-088b616c232f-76
 38%|███       | 3799/10000 [05:49<09:23, 11.00it/s]
Step 3800: average-batch loss:9979.331929620028 rel loss: 0.010923076723297174
Saved a checkpoint: /tmp/tfcheckpoints/d510a85a-e51a-4212-9da7-088b616c232f/d510a85a-e51a-4212-9da7-088b616c232f-77
 38%|███       | 3849/10000 [05:54<09:20, 10.98it/s]
Step 3850: average-batch loss:9871.456723484358 rel loss: 0.01092799261116478
Saved a checkpoint: /tmp/tfcheckpoints/d510a85a-e51a-4212-9da7-088b616c232f/d510a85a-e51a-4212-9da7-088b616c232f-78
 39%|███       | 3899/10000 [05:58<09:18, 10.92it/s]
Step 3900: average-batch loss:9756.207495454391 rel loss: 0.011812912761814823
Saved a checkpoint: /tmp/tfcheckpoints/d510a85a-e51a-4212-9da7-088b616c232f/d510a85a-e51a-4212-9da7-088b616c232f-79
 39%|███       | 3949/10000 [06:03<09:14, 10.91it/s]
Step 3950: average-batch loss:9644.851851220632 rel loss: 0.011545604427264117
Saved a checkpoint: /tmp/tfcheckpoints/d510a85a-e51a-4212-9da7-088b616c232f/d510a85a-e51a-4212-9da7-088b616c232f-80
 40%|████      | 3999/10000 [06:07<09:03, 11.05it/s]
Step 4000: average-batch loss:9538.533020511473 rel loss: 0.011146245495039198
Saved a checkpoint: /tmp/tfcheckpoints/d510a85a-e51a-4212-9da7-088b616c232f/d510a85a-e51a-4212-9da7-088b616c232f-81
 40%|████      | 4049/10000 [06:12<08:57, 11.07it/s]
Step 4050: average-batch loss:9422.937985358349 rel loss: 0.012267409096052607
Saved a checkpoint: /tmp/tfcheckpoints/d510a85a-e51a-4212-9da7-088b616c232f/d510a85a-e51a-4212-9da7-088b616c232f-82
 41%|████      | 4099/10000 [06:16<08:55, 11.03it/s]
Step 4100: average-batch loss:9315.18385705486 rel loss: 0.011567579336813658
Saved a checkpoint: /tmp/tfcheckpoints/d510a85a-e51a-4212-9da7-088b616c232f/d510a85a-e51a-4212-9da7-088b616c232f-83
 41%|████      | 4149/10000 [06:21<08:48, 11.06it/s]
Step 4150: average-batch loss:9195.012528984334 rel loss: 0.013069185897433479
Saved a checkpoint: /tmp/tfcheckpoints/d510a85a-e51a-4212-9da7-088b616c232f/d510a85a-e51a-4212-9da7-088b616c232f-84
 42%|████      | 4199/10000 [06:26<08:45, 11.05it/s]
Step 4200: average-batch loss:9099.475710805875 rel loss: 0.010499156348646236
Saved a checkpoint: /tmp/tfcheckpoints/d510a85a-e51a-4212-9da7-088b616c232f/d510a85a-e51a-4212-9da7-088b616c232f-85
 42%|████      | 4249/10000 [06:30<08:41, 11.03it/s]
Step 4250: average-batch loss:8982.539229455326 rel loss: 0.013018198792507771
Saved a checkpoint: /tmp/tfcheckpoints/d510a85a-e51a-4212-9da7-088b616c232f/d510a85a-e51a-4212-9da7-088b616c232f-86
 43%|████      | 4299/10000 [06:35<08:42, 10.91it/s]
Step 4300: average-batch loss:8872.206349939932 rel loss: 0.012435788254196868
Saved a checkpoint: /tmp/tfcheckpoints/d510a85a-e51a-4212-9da7-088b616c232f/d510a85a-e51a-4212-9da7-088b616c232f-87
 43%|████      | 4349/10000 [06:39<08:35, 10.96it/s]
Step 4350: average-batch loss:8768.120915971582 rel loss: 0.011870893999506001
Saved a checkpoint: /tmp/tfcheckpoints/d510a85a-e51a-4212-9da7-088b616c232f/d510a85a-e51a-4212-9da7-088b616c232f-88
 44%|████      | 4399/10000 [06:44<08:29, 10.99it/s]
Step 4400: average-batch loss:8653.14847045933 rel loss: 0.013286776010460537
Saved a checkpoint: /tmp/tfcheckpoints/d510a85a-e51a-4212-9da7-088b616c232f/d510a85a-e51a-4212-9da7-088b616c232f-89
 44%|████      | 4449/10000 [06:48<08:27, 10.94it/s]
Step 4450: average-batch loss:8548.627312948001 rel loss: 0.012226659753083076
Saved a checkpoint: /tmp/tfcheckpoints/d510a85a-e51a-4212-9da7-088b616c232f/d510a85a-e51a-4212-9da7-088b616c232f-90
 45%|████      | 4499/10000 [06:53<08:24, 10.91it/s]
Step 4500: average-batch loss:8439.040372509735 rel loss: 0.012985711123654198
Saved a checkpoint: /tmp/tfcheckpoints/d510a85a-e51a-4212-9da7-088b616c232f/d510a85a-e51a-4212-9da7-088b616c232f-91
 45%|████      | 4549/10000 [06:58<08:16, 10.98it/s]
Step 4550: average-batch loss:8326.751875433027 rel loss: 0.0134852699776008
Saved a checkpoint: /tmp/tfcheckpoints/d510a85a-e51a-4212-9da7-088b616c232f/d510a85a-e51a-4212-9da7-088b616c232f-92
 46%|████      | 4599/10000 [07:02<08:13, 10.95it/s]
Step 4600: average-batch loss:8221.924344336592 rel loss: 0.012749756225699354
Saved a checkpoint: /tmp/tfcheckpoints/d510a85a-e51a-4212-9da7-088b616c232f/d510a85a-e51a-4212-9da7-088b616c232f-93
 46%|████      | 4649/10000 [07:07<08:04, 11.04it/s]
Step 4650: average-batch loss:8114.581407841449 rel loss: 0.013228400961189896
Saved a checkpoint: /tmp/tfcheckpoints/d510a85a-e51a-4212-9da7-088b616c232f/d510a85a-e51a-4212-9da7-088b616c232f-94
 47%|████      | 4699/10000 [07:11<08:00, 11.03it/s]
Step 4700: average-batch loss:8005.182302369601 rel loss: 0.01366603549296623
Saved a checkpoint: /tmp/tfcheckpoints/d510a85a-e51a-4212-9da7-088b616c232f/d510a85a-e51a-4212-9da7-088b616c232f-95
 47%|████      | 4749/10000 [07:16<07:53, 11.10it/s]
Step 4750: average-batch loss:7892.545622387329 rel loss: 0.014271273853999152
Saved a checkpoint: /tmp/tfcheckpoints/d510a85a-e51a-4212-9da7-088b616c232f/d510a85a-e51a-4212-9da7-088b616c232f-96
 48%|████      | 4799/10000 [07:20<07:57, 10.89it/s]
Step 4800: average-batch loss:7784.658245380032 rel loss: 0.013858974100927925
Saved a checkpoint: /tmp/tfcheckpoints/d510a85a-e51a-4212-9da7-088b616c232f/d510a85a-e51a-4212-9da7-088b616c232f-97
 48%|████      | 4849/10000 [07:25<07:47, 11.01it/s]
Step 4850: average-batch loss:7677.30736228986 rel loss: 0.01398288202156245
Saved a checkpoint: /tmp/tfcheckpoints/d510a85a-e51a-4212-9da7-088b616c232f/d510a85a-e51a-4212-9da7-088b616c232f-98
 49%|████      | 4899/10000 [07:30<07:47, 10.90it/s]
Step 4900: average-batch loss:7569.304426186947 rel loss: 0.014268541734067833
Saved a checkpoint: /tmp/tfcheckpoints/d510a85a-e51a-4212-9da7-088b616c232f/d510a85a-e51a-4212-9da7-088b616c232f-99
 49%|████      | 4949/10000 [07:34<07:35, 11.09it/s]
Step 4950: average-batch loss:7468.737017220072 rel loss: 0.013465115820118617
Saved a checkpoint: /tmp/tfcheckpoints/d510a85a-e51a-4212-9da7-088b616c232f/d510a85a-e51a-4212-9da7-088b616c232f-100
 50%|█████     | 4999/10000 [07:39<07:35, 10.98it/s]
```

```
Step 5000: average-batch loss:7358.406347410793 rel loss: 0.01499382673370579
Saved a checkpoint: /tmp/tfcheckpoints/d510a85a-e51a-4212-9da7-088b616c232f/d510a85a-e51a-4212-9da7-088b616c232f-101
 50%|█████     | 5049/10000 [07:43<07:32, 10.95it/s]
Step 5050: average-batch loss:7257.268039393043 rel loss: 0.01393614063429423
Saved a checkpoint: /tmp/tfcheckpoints/d510a85a-e51a-4212-9da7-088b616c232f/d510a85a-e51a-4212-9da7-088b616c232f-102
 51%|█████     | 5099/10000 [07:48<07:27, 10.94it/s]
Step 5100: average-batch loss:7144.322802877877 rel loss: 0.015809089207121194
Saved a checkpoint: /tmp/tfcheckpoints/d510a85a-e51a-4212-9da7-088b616c232f/d510a85a-e51a-4212-9da7-088b616c232f-103
 51%|█████     | 5149/10000 [07:53<07:20, 11.00it/s]
Step 5150: average-batch loss:7035.981128275047 rel loss: 0.015398232688181034
Saved a checkpoint: /tmp/tfcheckpoints/d510a85a-e51a-4212-9da7-088b616c232f/d510a85a-e51a-4212-9da7-088b616c232f-104
 52%|█████     | 5199/10000 [07:57<07:16, 11.00it/s]
Step 5200: average-batch loss:6926.377655924911 rel loss: 0.015824068191889588
Saved a checkpoint: /tmp/tfcheckpoints/d510a85a-e51a-4212-9da7-088b616c232f/d510a85a-e51a-4212-9da7-088b616c232f-105
 52%|█████     | 5249/10000 [08:02<07:13, 10.95it/s]
Step 5250: average-batch loss:6825.65318233598 rel loss: 0.01475675234270536
Saved a checkpoint: /tmp/tfcheckpoints/d510a85a-e51a-4212-9da7-088b616c232f/d510a85a-e51a-4212-9da7-088b616c232f-106
 53%|█████     | 5299/10000 [08:06<07:10, 10.91it/s]
Step 5300: average-batch loss:6719.186083821268 rel loss: 0.01584523738240679
Saved a checkpoint: /tmp/tfcheckpoints/d510a85a-e51a-4212-9da7-088b616c232f/d510a85a-e51a-4212-9da7-088b616c232f-107
 53%|█████     | 5349/10000 [08:11<07:01, 11.03it/s]
Step 5350: average-batch loss:6618.107095770242 rel loss: 0.015273096459201618
Saved a checkpoint: /tmp/tfcheckpoints/d510a85a-e51a-4212-9da7-088b616c232f/d510a85a-e51a-4212-9da7-088b616c232f-108
 54%|█████     | 5399/10000 [08:15<07:00, 10.94it/s]
Step 5400: average-batch loss:6511.322283480361 rel loss: 0.016399865901400884
Saved a checkpoint: /tmp/tfcheckpoints/d510a85a-e51a-4212-9da7-088b616c232f/d510a85a-e51a-4212-9da7-088b616c232f-109
 54%|█████     | 5449/10000 [08:20<07:06, 10.66it/s]
Step 5450: average-batch loss:6407.733850571378 rel loss: 0.016166157228853402
Saved a checkpoint: /tmp/tfcheckpoints/d510a85a-e51a-4212-9da7-088b616c232f/d510a85a-e51a-4212-9da7-088b616c232f-110
 55%|█████     | 5499/10000 [08:25<06:52, 10.92it/s]
Step 5500: average-batch loss:6298.690498810761 rel loss: 0.017312066973477303
Saved a checkpoint: /tmp/tfcheckpoints/d510a85a-e51a-4212-9da7-088b616c232f/d510a85a-e51a-4212-9da7-088b616c232f-111
 55%|█████     | 5549/10000 [08:29<06:44, 10.99it/s]
Step 5550: average-batch loss:6198.842027130958 rel loss: 0.01610760062004941
Saved a checkpoint: /tmp/tfcheckpoints/d510a85a-e51a-4212-9da7-088b616c232f/d510a85a-e51a-4212-9da7-088b616c232f-112
 56%|█████     | 5599/10000 [08:34<06:47, 10.80it/s]
Step 5600: average-batch loss:6100.179898244123 rel loss: 0.016173642504417534
Saved a checkpoint: /tmp/tfcheckpoints/d510a85a-e51a-4212-9da7-088b616c232f/d510a85a-e51a-4212-9da7-088b616c232f-113
 56%|█████     | 5649/10000 [08:38<06:34, 11.03it/s]
Step 5650: average-batch loss:5993.824530973259 rel loss: 0.017744157627783332
Saved a checkpoint: /tmp/tfcheckpoints/d510a85a-e51a-4212-9da7-088b616c232f/d510a85a-e51a-4212-9da7-088b616c232f-114
 57%|█████     | 5699/10000 [08:43<06:29, 11.05it/s]
Step 5700: average-batch loss:5891.444829370904 rel loss: 0.017377689950002925
Saved a checkpoint: /tmp/tfcheckpoints/d510a85a-e51a-4212-9da7-088b616c232f/d510a85a-e51a-4212-9da7-088b616c232f-115
 57%|█████     | 5749/10000 [08:48<06:27, 10.98it/s]
Step 5750: average-batch loss:5791.44885149514 rel loss: 0.017266141934405337
Saved a checkpoint: /tmp/tfcheckpoints/d510a85a-e51a-4212-9da7-088b616c232f/d510a85a-e51a-4212-9da7-088b616c232f-116
 58%|█████     | 5799/10000 [08:52<06:21, 11.01it/s]
Step 5800: average-batch loss:5687.758546560295 rel loss: 0.01823043367365728
Saved a checkpoint: /tmp/tfcheckpoints/d510a85a-e51a-4212-9da7-088b616c232f/d510a85a-e51a-4212-9da7-088b616c232f-117
 58%|█████     | 5849/10000 [08:57<06:17, 10.99it/s]
Step 5850: average-batch loss:5584.612267538218 rel loss: 0.018469729693077874
Saved a checkpoint: /tmp/tfcheckpoints/d510a85a-e51a-4212-9da7-088b616c232f/d510a85a-e51a-4212-9da7-088b616c232f-118
 59%|█████     | 5899/10000 [09:01<06:13, 10.99it/s]
Step 5900: average-batch loss:5482.862730772095 rel loss: 0.018557739225361542
Saved a checkpoint: /tmp/tfcheckpoints/d510a85a-e51a-4212-9da7-088b616c232f/d510a85a-e51a-4212-9da7-088b616c232f-119
 59%|█████     | 5949/10000 [09:06<06:07, 11.02it/s]
Step 5950: average-batch loss:5382.066172154089 rel loss: 0.01872822730042056
Saved a checkpoint: /tmp/tfcheckpoints/d510a85a-e51a-4212-9da7-088b616c232f/d510a85a-e51a-4212-9da7-088b616c232f-120
 60%|██████    | 5999/10000 [09:11<06:08, 10.87it/s]
Step 6000: average-batch loss:5283.689967342484 rel loss: 0.018618845053296133
Saved a checkpoint: /tmp/tfcheckpoints/d510a85a-e51a-4212-9da7-088b616c232f/d510a85a-e51a-4212-9da7-088b616c232f-121
 60%|██████    | 6049/10000 [09:15<05:59, 10.99it/s]
Step 6050: average-batch loss:5177.128548892068 rel loss: 0.020583112326469324
Saved a checkpoint: /tmp/tfcheckpoints/d510a85a-e51a-4212-9da7-088b616c232f/d510a85a-e51a-4212-9da7-088b616c232f-122
 61%|██████    | 6099/10000 [09:20<05:55, 10.97it/s]
Step 6100: average-batch loss:5077.532417636216 rel loss: 0.019615065560175563
Saved a checkpoint: /tmp/tfcheckpoints/d510a85a-e51a-4212-9da7-088b616c232f/d510a85a-e51a-4212-9da7-088b616c232f-123
 61%|██████    | 6149/10000 [09:24<05:52, 10.91it/s]
Step 6150: average-batch loss:4976.881640172875 rel loss: 0.020223663076674017
Saved a checkpoint: /tmp/tfcheckpoints/d510a85a-e51a-4212-9da7-088b616c232f/d510a85a-e51a-4212-9da7-088b616c232f-124
 62%|██████    | 6199/10000 [09:29<05:52, 10.79it/s]
Step 6200: average-batch loss:4882.942712652288 rel loss: 0.019238179321084528
Saved a checkpoint: /tmp/tfcheckpoints/d510a85a-e51a-4212-9da7-088b616c232f/d510a85a-e51a-4212-9da7-088b616c232f-125
 62%|██████    | 6249/10000 [09:34<05:42, 10.95it/s]
Step 6250: average-batch loss:4781.856938699614 rel loss: 0.021139439186184245
Saved a checkpoint: /tmp/tfcheckpoints/d510a85a-e51a-4212-9da7-088b616c232f/d510a85a-e51a-4212-9da7-088b616c232f-126
 63%|██████    | 6299/10000 [09:38<05:44, 10.74it/s]
Step 6300: average-batch loss:4688.135451203755 rel loss: 0.019991207265949305
Saved a checkpoint: /tmp/tfcheckpoints/d510a85a-e51a-4212-9da7-088b616c232f/d510a85a-e51a-4212-9da7-088b616c232f-127
 63%|██████    | 6349/10000 [09:43<05:30, 11.03it/s]
Step 6350: average-batch loss:4590.827460808936 rel loss: 0.02119617677325491
Saved a checkpoint: /tmp/tfcheckpoints/d510a85a-e51a-4212-9da7-088b616c232f/d510a85a-e51a-4212-9da7-088b616c232f-128
 64%|██████    | 6399/10000 [09:47<05:32, 10.84it/s]
Step 6400: average-batch loss:4494.600359536947 rel loss: 0.021409489960060226
Saved a checkpoint: /tmp/tfcheckpoints/d510a85a-e51a-4212-9da7-088b616c232f/d510a85a-e51a-4212-9da7-088b616c232f-129
 64%|██████    | 6449/10000 [09:52<05:25, 10.91it/s]
Step 6450: average-batch loss:4386.887472012278 rel loss: 0.024553373710144606
Saved a checkpoint: /tmp/tfcheckpoints/d510a85a-e51a-4212-9da7-088b616c232f/d510a85a-e51a-4212-9da7-088b616c232f-130
 65%|██████    | 6499/10000 [09:57<05:18, 10.99it/s]
Step 6500: average-batch loss:4296.07661301707 rel loss: 0.021138091141124447
Saved a checkpoint: /tmp/tfcheckpoints/d510a85a-e51a-4212-9da7-088b616c232f/d510a85a-e51a-4212-9da7-088b616c232f-131
 65%|██████    | 6549/10000 [10:01<05:12, 11.04it/s]
Step 6550: average-batch loss:4201.2781703787305 rel loss: 0.022564190894742247
Saved a checkpoint: /tmp/tfcheckpoints/d510a85a-e51a-4212-9da7-088b616c232f/d510a85a-e51a-4212-9da7-088b616c232f-132
 66%|██████    | 6599/10000 [10:06<05:09, 10.99it/s]
Step 6600: average-batch loss:4104.938412909397 rel loss: 0.023469233342541734
Saved a checkpoint: /tmp/tfcheckpoints/d510a85a-e51a-4212-9da7-088b616c232f/d510a85a-e51a-4212-9da7-088b616c232f-133
 66%|██████    | 6649/10000 [10:11<05:06, 10.94it/s]
Step 6650: average-batch loss:4016.38614553703 rel loss: 0.022047747443498467
Saved a checkpoint: /tmp/tfcheckpoints/d510a85a-e51a-4212-9da7-088b616c232f/d510a85a-e51a-4212-9da7-088b616c232f-134
 67%|██████    | 6699/10000 [10:15<05:35,  9.85it/s]
Step 6700: average-batch loss:3917.635857960074 rel loss: 0.025206601929658567
Saved a checkpoint: /tmp/tfcheckpoints/d510a85a-e51a-4212-9da7-088b616c232f/d510a85a-e51a-4212-9da7-088b616c232f-135
 67%|██████    | 6748/10000 [10:20<04:56, 10.97it/s]
Step 6750: average-batch loss:3822.7213315570675 rel loss: 0.024829046684484893
Saved a checkpoint: /tmp/tfcheckpoints/d510a85a-e51a-4212-9da7-088b616c232f/d510a85a-e51a-4212-9da7-088b616c232f-136
 68%|██████    | 6798/10000 [10:24<04:55, 10.84it/s]
```

```
Step 6800: average-batch loss:3731.608734026811 rel loss: 0.024416439135068756
Saved a checkpoint: /tmp/tfcheckpoints/d510a85a-e51a-4212-9da7-088b616c232f/d510a85a-e51a-4212-9da7-088b616c232f-137
 68%|██████▊   | 6848/10000 [10:29<04:45, 11.03it/s]
Step 6850: average-batch loss:3641.4681004749527 rel loss: 0.024753926456228252
Saved a checkpoint: /tmp/tfcheckpoints/d510a85a-e51a-4212-9da7-088b616c232f/d510a85a-e51a-4212-9da7-088b616c232f-138
 69%|██████▉   | 6898/10000 [10:34<04:40, 11.05it/s]
Step 6900: average-batch loss:3551.1917684929026 rel loss: 0.025421418460981247
Saved a checkpoint: /tmp/tfcheckpoints/d510a85a-e51a-4212-9da7-088b616c232f/d510a85a-e51a-4212-9da7-088b616c232f-139
 69%|██████▉   | 6948/10000 [10:38<04:37, 10.98it/s]
Step 6950: average-batch loss:3461.0211243890462 rel loss: 0.026053190911908598
Saved a checkpoint: /tmp/tfcheckpoints/d510a85a-e51a-4212-9da7-088b616c232f/d510a85a-e51a-4212-9da7-088b616c232f-140
 70%|██████▉   | 6998/10000 [10:43<04:33, 10.99it/s]
Step 7000: average-batch loss:3371.6165878919146 rel loss: 0.026516815944671614
Saved a checkpoint: /tmp/tfcheckpoints/d510a85a-e51a-4212-9da7-088b616c232f/d510a85a-e51a-4212-9da7-088b616c232f-141
 70%|███████   | 7048/10000 [10:47<04:27, 11.05it/s]
Step 7050: average-batch loss:3279.1015169096177 rel loss: 0.028213542796774276
Saved a checkpoint: /tmp/tfcheckpoints/d510a85a-e51a-4212-9da7-088b616c232f/d510a85a-e51a-4212-9da7-088b616c232f-142
 71%|███████   | 7098/10000 [10:52<04:25, 10.94it/s]
Step 7100: average-batch loss:3194.18241776556 rel loss: 0.02658555086639717
Saved a checkpoint: /tmp/tfcheckpoints/d510a85a-e51a-4212-9da7-088b616c232f/d510a85a-e51a-4212-9da7-088b616c232f-143
 71%|███████▏  | 7148/10000 [10:57<04:22, 10.88it/s]
Step 7150: average-batch loss:3103.9507191164125 rel loss: 0.029069952075409623
Saved a checkpoint: /tmp/tfcheckpoints/d510a85a-e51a-4212-9da7-088b616c232f/d510a85a-e51a-4212-9da7-088b616c232f-144
 72%|███████▏  | 7198/10000 [11:01<04:13, 11.07it/s]
Step 7200: average-batch loss:3016.317992023237 rel loss: 0.02905288080531415
Saved a checkpoint: /tmp/tfcheckpoints/d510a85a-e51a-4212-9da7-088b616c232f/d510a85a-e51a-4212-9da7-088b616c232f-145
 72%|███████▏  | 7248/10000 [11:06<04:09, 11.02it/s]
Step 7250: average-batch loss:2931.6568339157075 rel loss: 0.028878263352007202
Saved a checkpoint: /tmp/tfcheckpoints/d510a85a-e51a-4212-9da7-088b616c232f/d510a85a-e51a-4212-9da7-088b616c232f-146
 73%|███████▎  | 7298/10000 [11:10<04:10, 10.79it/s]
Step 7300: average-batch loss:2848.852997739658 rel loss: 0.02906567528817666
Saved a checkpoint: /tmp/tfcheckpoints/d510a85a-e51a-4212-9da7-088b616c232f/d510a85a-e51a-4212-9da7-088b616c232f-147
 73%|███████▎  | 7348/10000 [11:15<04:04, 10.83it/s]
Step 7350: average-batch loss:2759.3462270583505 rel loss: 0.03243767302689221
Saved a checkpoint: /tmp/tfcheckpoints/d510a85a-e51a-4212-9da7-088b616c232f/d510a85a-e51a-4212-9da7-088b616c232f-148
 74%|███████▍  | 7398/10000 [11:20<04:00, 10.83it/s]
Step 7400: average-batch loss:2676.724804751005 rel loss: 0.03086661062829389
Saved a checkpoint: /tmp/tfcheckpoints/d510a85a-e51a-4212-9da7-088b616c232f/d510a85a-e51a-4212-9da7-088b616c232f-149
 74%|███████▍  | 7448/10000 [11:24<03:59, 10.66it/s]
Step 7450: average-batch loss:2593.387625680588 rel loss: 0.0321344862777877
Saved a checkpoint: /tmp/tfcheckpoints/d510a85a-e51a-4212-9da7-088b616c232f/d510a85a-e51a-4212-9da7-088b616c232f-150
 75%|███████▍  | 7498/10000 [11:29<03:45, 11.09it/s]
Step 7500: average-batch loss:2510.4441216476557 rel loss: 0.03303937471370392
Saved a checkpoint: /tmp/tfcheckpoints/d510a85a-e51a-4212-9da7-088b616c232f/d510a85a-e51a-4212-9da7-088b616c232f-151
 75%|███████▌  | 7548/10000 [11:33<03:46, 10.82it/s]
Step 7550: average-batch loss:2434.590229334429 rel loss: 0.031156738985995153
Saved a checkpoint: /tmp/tfcheckpoints/d510a85a-e51a-4212-9da7-088b616c232f/d510a85a-e51a-4212-9da7-088b616c232f-152
 76%|███████▌  | 7598/10000 [11:38<03:53, 10.29it/s]
Step 7600: average-batch loss:2355.0173879977597 rel loss: 0.03378864281096547
Saved a checkpoint: /tmp/tfcheckpoints/d510a85a-e51a-4212-9da7-088b616c232f/d510a85a-e51a-4212-9da7-088b616c232f-153
 76%|███████▋  | 7648/10000 [11:43<03:54, 10.03it/s]
Step 7650: average-batch loss:2268.8942305058135 rel loss: 0.03795820727736011
Saved a checkpoint: /tmp/tfcheckpoints/d510a85a-e51a-4212-9da7-088b616c232f/d510a85a-e51a-4212-9da7-088b616c232f-154
 77%|███████▋  | 7698/10000 [11:48<03:29, 10.99it/s]
Step 7700: average-batch loss:2193.7020340964173 rel loss: 0.03427639453339333
Saved a checkpoint: /tmp/tfcheckpoints/d510a85a-e51a-4212-9da7-088b616c232f/d510a85a-e51a-4212-9da7-088b616c232f-155
 77%|███████▋  | 7748/10000 [11:52<03:43, 10.08it/s]
Step 7750: average-batch loss:2111.5015609358034 rel loss: 0.03892986615846176
Saved a checkpoint: /tmp/tfcheckpoints/d510a85a-e51a-4212-9da7-088b616c232f/d510a85a-e51a-4212-9da7-088b616c232f-156
 78%|███████▊  | 7798/10000 [11:57<03:21, 10.94it/s]
Step 7800: average-batch loss:2036.5235675745907 rel loss: 0.03681665881751031
Saved a checkpoint: /tmp/tfcheckpoints/d510a85a-e51a-4212-9da7-088b616c232f/d510a85a-e51a-4212-9da7-088b616c232f-157
 78%|███████▊  | 7848/10000 [12:02<03:17, 10.92it/s]
Step 7850: average-batch loss:1961.8793615367022 rel loss: 0.038047296638780696
Saved a checkpoint: /tmp/tfcheckpoints/d510a85a-e51a-4212-9da7-088b616c232f/d510a85a-e51a-4212-9da7-088b616c232f-158
 79%|███████▉  | 7898/10000 [12:06<03:12, 10.95it/s]
Step 7900: average-batch loss:1885.0884103317012 rel loss: 0.040735994547591955
Saved a checkpoint: /tmp/tfcheckpoints/d510a85a-e51a-4212-9da7-088b616c232f/d510a85a-e51a-4212-9da7-088b616c232f-159
 79%|███████▉  | 7948/10000 [12:11<03:07, 10.92it/s]
Step 7950: average-batch loss:1818.4524864099021 rel loss: 0.03664430301027865
Saved a checkpoint: /tmp/tfcheckpoints/d510a85a-e51a-4212-9da7-088b616c232f/d510a85a-e51a-4212-9da7-088b616c232f-160
 80%|███████▉  | 7998/10000 [12:15<03:06, 10.73it/s]
Step 8000: average-batch loss:1745.679862551154 rel loss: 0.04168726776305794
Saved a checkpoint: /tmp/tfcheckpoints/d510a85a-e51a-4212-9da7-088b616c232f/d510a85a-e51a-4212-9da7-088b616c232f-161
 80%|████████  | 8048/10000 [12:20<02:57, 11.01it/s]
Step 8050: average-batch loss:1672.7533684466734 rel loss: 0.04359668046712732
Saved a checkpoint: /tmp/tfcheckpoints/d510a85a-e51a-4212-9da7-088b616c232f/d510a85a-e51a-4212-9da7-088b616c232f-162
 81%|████████  | 8098/10000 [12:25<02:53, 10.97it/s]
Step 8100: average-batch loss:1603.7146240497655 rel loss: 0.04304927033873928
Saved a checkpoint: /tmp/tfcheckpoints/d510a85a-e51a-4212-9da7-088b616c232f/d510a85a-e51a-4212-9da7-088b616c232f-163
 81%|████████▏ | 8148/10000 [12:29<02:49, 10.92it/s]
Step 8150: average-batch loss:1531.6740542234961 rel loss: 0.04703387749346667
Saved a checkpoint: /tmp/tfcheckpoints/d510a85a-e51a-4212-9da7-088b616c232f/d510a85a-e51a-4212-9da7-088b616c232f-164
 82%|████████▏ | 8198/10000 [12:34<02:43, 11.02it/s]
Step 8200: average-batch loss:1466.726063242174 rel loss: 0.04428092784944156
Saved a checkpoint: /tmp/tfcheckpoints/d510a85a-e51a-4212-9da7-088b616c232f/d510a85a-e51a-4212-9da7-088b616c232f-165
 82%|████████▏ | 8248/10000 [12:38<02:38, 11.03it/s]
Step 8250: average-batch loss:1399.1294548694782 rel loss: 0.0483133337929775
Saved a checkpoint: /tmp/tfcheckpoints/d510a85a-e51a-4212-9da7-088b616c232f/d510a85a-e51a-4212-9da7-088b616c232f-166
 83%|████████▎ | 8298/10000 [12:43<02:34, 11.04it/s]
Step 8300: average-batch loss:1337.8948446131067 rel loss: 0.04576937455355797
Saved a checkpoint: /tmp/tfcheckpoints/d510a85a-e51a-4212-9da7-088b616c232f/d510a85a-e51a-4212-9da7-088b616c232f-167
 83%|████████▎ | 8348/10000 [12:48<02:30, 10.97it/s]
Step 8350: average-batch loss:1271.7829772076839 rel loss: 0.05198360773044586
Saved a checkpoint: /tmp/tfcheckpoints/d510a85a-e51a-4212-9da7-088b616c232f/d510a85a-e51a-4212-9da7-088b616c232f-168
 84%|████████▍ | 8398/10000 [12:52<02:26, 10.91it/s]
Step 8400: average-batch loss:1213.7475364760046 rel loss: 0.04781508426388194
Saved a checkpoint: /tmp/tfcheckpoints/d510a85a-e51a-4212-9da7-088b616c232f/d510a85a-e51a-4212-9da7-088b616c232f-169
 84%|████████▍ | 8448/10000 [12:57<02:25, 10.67it/s]
Step 8450: average-batch loss:1146.5852763886965 rel loss: 0.05857589615910949
Saved a checkpoint: /tmp/tfcheckpoints/d510a85a-e51a-4212-9da7-088b616c232f/d510a85a-e51a-4212-9da7-088b616c232f-170
 85%|████████▍ | 8498/10000 [13:02<02:17, 10.94it/s]
Step 8500: average-batch loss:1088.200691501568 rel loss: 0.053652405611473934
Saved a checkpoint: /tmp/tfcheckpoints/d510a85a-e51a-4212-9da7-088b616c232f/d510a85a-e51a-4212-9da7-088b616c232f-171
 85%|████████▌ | 8548/10000 [13:06<02:14, 10.83it/s]
Step 8550: average-batch loss:1031.0385789311365 rel loss: 0.05544129360289369
Saved a checkpoint: /tmp/tfcheckpoints/d510a85a-e51a-4212-9da7-088b616c232f/d510a85a-e51a-4212-9da7-088b616c232f-172
 86%|████████▌ | 8598/10000 [13:11<02:07, 11.00it/s]
```

```
Step 8600: average-batch loss:971.0115588723947 rel loss: 0.06181905818757631
Saved a checkpoint: /tmp/tfcheckpoints/d510a85a-e51a-4212-9da7-088b616c232f/d510a85a-e51a-4212-9da7-088b616c232f-173
 86%|████████▌ | 8648/10000 [13:15<02:03, 10.99it/s]
Step 8650: average-batch loss:917.5285028145053 rel loss: 0.05829034835847714
Saved a checkpoint: /tmp/tfcheckpoints/d510a85a-e51a-4212-9da7-088b616c232f/d510a85a-e51a-4212-9da7-088b616c232f-174
 87%|████████▋ | 8698/10000 [13:20<01:58, 10.99it/s]
Step 8700: average-batch loss:863.5519068798003 rel loss: 0.06250532886868847
Saved a checkpoint: /tmp/tfcheckpoints/d510a85a-e51a-4212-9da7-088b616c232f/d510a85a-e51a-4212-9da7-088b616c232f-175
 87%|████████▋ | 8748/10000 [13:25<01:54, 10.97it/s]
Step 8750: average-batch loss:812.6422512188902 rel loss: 0.06264706958634031
Saved a checkpoint: /tmp/tfcheckpoints/d510a85a-e51a-4212-9da7-088b616c232f/d510a85a-e51a-4212-9da7-088b616c232f-176
 88%|████████▊ | 8798/10000 [13:29<01:49, 10.99it/s]
Step 8800: average-batch loss:760.6119748355202 rel loss: 0.06840580756649463
Saved a checkpoint: /tmp/tfcheckpoints/d510a85a-e51a-4212-9da7-088b616c232f/d510a85a-e51a-4212-9da7-088b616c232f-177
 88%|████████▊ | 8848/10000 [13:34<01:45, 10.96it/s]
Step 8850: average-batch loss:710.036957760123 rel loss: 0.07122871073491834
Saved a checkpoint: /tmp/tfcheckpoints/d510a85a-e51a-4212-9da7-088b616c232f/d510a85a-e51a-4212-9da7-088b616c232f-178
 89%|████████▉ | 8898/10000 [13:38<01:40, 10.95it/s]
Step 8900: average-batch loss:662.626100756744 rel loss: 0.0715499388708564
Saved a checkpoint: /tmp/tfcheckpoints/d510a85a-e51a-4212-9da7-088b616c232f/d510a85a-e51a-4212-9da7-088b616c232f-179
 89%|████████▉ | 8948/10000 [13:43<01:35, 11.03it/s]
Step 8950: average-batch loss:614.8888904811109 rel loss: 0.07763550621036878
Saved a checkpoint: /tmp/tfcheckpoints/d510a85a-e51a-4212-9da7-088b616c232f/d510a85a-e51a-4212-9da7-088b616c232f-180
 90%|████████▉ | 8998/10000 [13:48<01:32, 10.87it/s]
Step 9000: average-batch loss:572.73187523162 rel loss: 0.07360689542980657
Saved a checkpoint: /tmp/tfcheckpoints/d510a85a-e51a-4212-9da7-088b616c232f/d510a85a-e51a-4212-9da7-088b616c232f-181
 90%|█████████ | 9048/10000 [13:52<01:27, 10.89it/s]
Step 9050: average-batch loss:528.5639011638294 rel loss: 0.08356222203320819
Saved a checkpoint: /tmp/tfcheckpoints/d510a85a-e51a-4212-9da7-088b616c232f/d510a85a-e51a-4212-9da7-088b616c232f-182
 91%|█████████ | 9098/10000 [13:57<01:22, 10.99it/s]
Step 9100: average-batch loss:491.8706651216367 rel loss: 0.07459935841695033
Saved a checkpoint: /tmp/tfcheckpoints/d510a85a-e51a-4212-9da7-088b616c232f/d510a85a-e51a-4212-9da7-088b616c232f-183
 91%|█████████▏| 9148/10000 [14:01<01:20, 10.64it/s]
Step 9150: average-batch loss:449.40900024847946 rel loss: 0.09448334334577206
Saved a checkpoint: /tmp/tfcheckpoints/d510a85a-e51a-4212-9da7-088b616c232f/d510a85a-e51a-4212-9da7-088b616c232f-184
 92%|█████████▏| 9198/10000 [14:06<01:13, 10.95it/s]
Step 9200: average-batch loss:408.87018550665186 rel loss: 0.09914837564297795
Saved a checkpoint: /tmp/tfcheckpoints/d510a85a-e51a-4212-9da7-088b616c232f/d510a85a-e51a-4212-9da7-088b616c232f-185
 92%|█████████▏| 9248/10000 [14:11<01:08, 10.95it/s]
Step 9250: average-batch loss:374.81300921701757 rel loss: 0.09086444560923743
Saved a checkpoint: /tmp/tfcheckpoints/d510a85a-e51a-4212-9da7-088b616c232f/d510a85a-e51a-4212-9da7-088b616c232f-186
 93%|█████████▎| 9298/10000 [14:15<01:03, 11.07it/s]
Step 9300: average-batch loss:340.10108606109696 rel loss: 0.10206354692347212
Saved a checkpoint: /tmp/tfcheckpoints/d510a85a-e51a-4212-9da7-088b616c232f/d510a85a-e51a-4212-9da7-088b616c232f-187
 93%|█████████▎| 9348/10000 [14:20<00:59, 10.87it/s]
Step 9350: average-batch loss:308.45132166329984 rel loss: 0.1026086198208788
Saved a checkpoint: /tmp/tfcheckpoints/d510a85a-e51a-4212-9da7-088b616c232f/d510a85a-e51a-4212-9da7-088b616c232f-188
 94%|█████████▍| 9398/10000 [14:24<00:55, 10.82it/s]
Step 9400: average-batch loss:277.41446795579407 rel loss: 0.11187900161159398
Saved a checkpoint: /tmp/tfcheckpoints/d510a85a-e51a-4212-9da7-088b616c232f/d510a85a-e51a-4212-9da7-088b616c232f-189
 94%|█████████▍| 9448/10000 [14:29<00:50, 10.87it/s]
Step 9450: average-batch loss:248.09388360288767 rel loss: 0.118183422852288
Saved a checkpoint: /tmp/tfcheckpoints/d510a85a-e51a-4212-9da7-088b616c232f/d510a85a-e51a-4212-9da7-088b616c232f-190
 95%|█████████▍| 9498/10000 [14:33<00:45, 11.04it/s]
Step 9500: average-batch loss:224.5717097914474 rel loss: 0.10474237308557058
Saved a checkpoint: /tmp/tfcheckpoints/d510a85a-e51a-4212-9da7-088b616c232f/d510a85a-e51a-4212-9da7-088b616c232f-191
 95%|█████████▌| 9548/10000 [14:38<00:41, 10.98it/s]
Step 9550: average-batch loss:200.10866942016557 rel loss: 0.12224877833711996
Saved a checkpoint: /tmp/tfcheckpoints/d510a85a-e51a-4212-9da7-088b616c232f/d510a85a-e51a-4212-9da7-088b616c232f-192
 96%|█████████▌| 9598/10000 [14:43<00:36, 11.03it/s]
Step 9600: average-batch loss:176.06349262292542 rel loss: 0.1365710542204091
Saved a checkpoint: /tmp/tfcheckpoints/d510a85a-e51a-4212-9da7-088b616c232f/d510a85a-e51a-4212-9da7-088b616c232f-193
 96%|█████████▋| 9648/10000 [14:47<00:32, 10.99it/s]
Step 9650: average-batch loss:156.96383824949356 rel loss: 0.1216818764527981
Saved a checkpoint: /tmp/tfcheckpoints/d510a85a-e51a-4212-9da7-088b616c232f/d510a85a-e51a-4212-9da7-088b616c232f-194
 97%|█████████▋| 9698/10000 [14:52<00:27, 10.91it/s]
Step 9700: average-batch loss:140.08748261582443 rel loss: 0.12047011851837477
Saved a checkpoint: /tmp/tfcheckpoints/d510a85a-e51a-4212-9da7-088b616c232f/d510a85a-e51a-4212-9da7-088b616c232f-195
 97%|█████████▋| 9748/10000 [14:56<00:22, 10.99it/s]
Step 9750: average-batch loss:121.31709814230378 rel loss: 0.15472167370425538
Saved a checkpoint: /tmp/tfcheckpoints/d510a85a-e51a-4212-9da7-088b616c232f/d510a85a-e51a-4212-9da7-088b616c232f-196
 98%|█████████▊| 9798/10000 [15:01<00:18, 10.80it/s]
Step 9800: average-batch loss:108.58910374628861 rel loss: 0.11721244541950815
Saved a checkpoint: /tmp/tfcheckpoints/d510a85a-e51a-4212-9da7-088b616c232f/d510a85a-e51a-4212-9da7-088b616c232f-197
 98%|█████████▊| 9848/10000 [15:06<00:13, 10.94it/s]
Step 9850: average-batch loss:95.84344485992958 rel loss: 0.13298414831589353
Saved a checkpoint: /tmp/tfcheckpoints/d510a85a-e51a-4212-9da7-088b616c232f/d510a85a-e51a-4212-9da7-088b616c232f-198
 99%|█████████▉| 9898/10000 [15:10<00:09, 10.90it/s]
Step 9900: average-batch loss:85.17776177507837 rel loss: 0.12521675684570308
Saved a checkpoint: /tmp/tfcheckpoints/d510a85a-e51a-4212-9da7-088b616c232f/d510a85a-e51a-4212-9da7-088b616c232f-199
 99%|█████████▉| 9948/10000 [15:15<00:04, 10.97it/s]
Step 9950: average-batch loss:76.07320194096006 rel loss: 0.11968156462224763
Saved a checkpoint: /tmp/tfcheckpoints/d510a85a-e51a-4212-9da7-088b616c232f/d510a85a-e51a-4212-9da7-088b616c232f-200
100%|█████████▉| 9998/10000 [15:19<00:00, 11.04it/s]
Step 10000: average-batch loss:69.26030562134594 rel loss: 0.09836653561508975
Saved a checkpoint: /tmp/tfcheckpoints/d510a85a-e51a-4212-9da7-088b616c232f/d510a85a-e51a-4212-9da7-088b616c232f-201
100%|██████████| 10000/10000 [15:20<00:00, 10.42it/s]
Terminating because we are out of iterations
100%|██████████| 10000/10000 [15:20<00:00, 10.87it/s]
```

In [139…

```
p_ = lr_model2.sample(5)
{k: p.shape for k, p in p_.items()}
```

Out[139…

```
{'w_1': TensorShape([5, 3, 1]),
 'w_0': TensorShape([5, 1536, 3]),
 'b_1': TensorShape([5, 1]),
 'b_0': TensorShape([5, 3])}
```

In [140…

```
base = 4
h_vals = [4**-r for r in range(9)]

loo_output = []

for _ in tqdm(range(1)):
    loo_khat_advi = {}
    reduced_ndx_advi = {}
    n_sample = [1000]
    records_advi = []
    for n_samples in n_sample:
        reduced_ndx_advi[n_samples] = {}
```

```
        # print(f"Samples: {n_samples}")
        loo_khat_advi[n_samples] = {}
        params_ = lr_model2.sample(n_samples)

        for h in h_vals:
            loo_khat_advi[n_samples][h] = {}
            loo = adaptive_is_loo(lr_model2, batch, params_, h, variational=False)
            reduced_ndx_advi[n_samples][h] = {}
            # print(f"rho={h}\n")
            for T in ["I0", "I", "KL", "Var", "MM1", "MM2"]:
                loo_khat_advi[n_samples][h][T] = np.array(loo[T]["khat"])

                records_advi += [
                    {
                        "h": h,
                        "T": T,
                        "S": n_samples,
                        "khat": loo[T]["khat"],
                        "p_psis": loo[T]["p_psis"].numpy(),
                        "n>0.7": (np.where((loo[T]["khat"] > 0.7))[0]).shape,
                        "ndx": loo[T]["ndx"]
                    }
                ]
                reduced_ndx_advi[n_samples][h][T] = [loo[T]["ndx"][k] for k in np.where((loo[T]["khat"] <= 0.7))[0]]
                # print(reduced_ndx[n_samples][h])


print(reduced_ndx_advi)
```

```
100%|██████████| 1/1 [04:28<00:00, 268.96s/it]
{1000: {1: {'I0': [7], 'I': [], 'KL': [], 'Var': [], 'MM1': [], 'MM2': []}, 0.25: {'I0': [7], 'I': [], 'KL': [], 'Var': [], 'MM1': [], 'MM2': []}, 0.0625: {'I0': [7], 'I': [], 'KL': [], 'Var': [], 'MM1': [6, 8, 26, 30, 39, 41, 43, 48], 'MM2': [8, 11, 30, 32, 39, 48]}, 0.015625: {'I0': [7], 'I': [], 'KL': [], 'Var': [], 'MM1': [2, 6, 8, 11, 26, 27, 33, 36, 41, 45, 50], 'MM2': [6, 8, 11, 22, 24, 26, 27, 33, 36, 38, 40, 41, 45, 48, 50, 52, 53]}, 0.00390625: {'I0': [7], 'I': [], 'KL': [], 'Var': [], 'MM1': [8, 36], 'MM2': [8, 36, 50]}, 0.0009765625: {'I0': [7], 'I': [], 'KL': [], 'Var': [], 'MM1': [], 'MM2': []}, 0.000244140625: {'I0': [7], 'I': [], 'KL': [], 'Var': [], 'MM1': [], 'MM2': []}, 6.103515625e-05: {'I0': [7], 'I': [], 'KL': [], 'Var': [], 'MM1': [], 'MM2': []}, 1.52587890625e-05: {'I0': [7], 'I': [], 'KL': [], 'Var': [], 'MM1': [], 'MM2': []}}}
```

In [141…
```
df_advi = pd.DataFrame(records_advi)
df_advi['rho_rank'] = np.emath.logn(base, df_advi['h'])

I0_advi = df_advi.loc[(df_advi['S']==n_sample[-1]) & (df_advi['T']=="I0")]

kl_advi = df_advi.loc[(df_advi['S']==n_sample[-1]) & (df_advi['T']=="KL")]
var_advi = df_advi.loc[(df_advi['S']==n_sample[-1]) & (df_advi['T']=="Var")]
ll_advi = df_advi.loc[(df_advi['S']==n_sample[-1]) & (df_advi['T']=="LL")]
mm1_advi = df_advi.loc[(df_advi['S']==n_sample[-1]) & (df_advi['T']=="MM1")]
I_advi = df_advi.loc[(df_advi['S']==n_sample[-1]) & (df_advi['T']=="I")]

kl_advi = kl_advi.explode(['khat', 'ndx'])
var_advi = var_advi.explode(['khat', 'ndx'])
ll_advi = ll_advi.explode(['khat', 'ndx'])
I_advi = I_advi.explode(['khat', 'ndx'])
mm1_advi = mm1_advi.explode(['khat', 'ndx'])

original_advi = I_advi[['khat', 'ndx']].drop_duplicates()
```

In [142…
```
df_advi_ = df_advi.explode(['khat', "p_psis", 'ndx'])[['ndx', 'p_psis', 'khat', 'T']]
#df_ = df_[df_['p_psis']!=1]
#df_ = df_[df_['p_psis']>1e-5]
df_advi_ = df_advi_[df_advi_['T']!="MM2"]

df_advi_ = df_advi_.groupby(['ndx'])[['khat', 'p_psis']].min()

pd.set_option('display.max_rows', 500)

df_advi_ = df_advi_.reset_index()
df_advi_['y'] = [y_.tolist()[i][0] for i in df_advi_['ndx'].values]
```

In [143…
```
fpr_advi, tpr_advi, thresholds_advi = metrics.roc_curve(df_advi_['y'], df_advi_.p_psis, pos_label=1)
precision_advi, recall_advi, thresholds_pr_advi = metrics.precision_recall_curve(df_advi_['y'], df_advi_.p_psis)
```

In [145…
```
fig, ax = plt.subplots(1, 2, figsize=(9, 2))
ax[0].plot(fpr, tpr, linewidth=3, color="blue")
ax[0].text(0.5, 0.25, f"MCMC-AUROC: {metrics.auc(fpr, tpr):.3f}", color="blue")

ax[0].plot(fpr_advi, tpr_advi, color="red")
ax[0].text(0.5, 0.1, f"ADVI-AUROC: {metrics.auc(fpr_advi, tpr_advi):.3f}", color="red")

ax[0].set_xlim((0, 1))
ax[0].set_ylim((0, 1))
ax[0].set_title("ROC")

ax[1].plot(recall, precision, linewidth=3, color="blue")
ax[1].plot(recall_advi, precision_advi, color="red")
ax[1].text(0.5, 0.8, f"MCMC-AUPRC: {metrics.auc(recall, precision):.3f}", color="blue")
ax[1].text(0.5, 0.65, f"ADVI-AUPRC: {metrics.auc(recall_advi, precision_advi):.3f}", color="red")
ax[1].set_title("Precision-Recall")
ax[1].set_xlim((0, 1))
ax[1].set_ylim((0, 1))

_ = plt.savefig("roccurve_relu.pdf", bbox_inches='tight')
```

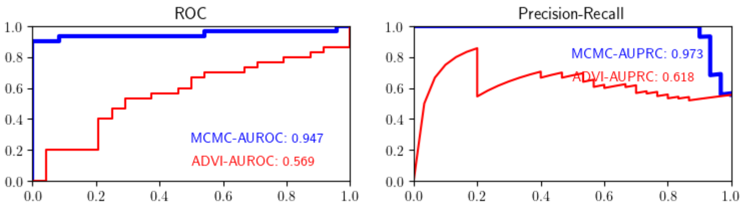

In [146…
```
def powerset(iterable):
    "powerset([1,2,3]) --> () (1,) (2,) (3,) (1,2) (1,3) (2,3) (1,2,3)"
    s = list(iterable)  # allows duplicate elements
    return chain.from_iterable(combinations(s, r) for r in range(len(s)+1))
```

In [149…
```
base = 4
h_vals = [base**-r for r in range(-2, 8)]

loo_output = []
success = []

for _ in tqdm(range(10)):
    loo_khat = {}
    reduced_ndx = {}
```

```
    high_khat_ndx = {}
    n_sample = [1000]
    records = []
    for n_samples in n_sample:
        reduced_ndx[n_samples] = {}
        high_khat_ndx[n_samples] = {}
        # print(f"Samples: {n_samples}")
        loo_khat[n_samples] = {}
        params_ = lr_model2.sample(n_samples)

        for h in h_vals:
            loo_khat[n_samples][h] = {}
            loo = adaptive_is_loo(lr_model2, batch, params_, h, variational=False)
            reduced_ndx[n_samples][h] = {}
            # print(f"rho={h}\n")
            for T in ["I0", "I",  "KL", "Var", "MM1", "MM2"]:
                loo_khat[n_samples][h][T] = np.array(loo[T]["khat"])

                records += [
                    {
                        "h": h,
                        "T": T,
                        "S": n_samples,
                        "khat": loo[T]["khat"],
                        "p_psis": loo[T]["p_psis"].numpy(),
                        "n>0.7": (np.where((loo[T]["khat"] > 0.7))[0]).shape,
                        "ndx": loo[T]["ndx"],
                    }
                ]
                reduced_ndx[n_samples][h][T] = [
                    loo[T]["ndx"][k] for k in np.where((loo[T]["khat"] <= 0.7))[0]
                ]
                # print(reduced_ndx[n_samples][h])

    df = pd.DataFrame(records)
    df["rho_rank"] = np.emath.logn(base, df["h"])
    __df__ = df.explode(["khat", "ndx"])[["ndx", "khat", "T"]]

    counts = {}
    counts["n"] = len(__df__[__df__["T"] != "I0"].ndx.unique())
    for T in transforms:
        ___df__ = __df__.loc[(__df__["T"] == T) & (__df__["khat"] < 0.7)]
        counts[T] = ___df__.groupby("ndx").min().shape[0]
    ___df__ = __df__.loc[
        ((__df__["T"] == "KL") | (__df__["T"] == "Var")) & (__df__["khat"] < 0.7)
    ]
    counts[("KL", "Var")] = ___df__.groupby("ndx").min().shape[0]
    ___df__ = __df__.loc[
        ((__df__["T"] == "MM1") | (__df__["T"] == "MM2") | (__df__["T"] == "LL"))
        & (__df__["khat"] < 0.7)
    ]
    counts[("MM1", "MM2")] = ___df__.groupby("ndx").min().shape[0]
    ___df__ = __df__.loc[
        ((__df__["T"] == "MM1") | (__df__["T"] == "MM2") |  (__df__["T"] == "KL") | (__df__["T"] == "Var"))
        & (__df__["khat"] < 0.7)
    ]
    counts[("KL", "Var", "MM1", "MM2")] = ___df__.groupby("ndx").min().shape[0]
    success += [counts]
```

```
  0%|          | 0/10 [00:00<?, ?it/s]
```

In [41]:
```
success = pd.DataFrame(success)
success_ = success.copy()
success_.iloc[:, 1:] = -success_.iloc[:, 1:].sub(success_.iloc[:, 0], axis=1)
```

In [43]:
```
success.agg(['mean','std'])
```

Out[43]:

	n	KL	Var	LL	MM1	MM2	(KL, Var)	(LL, MM1, MM2)	(KL, Var, LL, MM1, MM2)
**mean**	34.700000	17.900000	15.700000	12.700000	31.600000	32.300000	22.400000	33.900000	34.600000
**std**	2.830391	3.071373	2.406011	2.945807	2.988868	3.497618	3.272783	2.998148	3.025815

In [44]:
```
(-success.iloc[:, 1:].sub(success.iloc[:, 0], axis=0)).agg(['mean','std'])
```

Out[44]:

	KL	Var	LL	MM1	MM2	(KL, Var)	(LL, MM1, MM2)	(KL, Var, LL, MM1, MM2)
**mean**	16.800000	19.000000	22.000000	3.100000	2.400000	12.300000	0.800000	0.100000
**std**	1.619328	1.825742	1.563472	1.286684	1.646545	2.002776	0.632456	0.316228

In [46]:
```
_df = df.explode(['khat','ndx'])[['rho_rank', 'T', 'ndx', 'khat']]
_df['rho_rank'] *= -1
```

In [47]:
```
base_khat = _df[_df['T']=="I"][['ndx', 'khat']].drop_duplicates().to_dict(orient='records')
base_khat = {r['ndx']: r['khat'] for r in base_khat}
```

In [49]:
```
_df = _df[(_df["T"]!="I") & (_df["T"]!="I0")]
_df[r'$\log(\hat{k}/\hat{k}_0)$'] = _df.apply(lambda x: x.khat/base_khat[x.ndx], axis=1)
_df[r'$-log_4(\rho)$'] = _df['rho_rank']
```

```
/tmp/ipykernel_3246782/2716067429.py:2: SettingWithCopyWarning:
A value is trying to be set on a copy of a slice from a DataFrame.
Try using .loc[row_indexer,col_indexer] = value instead

See the caveats in the documentation: https://pandas.pydata.org/pandas-docs/stable/user_guide/indexing.html#returning-a-view-versus-a-copy
  _df[r'$\log(\hat{k}/\hat{k}_0)$'] = _df.apply(lambda x: x.khat/base_khat[x.ndx], axis=1)
/tmp/ipykernel_3246782/2716067429.py:3: SettingWithCopyWarning:
A value is trying to be set on a copy of a slice from a DataFrame.
Try using .loc[row_indexer,col_indexer] = value instead

See the caveats in the documentation: https://pandas.pydata.org/pandas-docs/stable/user_guide/indexing.html#returning-a-view-versus-a-copy
  _df[r'$-log_4(\rho)$'] = _df['rho_rank']
```

In [50]:
```
_df = _df[_df[r'$\log(\hat{k}/\hat{k}_0)$']<4]
```

In [51]:
```
from plotnine import ggplot, geom_line, geom_point, aes, stat_smooth, facet_wrap, theme, ylim
from plotnine.data import mtcars


plot = (
    ggplot(_df, aes(r'$-log_4(\rho)$', r'$\log(\hat{k}/\hat{k}_0)$', color="T"))
    + geom_line()
    + facet_wrap("ndx", ncol=4)
    + theme(figure_size=(15, 16), legend_position=(.5, .0), legend_direction='horizontal') + ylim((0, 2))

)
```

```
plot.show()
plot.save("rho_rank_relu.pdf")
```

```
/home/josh/workspace/readmission/env/lib/python3.10/site-packages/plotnine/geoms/geom_path.py:100: PlotnineWarning: geom_path: Removed 1 rows containing missing values.
```

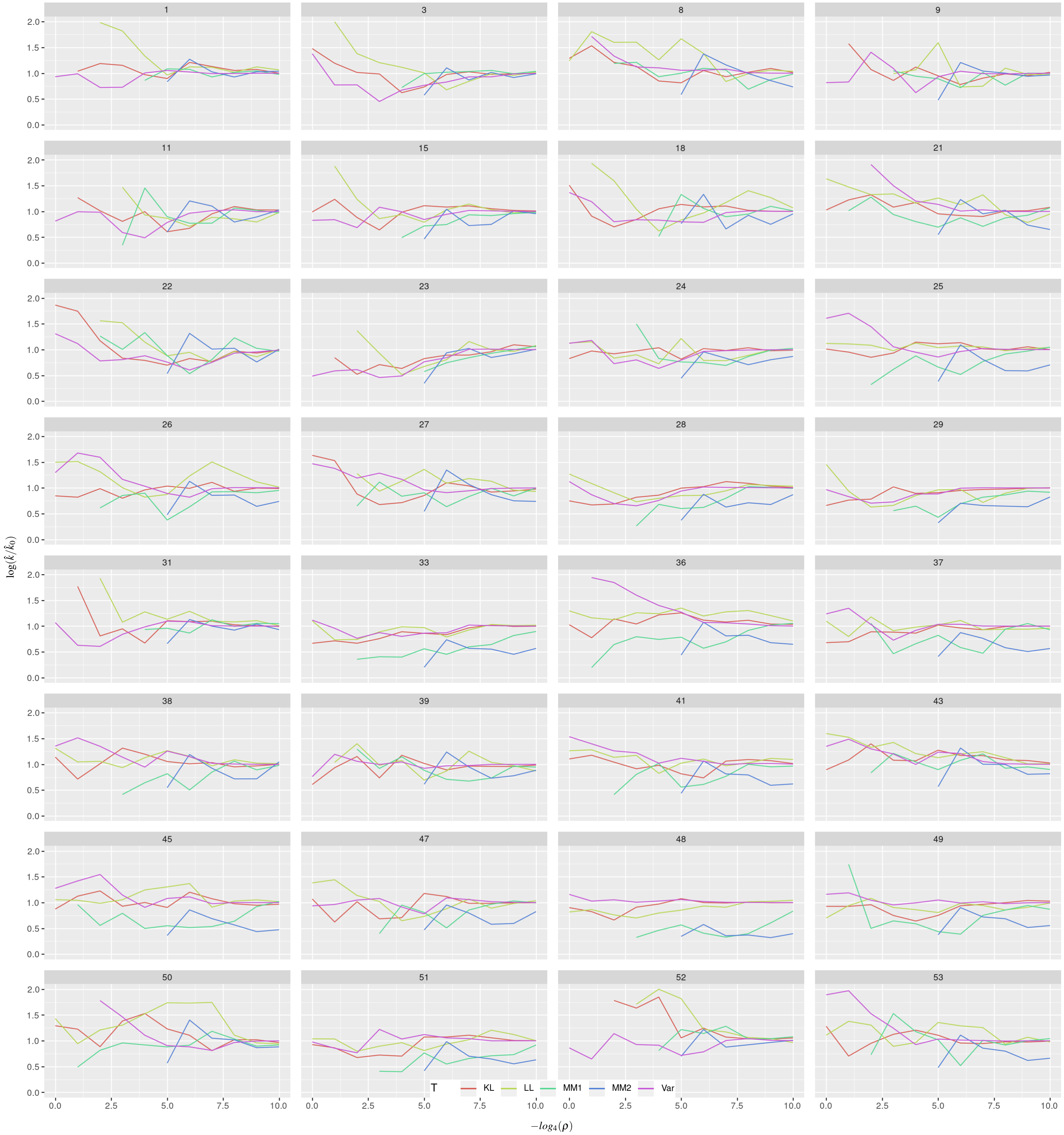


```
/home/josh/workspace/readmission/env/lib/python3.10/site-packages/plotnine/ggplot.py:606: PlotnineWarning: Saving 15 x 16 in image.
/home/josh/workspace/readmission/env/lib/python3.10/site-packages/plotnine/ggplot.py:607: PlotnineWarning: Filename: rho_rank.pdf
/home/josh/workspace/readmission/env/lib/python3.10/site-packages/plotnine/geoms/geom_path.py:100: PlotnineWarning: geom_path: Removed 1 rows containing missing values.
```

In [ ]:

In [ ]:
