# Supplementary material for: Gradient-flow adaptive importance sampling for Bayesian leave one out cross-validation for sigmoidal classification models
Source: ArXiv. 2024 Feb 13:arXiv:2402.08151v1. Preprint. [Version 1] (PMC11071546)
Supplement: Supplementary file 1 [file NIHPP2402.08151v1-supplement-1.pdf]

Gradient-flow adaptive importance sampling for Bayesian LOO (Supplementary Material)

Joshua C. Chang[1] **Xiangting Li**[2] **Shixin Xu**[3] **Hao-ren Yao**[1] **Julia Porcino**[1] **Carson C. Chow**[4]

[1]NIH Clinical Center, Rehabilitation Medicine, Epidemiology and Biostatistics Section, Bethesda MD, USA
[2]UCLA Department of Computational Medicine, Los Angeles CA, USA
[3]Data Science Research Center, Duke Kunshan University, Kunshan, Jiangsu, China
[4]NIH NIDDK, Laboratory of Biological Modeling, Bethesda MD, USA

S.1 VARIATIONAL PROBLEMS

S.1.1 KL DIVERGENCE

For convenience, we write the cross-entropy in the forward-transformation form, so that

$$\begin{aligned} H\left(\pi(\boldsymbol{\phi}|\mathcal{D}^{(-i)}), \pi_{\boldsymbol{\phi}}(\boldsymbol{\phi}|\mathcal{D})\right) &= -\int \nu_i(\boldsymbol{\phi})\pi(\boldsymbol{\phi}|\mathcal{D}) \log \frac{\pi(T_i^{-1}(\boldsymbol{\phi})|\mathcal{D})}{\mathcal{J}_i(T_i^{-1}(\boldsymbol{\phi}))} \mathrm{d}\boldsymbol{\phi} \\ &= -\int \nu_i(T_i(\boldsymbol{\theta}))\pi(T_i(\boldsymbol{\theta})|\mathcal{D}) \log \frac{\pi(\boldsymbol{\theta}|\mathcal{D})}{\mathcal{J}_i(\boldsymbol{\theta})} \mathcal{J}_{T_i}(\boldsymbol{\theta}) \mathrm{d}\boldsymbol{\theta}. \end{aligned} \tag{S.1}$$

Now for an arbitrary test function with vanishing boundary conditions, ξ,

$$\begin{aligned} &\int \frac{\delta H\left(\pi(\boldsymbol{\theta}|\mathcal{D}^{(-i)}), \pi_{\boldsymbol{\phi}}(\boldsymbol{\theta}|\mathcal{D})\right)}{\delta T_i} \xi(\boldsymbol{\theta}) \mathrm{d}\boldsymbol{\theta} \\ &\quad = -\lim_{\varepsilon\to 0} \frac{\mathrm{d}}{\mathrm{d}\varepsilon} \int \nu_i(T_i(\boldsymbol{\theta}) + \varepsilon\xi(\boldsymbol{\theta}))\pi(T_i(\boldsymbol{\theta}) + \varepsilon\xi(\boldsymbol{\theta})|\mathcal{D}) \log \frac{\pi(\boldsymbol{\theta}|\mathcal{D})}{|\nabla T_i(\boldsymbol{\theta}) + \varepsilon\nabla\xi(\boldsymbol{\theta})|} |\nabla T_i(\boldsymbol{\theta}) + \varepsilon\nabla\xi| \mathrm{d}\boldsymbol{\theta} \\ &\quad = -\int \nabla\left\{\pi(\boldsymbol{\theta}|\mathcal{D})\nu_i(\boldsymbol{\theta})\right\}\big|_{\boldsymbol{\theta}=T_i(\boldsymbol{\theta})} \mathcal{J}_i(\boldsymbol{\theta}) \log \frac{\pi(\boldsymbol{\theta}|\mathcal{D})}{\mathcal{J}_i(\boldsymbol{\theta})} \cdot \xi(\boldsymbol{\theta}) \mathrm{d}\boldsymbol{\theta} \\ &\qquad - \int \nu_i(T_i(\boldsymbol{\theta}))\pi(T_i(\boldsymbol{\theta})|\mathcal{D}) \left[\mathcal{J}_{T_i}(\boldsymbol{\theta}) \log \frac{\pi(\boldsymbol{\theta}|\mathcal{D})}{\mathcal{J}_{T_i}(\boldsymbol{\theta})} - \mathcal{J}_{T_i}(\boldsymbol{\theta})\right] \mathrm{tr}\left(\mathbf{J}_i^{-1}(\boldsymbol{\theta})\nabla\xi(\boldsymbol{\theta})\right) \mathrm{d}\boldsymbol{\theta}. \end{aligned} \tag{S.2}$$

Re-arranging the trace term,

$$\mathrm{tr}\left[\mathbf{J}_i^{-1}\nabla\xi(\boldsymbol{\theta})\right] = \sum_q \sum_r (\mathbf{J}_i^{-1})_{qr} (\nabla\xi)_{rq} = \sum_r \sum_q \frac{\partial \xi_r}{\partial \theta_q} (\mathbf{J}_i^{-1}(\boldsymbol{\theta}))_{qr}, \tag{S.3}$$

and integrating each term by parts,

$$\begin{aligned} &\int \frac{\delta H\left(\pi(\boldsymbol{\theta}|\mathcal{D}^{(-i)}), \pi_{\boldsymbol{\phi}}(\boldsymbol{\theta}|\mathcal{D})\right)}{\delta T_i} \xi(\boldsymbol{\theta}) \mathrm{d}\boldsymbol{\theta} \\ &\quad = -\int \nabla\left\{\pi(\boldsymbol{\theta}|\mathcal{D})\nu_i(\boldsymbol{\theta})\right\}\big|_{\boldsymbol{\theta}=T_i(\boldsymbol{\theta})} \mathcal{J}_i(\boldsymbol{\theta}) \log \frac{\pi(\boldsymbol{\theta}|\mathcal{D})}{\mathcal{J}_i(\boldsymbol{\theta})} \cdot \xi(\boldsymbol{\theta}) \mathrm{d}\boldsymbol{\theta} \\ &\qquad + \sum_r \nabla \cdot \int \frac{\partial}{\partial \theta_q} \left\{\nu_i(T_i(\boldsymbol{\theta}))\pi(T_i(\boldsymbol{\theta})|\mathcal{D}) \left[\mathcal{J}_{T_i}(\boldsymbol{\theta}) \log \frac{\pi(\boldsymbol{\theta}|\mathcal{D})}{\mathcal{J}_{T_i}(\boldsymbol{\theta})} - \mathcal{J}_{T_i}(\boldsymbol{\theta})\right] (\mathbf{J}_i^{-1}(\boldsymbol{\theta}))_{qr}\right\} \mathbf{e}_r \cdot \xi \mathrm{d}\boldsymbol{\theta}. \end{aligned} \tag{S.4}$$

So, the Euler-Lagrange equation satisfies

$$\begin{aligned}\frac{\delta \mathcal{H}}{\delta T_i} &= -\nabla(\pi(\boldsymbol{\theta}|\mathcal{D})\nu_i(\boldsymbol{\theta}))\big|_{\boldsymbol{\theta}=T_i(\boldsymbol{\theta})}\mathcal{J}_i(\boldsymbol{\theta})\log\frac{\pi(\boldsymbol{\theta}|\mathcal{D})}{\mathcal{J}_i(\boldsymbol{\theta})} \\ &\quad + \sum_r \nabla\cdot\left\{\nu_i(T_i(\boldsymbol{\theta}))\pi(T_i(\boldsymbol{\theta})|\mathcal{D})\left[\mathcal{J}_{T_i}(\boldsymbol{\theta})\log\frac{\pi(\boldsymbol{\theta}|\mathcal{D})}{\mathcal{J}_{T_i}(\boldsymbol{\theta})} - \mathcal{J}_{T_i}(\boldsymbol{\theta})\right](\mathbf{J}_i^{-1}(\boldsymbol{\theta}))_{qr}\right\}\mathbf{e}_r \\ &= 0. \end{aligned} \tag{S.5}$$

We approximate T_i using the single-step (of size $0 < h \ll 1$) forward Euler update under the initial condition $T_i(\boldsymbol{\theta}, 0) = \boldsymbol{\theta}$,

$$T_i(\boldsymbol{\theta}) \approx T_i(\boldsymbol{\theta},0) - h\frac{\delta H\left(\pi(\boldsymbol{\theta}|\mathcal{D}^{(-i)}), \pi_{\boldsymbol{\phi}}(\boldsymbol{\theta}|\mathcal{D})\right)}{\delta T_i}\Big|_{T_i(\boldsymbol{\theta})=\boldsymbol{\theta}} = \boldsymbol{\theta} + h\pi(\boldsymbol{\theta}|\mathcal{D})\nabla\left(\frac{1}{\ell(\boldsymbol{\theta}|\mathbf{x}_i, y_i)}\right), \tag{S.6}$$

where we have absorbed the unknown normalizing constant into h.

S.1.2 VARIANCE

The variance of the transformed importance sampling estimator of Eq. 13 is specific to the target function. We examine the variance in computing the expectation of a function $f(\boldsymbol{\theta})$ which is related to the variance of the individual element

$$\begin{aligned}\mathrm{Var}\left[\nu_i(\boldsymbol{\phi})\mathcal{J}_{T_i}(T_i^{-1}(\boldsymbol{\phi}))\frac{\pi(\boldsymbol{\phi}|\mathcal{D})}{\pi(T_i^{-1}(\boldsymbol{\phi})|\mathcal{D})}f(\boldsymbol{\theta})\right] &= \int\left[\nu_i(\boldsymbol{\phi})\frac{\pi(\boldsymbol{\phi}|\mathcal{D})}{\pi_{\boldsymbol{\phi}}(\boldsymbol{\phi}|\mathcal{D})}f(\boldsymbol{\theta})\right]^2\pi_{\boldsymbol{\phi}}(\boldsymbol{\phi}|\mathcal{D})\mathrm{d}\boldsymbol{\phi} \\ &\quad - \left\{\int\left[\nu_i(\boldsymbol{\phi})\frac{\pi(\boldsymbol{\phi}|\mathcal{D})}{\pi_{\boldsymbol{\phi}}(\boldsymbol{\phi}|\mathcal{D})}f(\boldsymbol{\theta})\right]\pi_{\boldsymbol{\phi}}(\boldsymbol{\phi}|\mathcal{D})\mathrm{d}\boldsymbol{\phi}\right\}^2 \\ &= \int\frac{[\nu_i(\boldsymbol{\phi})\pi(\boldsymbol{\phi}|\mathcal{D})f(\boldsymbol{\theta})]^2}{\pi_{\boldsymbol{\phi}}(\boldsymbol{\phi}|\mathcal{D})}\mathrm{d}\boldsymbol{\phi} + \text{constant relative to } T_i \end{aligned} \tag{S.7}$$

Note that if one plugs in $f(\boldsymbol{\theta}) = p_i(\boldsymbol{\theta})$ into Eq. S.7, then when $y_i = 1$ the overall functional loses dependence on $\ell(\boldsymbol{\theta}|\mathbf{x}_i, y_i)$ because ν_i and $p_i(\boldsymbol{\theta})$ cancel. For this reason, in order to optimize with respect to the variance of the prediction, one can optimize against the symmetric function

$$f_i(\boldsymbol{\theta}) = p_i(\boldsymbol{\theta})^{1-y_i}(1 - p_i(\boldsymbol{\theta})^{y_i}.$$

Writing this expression in terms of the forward transformation and doing a change of variables $\mathrm{d}\boldsymbol{\phi} = \mathcal{J}_{T_i}(\boldsymbol{\theta})\mathrm{d}\boldsymbol{\theta}$, we write the functional to minimize

$$\begin{aligned}\mathcal{V}[T_i] &= \int\frac{g(T_i(\boldsymbol{\theta}))}{\pi_{\boldsymbol{\phi}}(T_i(\boldsymbol{\theta})|\mathcal{D})}\mathcal{J}_{T_i}(\boldsymbol{\theta})\mathrm{d}\boldsymbol{\theta} \\ &= \int\frac{g(T_i(\boldsymbol{\theta}))}{\pi(\boldsymbol{\theta}|\mathcal{D})}\mathcal{J}_i^2(\boldsymbol{\theta})\mathrm{d}\boldsymbol{\theta} \end{aligned} \tag{S.8}$$

$$g(\boldsymbol{\theta}) = (\nu_i(\boldsymbol{\theta})\pi(\boldsymbol{\theta}|\mathcal{D})f_i(\boldsymbol{\theta}))^2. \tag{S.9}$$

Computing the first variation of $\mathcal{V}$ with respect to T_i, using Gateaux differentiation,

$$\begin{aligned}\delta\mathcal{V} &= \lim_{\varepsilon\to 0}\frac{\mathrm{d}}{\mathrm{d}\varepsilon}\int\frac{g(T_i(\boldsymbol{\theta})+\varepsilon\xi)}{\pi(\boldsymbol{\theta}|\mathcal{D})}\left|\nabla T_i + \varepsilon\nabla\xi\right|^2\mathrm{d}\boldsymbol{\theta} \\ &= \int\left\{\frac{\nabla g(T_i(\boldsymbol{\theta}))\mathcal{J}_i^2(\boldsymbol{\theta})}{\pi(\boldsymbol{\theta}|\mathcal{D})}\cdot\xi(\boldsymbol{\theta}) + 2\frac{g(T_i(\boldsymbol{\theta}))\mathcal{J}_i^2(\boldsymbol{\theta})}{\pi(\boldsymbol{\theta}|\mathcal{D})}\mathrm{tr}\left(\mathbf{J}_i^{-1}(\boldsymbol{\theta})\nabla\xi(\boldsymbol{\theta})\right)\right\}\mathrm{d}\boldsymbol{\theta} \\ &= \int\left\{\frac{\nabla g(T_i(\boldsymbol{\theta}))\mathcal{J}_i^2(\boldsymbol{\theta})}{\pi(\boldsymbol{\theta}|\mathcal{D})} - 2\sum_r\nabla\cdot\left[\frac{g(T_i(\boldsymbol{\theta}))\mathcal{J}_i^2(\boldsymbol{\theta})}{\pi(\boldsymbol{\theta}|\mathcal{D})}\mathbf{J}_i^{-1}(\boldsymbol{\theta})_{,r}\right]\mathbf{e}_r\right\}\cdot\xi(\boldsymbol{\theta})\mathrm{d}\boldsymbol{\theta}. \end{aligned} \tag{S.10}$$

So the Euler-Lagrange equation for minimizing the variance is

$$\frac{\delta\mathcal{V}}{\delta T_i} = \frac{\nabla g(T_i(\boldsymbol{\theta}))\mathcal{J}_i^2(\boldsymbol{\theta})}{\pi(\boldsymbol{\theta}|\mathcal{D})} - 2\sum_r\nabla\cdot\left[\frac{g(T_i(\boldsymbol{\theta}))\mathcal{J}_i^2(\boldsymbol{\theta})}{\pi(\boldsymbol{\theta}|\mathcal{D})}\mathbf{J}_i^{-1}(\boldsymbol{\theta})_{,r}\right]\mathbf{e}_r = 0. \tag{S.11}$$

For $T_i(\boldsymbol{\theta}) = \boldsymbol{\theta}$, the functional derivative is

$$
\begin{aligned}
\frac{\delta \mathcal{V}}{\delta T_i}\Big|_{T_i=\boldsymbol{\theta}} &= \frac{\nabla g(\boldsymbol{\theta})}{\pi(\boldsymbol{\theta}|\mathcal{D})} - 2\nabla\left(\frac{g(\boldsymbol{\theta})}{\pi(\boldsymbol{\theta}|\mathcal{D})}\right) \\
&= \frac{\nabla g(\boldsymbol{\theta})}{\pi(\boldsymbol{\theta}|\mathcal{D})} - 2\frac{\nabla g(\boldsymbol{\theta})}{\pi(\boldsymbol{\theta}|\mathcal{D})} + 2\frac{g\nabla\pi(\boldsymbol{\theta})}{\pi^2(\boldsymbol{\theta}|\mathcal{D})} \\
&= -\frac{2\nu_i(\boldsymbol{\theta})\pi(\boldsymbol{\theta}|\mathcal{D})f_i(\boldsymbol{\theta})\nabla(\nu_i(\boldsymbol{\theta})\pi(\boldsymbol{\theta})f_i(\boldsymbol{\theta}))}{\pi(\boldsymbol{\theta}|\mathcal{D})} + 2\frac{g\nabla\pi(\boldsymbol{\theta})}{\pi^2(\boldsymbol{\theta}|\mathcal{D})} \\
&= -2\nu_i(\boldsymbol{\theta})f_i(\boldsymbol{\theta})\nabla(\nu_i(\boldsymbol{\theta})\pi(\boldsymbol{\theta}|\mathcal{D})f_i(\boldsymbol{\theta})) + 2\nu_i^2(\boldsymbol{\theta})f_i^2(\boldsymbol{\theta})\nabla\pi(\boldsymbol{\theta}|\mathcal{D}) \\
&= -2\nu_i(\boldsymbol{\theta})f_i(\boldsymbol{\theta})\pi(\boldsymbol{\theta}|\mathcal{D})\nabla\big[\nu_i(\boldsymbol{\theta})f_i(\boldsymbol{\theta})\big]
\end{aligned}
\tag{S.12}
$$

S.2 SIGMOIDAL MODELS

For these models one may use the chain rule to write the gradient,

$$\nabla\ell(\boldsymbol{\theta}|\mathbf{x}, y) = \ell(\boldsymbol{\theta}|\mathbf{x}, y)\nabla\log\ell(\boldsymbol{\theta}|\mathbf{x}, y) \tag{S.13}$$

$$\nabla\log\ell(\boldsymbol{\theta}|\mathbf{x}, y) = \underbrace{[y(1-\sigma(\mu)) - (1-y)\sigma(\mu)]}_{(\log\ell)'}\nabla\mu \tag{S.14}$$

and Hessian

$$
\begin{aligned}
\nabla\nabla\ell(\boldsymbol{\theta}|\mathbf{x}, y) &= \ell(\boldsymbol{\theta}|\mathbf{x}, y)\nabla\nabla\log\ell(\boldsymbol{\theta}|\mathbf{x}, y) \\
&\quad + \ell(\boldsymbol{\theta}|\mathbf{x}, y)\nabla\log\ell(\boldsymbol{\theta}|\mathbf{x}, y)\nabla\log\ell(\boldsymbol{\theta}|\mathbf{x}, y)
\end{aligned}
\tag{S.15}
$$

$$
\begin{aligned}
\nabla\nabla\log\ell(\boldsymbol{\theta}|\mathbf{x}, y) &= [y(1-\sigma(\mu)) - (1-y)\sigma(\mu)]\nabla\nabla\mu \\
&\quad \underbrace{-\sigma(\mu)(1-\sigma(\mu))}_{(\log\ell)''}\nabla\mu\nabla\mu
\end{aligned}
\tag{S.16}
$$

for the likelihood function as a function of the gradient and Hessian of μ.

For the sake of space, we drop all indices i and let $\pi = \pi(\boldsymbol{\theta}|\mathbf{x})$ and $\ell = \ell(\boldsymbol{\theta}|\mathbf{x}, y)$ All three transformations are of the form $T(\boldsymbol{\theta}) = \boldsymbol{\theta} + Q(\boldsymbol{\theta})$

S.2.1 RELU WITH ONE HIDDEN LAYER

Ordering the parameters $(W_1)_{11}, \ldots, (W_1)_{1p}, (W_1)_{21}, \ldots, (W_1)_{2p}, (W_1)_{d1}, \ldots, (W_1)_{dp}, (W_1)_1, (W_2)_1, \ldots, (W_2)_d, b_2$, the Hessian of μ takes the form

$$\nabla\nabla^{\intercal}\mu = \begin{pmatrix} \mathbf{0} & \mathbf{M} & \mathbf{0} \\ \mathbf{M}^{\intercal} & \mathbf{0} & \mathbf{0} \\ \mathbf{0} & \mathbf{0} & 0 \end{pmatrix}, \tag{S.17}$$

where $\mathbf{M}$ encodes the mixed partial derivatives of μ with respect to the elements of $\mathbf{W}_1$ and $\mathbf{W}_2$,

$$\frac{\partial^2\mu}{\partial(W_1)_{ij}(W_2)_{1k}} = \delta_{ik}a'((z_1)_i)x_j. \tag{S.18}$$

The $dp \times d$ matrix $\mathbf{M}$ takes the form

$$\mathbf{M} = \begin{pmatrix} \mathbf{u}_1 & \mathbf{0} & \mathbf{0} & \cdots & \mathbf{0} \\ \mathbf{0} & \mathbf{u}_2 & \mathbf{0} & \cdots & \mathbf{0} \\ \mathbf{0} & \mathbf{0} & \mathbf{u}_3 & \ddots & \vdots \\ \vdots & \vdots & \ddots & \ddots & \vdots \\ \mathbf{0} & \cdots & \cdots & \cdots & \mathbf{u}_d \end{pmatrix} \tag{S.19}$$

where each column vector

$$\mathbf{u}_k = \begin{pmatrix} a'((z_1)_k)x_1 & a'((z_1)_k)x_2 & \cdots & a'((z_1)_k)x_p \end{pmatrix}^{\intercal} = a'((z_1)_k)\mathbf{x} \tag{S.20}$$

is potentially sparse.

S.2.1.1 Hessian decomposition

Suppose that λ is an eigenvalue of $\nabla\nabla^\intercal\mu$, corresponding to the eigenvector $\boldsymbol{\psi} = \begin{pmatrix}\boldsymbol{\psi}_1^\intercal & \boldsymbol{\psi}_2^\intercal & \boldsymbol{\psi}_3^\intercal\end{pmatrix}^\intercal$. Then,

$$\mathbf{M}\boldsymbol{\psi}_2 = \lambda\boldsymbol{\psi}_1 \tag{S.21}$$
$$\mathbf{M}^\intercal\boldsymbol{\psi}_1 = \lambda\boldsymbol{\psi}_2 \tag{S.22}$$
$$\boldsymbol{\psi}_3 = \mathbf{0}. \tag{S.23}$$

Left-multiplying Eq. S.21 by $\mathbf{M}^\intercal$ and Eq. S.22 by $\mathbf{M}$, one finds that

$$\mathbf{M}^\intercal\mathbf{M}\boldsymbol{\psi}_2 = \lambda\mathbf{M}^\intercal\psi_1 = \lambda^2\boldsymbol{\psi}_2 \tag{S.24}$$
$$\mathbf{M}\mathbf{M}^\intercal\boldsymbol{\psi}_1 = \lambda\mathbf{M}\mathbf{M}^\intercal\boldsymbol{\psi}_2 = \lambda^2\boldsymbol{\psi}_1. \tag{S.25}$$

Eq. S.24 implies that solutions for $\boldsymbol{\psi}_2$ are eigenvectors of $\mathbf{M}^\intercal\mathbf{M}$ and the solutions for $\boldsymbol{\psi}_1$ are eigenvectors of $\mathbf{M}\mathbf{M}^\intercal$.

The matrix $\mathbf{M}^\intercal\mathbf{M}$ is the diagonal matrix diag($|\mathbf{u}_k|^2$), so the non-zero eigenvalues are each of $\pm|u_k|$, and the solutions for $\boldsymbol{\psi}_2$ are proportional to elements of the d dimensional standard basis.

It is easy to see that the eigenvectors of $\mathbf{M}\mathbf{M}^T$, which we denote $\tilde{\mathbf{u}}_k$, are related to the column vectors of $\mathbf{M}$,

$$\tilde{\mathbf{u}}_k = \big(\overbrace{0 \quad \ldots \quad 0}^{(k-1)p \text{ zeros}} \quad \mathbf{u}_k^\intercal \quad \overbrace{0 \quad \ldots \quad 0}^{(d-k)p \text{ zeros}}\big)^\intercal. \tag{S.26}$$

Overall, each eigenvector for $\nabla\nabla^\intercal\mu$ takes the form $\begin{pmatrix}\tilde{\mathbf{u}}_k^\intercal & \gamma\mathbf{e}_k^\intercal & 0\end{pmatrix}^\intercal$ for some constant γ. By Eq. S.24,

$$\mathbf{M}\gamma\mathbf{e}_k = \gamma\tilde{\mathbf{u}}_k = \lambda\tilde{\mathbf{u}}_k,$$

so $\gamma = \lambda$. Hence, by spectral theorem, we may write the decomposition for $\nabla\nabla^\intercal\mu$,

$$\nabla\nabla^\intercal\mu = \sum_{k=1}^{d}\left[|\mathbf{u}_k|\underbrace{\begin{pmatrix}\tilde{\mathbf{u}}_k/\sqrt{2|\mathbf{u}_k|^2}\\ \mathbf{e}_k/\sqrt{2}\\ 0\end{pmatrix}}_{\mathbf{v}_{k+}}\begin{pmatrix}\tilde{\mathbf{u}}_k^\intercal/\sqrt{2|\mathbf{u}_k|^2} & \mathbf{e}_k^\intercal/\sqrt{2} & 0\end{pmatrix} - |\mathbf{u}_k|\underbrace{\begin{pmatrix}\tilde{\mathbf{u}}_k/\sqrt{2|\mathbf{u}_k|^2}\\ -\mathbf{e}_k/\sqrt{2}\\ 0\end{pmatrix}}_{\mathbf{v}_{k-}}\begin{pmatrix}\tilde{\mathbf{u}}_k^\intercal/\sqrt{2|\mathbf{u}_k|^2} & -\mathbf{e}_k^\intercal/\sqrt{2} & 0\end{pmatrix}\right]. \tag{S.27}$$

S.2.1.2 Overall model Jacobians

To assemble the Jacobian of the overall transformation, we need to diagonalize the gradient outer product simultaneously with $\mathbf{M}$. The gradient terms follow

$$\frac{\partial\mu}{\partial b_2} = 1 \tag{S.28}$$
$$\frac{\partial\mu}{\partial(W_2)_{1k}} = a((z_1)_k) \tag{S.29}$$
$$\frac{\partial\mu}{\partial(W_1)_{jk}} = (W_2)_j a'((z_1)_j)x_k \tag{S.30}$$

so that

$$\nabla\mu = \begin{pmatrix}(W_2)_1\mathbf{u}_1^\intercal & (W_2)_2\mathbf{u}_2^\intercal & \cdots & (W_2)_d\mathbf{u}_d^\intercal & a((z_1)_1) & a((z_1)_2) & \cdots & a((z_1)_d) & 1\end{pmatrix}^\intercal \tag{S.31}$$

We then decompose the gradient into the eigenspace of the Hessian,

$$\begin{aligned}\nabla\mu &= \sum_{k=1}^{d}\left[\frac{(W_2)_k|\mathbf{u}_k| + a((z_1)_k)}{\sqrt{2}}\mathbf{v}_{k+} + \frac{(W_2)_k|\mathbf{u}_k| - a((z_1)_k)}{\sqrt{2}}\mathbf{v}_{k-}\right] + \underbrace{\begin{pmatrix}0 & \cdots & 0 & 1\end{pmatrix}^\intercal}_{\mathbf{e}_{dp+d+1}}\\ &= \mathbf{V}\mathbf{c}\end{aligned} \tag{S.32}$$

where $\mathbf{V}$ is the matrix where the columns are the eigenvectors

$$\mathbf{V} = \begin{pmatrix} \mathbf{v}_{1+} & \mathbf{v}_{1-} & \mathbf{v}_{2+} & \mathbf{v}_{2-} & \cdots & \mathbf{v}_{d+} & \mathbf{v}_{d-} & \mathbf{e}_{dp+d+1} \end{pmatrix}, \tag{S.33}$$

and $\mathbf{c}$ is a column vector corresponding to the coefficients in Eq. S.32.

Putting it all together, the Jacobians of the transformation are, for the log-likelihood descent tranformation,

$$\begin{aligned} \mathbf{I} - h\nabla\nabla^{\intercal}\log\ell = \mathbf{I} - h\Bigg\{ & [y(1-\sigma(\mu)) - (1-y)\sigma(\mu)] \overbrace{\left[\sum_{k=1}^{d}\left(|\mathbf{u}_k|\mathbf{v}_{k+}\mathbf{v}_{k+}^{\intercal} - |\mathbf{u}_k|\mathbf{v}_{k-}\mathbf{v}_{k-}^{\intercal}\right)\right]}^{\nabla\nabla^{\intercal}\mu} \\ & - \sigma(\mu)(1-\sigma(\mu))\mathbf{V}\mathbf{c}\mathbf{c}^{\intercal}\mathbf{V}^{\intercal}\Bigg\}. \end{aligned} \tag{S.34}$$

To compute the determinant of Eq. S.34 we first make two observations. First, two similar matrices have the same determinant, so that

$$\begin{aligned} \det\left[\mathbf{I} - t\nabla\nabla^{\intercal}\log\ell\right] &= \det\left[\mathbf{I} - h\left[[y(1-\sigma(\mu)) - (1-y)\sigma(\mu)]\mathbf{V}\mathrm{diag}(\lambda_i)\mathbf{V}^{\intercal} - \sigma(\mu)(1-\sigma(\mu))\mathbf{V}\mathbf{c}\mathbf{c}^{\intercal}\mathbf{V}^{\intercal}\right]\right] \\ &= \det\left[\underbrace{\mathbf{I} - h\left[[y(1-\sigma(\mu)) - (1-y)\sigma(\mu)]\mathrm{diag}(\lambda_i)\right]}_{\text{diagonal terms from } \mathbf{I} \text{ and } \nabla\nabla^{\intercal}\mu} + h\underbrace{\sigma(\mu)(1-\sigma(\mu))\mathbf{c}\mathbf{c}^{\intercal}}_{\text{from gradient outer product term}}\right], \end{aligned} \tag{S.35}$$

and second that the non-diagonal elements of the matrix in Eq. S.35 are rank-one so that

$$\begin{aligned} \mathcal{J}_{T^{\mathrm{LL}}}(\boldsymbol{\theta}) = \prod_{j=1}^{d} & \left(1 - h^2[y(1-\sigma(\mu)) - (1-y)\sigma(\mu)]^2|\mathbf{u}_j|^2\right) \\ \times & \left(1 + h\sigma(\mu)(1-\sigma(\mu))\left[\sum_{k=1}^{d}\left[\frac{[(W_2)_k|\mathbf{u}_k| + a((z_1)_k)]^2}{2[1 - h[y(1-\sigma(\mu)) - (1-y)\sigma(\mu)]|\mathbf{u}_k|]}\right.\right.\right. \\ & \left.\left.\left. + \frac{[(W_2)_k|\mathbf{u}_k| - a((z_1)_k)]^2}{2[1 + h[y(1-\sigma(\mu)) - (1-y)\sigma(\mu)]|\mathbf{u}_k|]}\right] + 1\right]\right). \end{aligned} \tag{S.36}$$
